# Identify the underlying true model from other models for clinical practice using model performance measures

**DOI:** 10.1186/s12874-025-02457-w

**Published:** 2025-01-09

**Authors:** Yan Li

**Affiliations:** School of Mathematical Sciences, Xiamen University, Xiamen, 361005 People’s Republic of China

**Keywords:** Clinical risk prediction model, Outcome generation true model, Model performance measures, Cardiovascular disease

## Abstract

**Objective:**

To assess whether the outcome generation true model could be identified from other candidate models for clinical practice with current conventional model performance measures considering various simulation scenarios and a CVD risk prediction as exemplar.

**Study design and setting:**

Thousands of scenarios of true models were used to simulate clinical data, various candidate models and true models were trained on training datasets and then compared on testing datasets with 25 conventional use model performance measures. This consists of univariate simulation (179.2k simulated datasets and over 1.792 million models), multivariate simulation (728k simulated datasets and over 8.736 million models) and a CVD risk prediction case analysis.

**Results:**

True models had overall C statistic and 95% range of 0.67 (0.51, 0.96) across all scenarios in univariate simulation, 0.81 (0.54, 0.98) in multivariate simulation, 0.85 (0.82, 0.88) in univariate case analysis and 0.85 (0.82, 0.88) in multivariate case analysis. Measures showed very clear differences between the true model and flip-coin model, little or none differences between the true model and candidate models with extra noises, relatively small differences between the true model and proxy models missing causal predictors.

**Conclusion:**

The study found the true model is not always identified as the “outperformed” model by current conventional measures for binary outcome, even though such true model is presented in the clinical data. New statistical approaches or measures should be established to identify the casual true model from proxy models, especially for those in proxy models with extra noises and/or missing causal predictors.

**Supplementary Information:**

The online version contains supplementary material available at 10.1186/s12874-025-02457-w.

## Introduction

Clinical risk Prediction Models (CPM) help clinicians to identify high risk patients of certain clinical outcomes and assist clinical decision making. E.g., NICE guideline recommended to prescribe statins to patients with QRISK3 predicted cardiovascular disease (CVD) risk above 10% [1, 2]. These models could be developed using either statistical model [2] or machine learning [3]. They were assessed by model performance measurements such as discrimination (ability of model to discriminate high risk patients from low risk) and calibration (agreement between the predicted risk and the observed events rate). Usually, the model with the highest performance was applied in the clinical practice [4, 5]. Previous studies [3, 6, 7] found that models with similar model performance could predict contradictory risks to the same individual patient resulting contradictory clinical decision to that patient. It was suggested more causal predictors should be considered [3, 8]. While the “most causal” model would be the underlying true model that generates the outcome variable, i.e., the outcome generation true model. For example, such true model could be a pre-defined mathematical formula which contains all the causal predictors and their true effects on the outcome variable. However, if such true model does exist in the collected clinical data, could it be identified from other candidate models using current conventional model performance measures? Since it is the underlying true model that describes how exact outcome variable depends on all the causal predictors, it should be the preferred model being used for the clinical practice comparing to other proxy models which contain noises or missing causal predictors. Peter et al. [9] has studied the change of a few performance measures when the true model has more predictors than the proxy model. Yet, other scenarios, such as when the proxy model has more predictors or the causal predictor was replaced by the proxy were not covered. This study assessed whether the true model could be identified from other proxy models for clinical practice using more conventional model performance measures considering more simulation scenarios, and a prediction of binary incident CVD as exemplar.

## Methods

### Method: overall description

There were three paralleled analyses conducted included univariate simulation (i.e., the true model had a single predictor), multivariate simulation (i.e., the true model had two predictors) and a CVD prediction case study. These models were often seen in practice [9, 10]. The pseudo health care data were simulated from the causal true models (assumed to be logistic model). The analysis cohort was extracted from National Health and Nutrition Examination Survey (NHANES) 2017–2018 [11] for case study. This cross-sectional survey represented the general health and nutrition of the general population in US, which was often used for study of risk prediction [12] or association [13]. Cycle of year 2017–2018 was selected since it was the latest year that the data collection was not interrupted by COVID19 [14]. At last, a series of proxy models were then compared to the true model (or pseudo true model for case study) with model performance measures. eFigure I & II described the process.

### Method: model performance measures

Model performance measures were considered from three perspectives: discrimination, calibration, and overall performance. Discrimination measures the ability of models to discriminate high risk patients from low risk, e.g., C statistic [15]. Calibration measures the agreement between observed events rate and predicted risk, e.g., Harrell’s E statistic [16]. Overall performance measures included discrimination, calibration and others, e.g., Brier score [15] measures overall prediction error of model, Pseudo R-squares [15, 16] measure the overall model performance with fitted likelihood. “Global shrinkage factor” [17] measures overfitting of model. Overall, the study considered 25 conventional used measures (eTables 1 & 2).

### Methods: Monte Carlo simulations and model comparisons

Health care data were first simulated using pre-defined underlying casual true model for both simulations. The simulated health care data includes binary outcome variable, causal predictors, and proxy predictors. Causal predictors were first simulated, then the binary outcome variable was simulated using the model formula of the causal true model. Then proxy predictors which were not used to simulate the outcome variable (i.e., non-causal) were simulated. The causal true models and proxy models were then compared in simulated health care data with 25 conventional model performance measures. eFigure III described this process using multivariate simulation as an exemplar.

The study considered the casual true models in various scenarios with multiple parameters to best mimic the practical health care datasets. The univariate simulation considered two simulation scenarios: the single casual predictor in the true model could be either continuous or binary/dichotomous. The multivariate simulation also considered two simulation scenarios: the two causal predictors were both continuous or one continuous and one binary.

Various simulation parameters which would influence the simulated causal predictors, binary outcome variable and proxy predictors were summarised in eTable 3 [9]. E.g., The true effects (i.e., $\:{\beta\:}_{1}$) of the single causal predictor on the outcome variable for univariate simulation was assumed to have range from log(1) to log(4). Correlation to the causal predictor were also considered to best mimic the practice (eTable 4). E.g., the added proxy variable could be independent or correlated (e.g., correlation of 0.3 and 0.5) to the causal predictor in the true model (Appendix II).

For each unique combination of simulation parameter, 100 datasets which contain outcome variable, causal predictors and added proxy predictors with sample size of 15,000 for univariate simulation and sample size of 3, 000 for multivariate simulation were simulated. They were then random split into training set (80%) and testing set (20%) (eFigure II).

Candidate models were considered mimicking real practice, e.g., extra noise was collected (“Add03Model” in eTable 4); causal predictor was replaced by correlated proxy predictors (e.g., “AICAddModel” or “AddCorrVar1” in eTable 4).

The model fitting and testing was similarly between two simulation analysis [4, 5]. Proxy models were trained using simulated training sets and validated in the corresponding testing sets, then the predicted risk was obtained to calculate all the model performance measures with observed outcome. Then the difference of each model performance measure from each proxy model to the true model was calculated (i.e., performance of each proxy model – performance of the true model) [9]. Overall averaged and 95% range of differences of each model performance measure between each type of proxy model and true model across all scenarios were summarised. They were also visualised individually for each model performance measure using boxplot.

## Methods: case study analysis

### Study population and CVD risk factors

The inclusion criteria were patients who participated in NHANES 2017–2018 and aged from 25 to 80. The exclusion criteria were patients did not complete the survey (e.g., their examination sample weights were 0) [2, 18] or those CVD status cannot be determined (33 patients (0.68%)). The primary outcome CVD consisted of coronary heart disease, ischaemic stroke and transient ischaemic attack [2]. This was determined using the combination of the CVD related questionnaire data and ICD-10-CM codes [19] of drug usage [2] (Appendix I). Three sets of predictors were extracted, where the set 1 predictors were used in an external validated and clinical implemented QRISK3 model [2], set 2 predictors may be plausibly related to CVD but not directly in QRISK3 and set 3 predictors (e.g., Creatinine [20]) might have predictive effects to CVD but with more uncertainty (Appendix I).

### Model development, validation, and comparison

The missing values were imputed 10 times with multiple imputation [21] (eTable 6). Two weighted logistic models were trained separately on predictor set1 and predictor set2 to obtain the corresponding linear predictor1 (LP1) and linear predictor2 (LP2). They were used to mimic the causal predictors of the true model in simulation. The Mobile Examination Centre weight was used as the weight variable in modelling since it was the “least common denominator” [18]. Models (eTable 5) were trained on training set (80%) and then predicted on the corresponding testing set (20%); the predicted risk were averaged among 10 missing imputations. Bootstrapping for 1000 times was used to quantify the uncertainty from resampling. Difference of model performance measures between pseudo true model and proxy models was then summarised.

## Methods: statistical software and packages

R was used to simulate and analyse the data [22]. Models were fitted with “glm” [23], and “survey” [24] (weighted logistic model). Model performance measures were calculated with “rms” [25], “PseudoR2” [26] or mathematical formula (eTable 2). Tables and Figures were formatted with “reporter” [27], “ggplot2” [28] and “draw.io” [29].

## Results

There were overall 1,792 combinations of parameters for univariate simulation and 7,280 combinations of parameters for multivariate simulation, which corresponds to 179.2k simulated datasets with sample size of 15,000 for univariate analysis and 728k simulated datasets with sample size of 3,000 for multivariate analysis (eFigure II). The sample size of the case study was 4,850 (Table 1). Overall, 10 types of models were considered in univariate simulation which corresponds to over 1.792 million fitted models, 12 types of models were considered for multivariate analysis which corresponds to over 8.736 million models and 18 types of models were considered in case study which corresponds to over 180 models.

**Table 1:** Baseline characteristics of the case study population (patients aged 25–80 years who participated in NHANES 2017–2018)

	Derivation cohort	Validation cohort
General characteristics		
Number of patients	3880	970
Number of female patients (%)	2008 (51.8)	511 (52.7)
Number of CVD cases (%)	533 (13.7)	163 (16.8)
Considered risk factors		
Age (Mean (SD))	53.6 (16.2)	53.9 (16.5)
Albumin (Mean (SD))	64.1 (430.5)	55.3 (487.2)
BMI (Mean (SD))	30.1 (7.4)	29.7 (7.1)
Cholesterol/HDL ratio (Mean (SD))	3.8 (1.4)	3.7 (1.2)
C-Reactive Protein (Mean (SD))	4.3 (8.4)	4.1 (7.7)
Cotinine (Mean (SD))	0.1 (0.1)	0.1 (0.1)
Creatinine (Mean (SD))	1252.6 (843.0)	1257.9 (840.8)
DBP (Mean (SD))	74.1 (11.7)	73.7 (11.3)
Ratio of family income to poverty (Mean (SD))	2.6 (1.6)	2.6 (1.6)
Ferritin (Mean (SD))	0.2 (0.2)	0.2 (0.2)
Glycohemoglobin (Mean (SD))	5.9 (1.1)	5.9 (1.2)
Median stiffness of kidney (Mean (SD))	6.2 (5.5)	5.9 (4.6)
Mercury (Mean (SD))	0.0002 (0.0008)	0.0002 (0.0008)
PULSE (Mean (SD))	70.5 (11.6)	70.1 (11.5)
AST/ALT ratio (Mean (SD))	1.1 (0.4)	1.1 (0.4)
SBP (Mean (SD))	127.3 (19.6)	128.0 (20.5)
Sleep hours (Mean (SD))	7.6 (1.7)	7.5 (1.7)
Total Vitamin D (Mean (SD))	70.3 (32.6)	69.7 (31.5)
Vitamin A (Mean (SD))	0.5 (0.2)	0.5 (0.2)
Vitamin C (Mean (SD))	9.0 (5.1)	8.8 (5.0)
Vitamin E (Mean (SD))	12.7 (4.5)	12.7 (4.9)
Number of those on hypertension treatment (%)	1498 (38.6)	380 (39.2)
Number of those with diabetes or are taking insulin (%)	683 (17.6)	186 (19.2)
Number of those with close relative had heart attack (%)	518 (13.4)	132 (13.6)
Number of those with kidney disease (%)	530 (13.7)	137 (14.1)
Ethnicity		
Number of Non-Hispanic White (%)	1333 (34.4)	349 (36.0)
Number of Non-Hispanic Black (%)	900 (23.2)	251 (25.9)
Number of Non-Hispanic Asian (%)	580 (14.9)	130 (13.4)
Number of Other Hispanic (%)	350 (9.0)	91 (9.4)
Number of Mexican American (%)	511 (13.2)	111 (11.4)
Number of Other Race - Including Multi-Racial (%)	206 (5.3)	38 (3.9)
Marriage		
Number of Married or Living with partner (%)	2375 (61.2)	594 (61.2)
Number of Never married (%)	556 (14.3)	129 (13.3)
Number of Once married but separated (%)	949 (24.5)	247 (25.5)
Education		
Number of Less than 9th grade (%)	344 (8.9)	101 (10.4)
Number of 9-11th grade and 12th without diploma (%)	450 (11.6)	97 (10.0)
Number of High school graduate/GED or equivalent (%)	893 (23.0)	226 (23.3)
Number of Some college or AA degree (%)	1210 (31.2)	315 (32.5)
Number of College graduate or above (%)	983 (25.3)	231 (23.8)

Table 2 (univariate), Table 3 (multivariate), Table 4 & eTables 7, 8, 9, 10 and 11 (case analysis) summarises mean and 95% range of difference of performance measurements between proxy models and the true/pseudo-true model considering all scenarios together. The true model had overall C statistic and 95% range of 0.67 (0.51, 0.96) across all scenarios in univariate simulation, 0.81 (0.54, 0.98) in multivariate simulation, 0.85 (0.82, 0.88) in univariate case analysis and 0.85 (0.82, 0.88) in multivariate case analysis.

**Table 2:** Comparison of the model performance between the univariate true model and other proxy models in univariate simulation

		Difference of model performance between the other model and the true model* (Mean (95% Range))								
		Flip a fair coin	Add one more variable to the true model				True variable was included before the procedure		True variable was excluded before the procedure	
	TrueModel	FlipCoin	AddIndModel	Add03Model	Add05Model	Add08Model	AICTrueModel	ShrinkTrueModel	AICAddModel	ShrinkAddModel
AUC	0.67 (0.51, 0.96)	-0.17 (-0.46, -0.01)	0.00 (-0.01, 0.01)	-0.00 (-0.01, 0.01)	-0.00 (-0.01, 0.01)	-0.00 (-0.01, 0.01)	-0.00 (-0.01, 0.01)	-0.00 (-0.01, 0.01)	-0.07 (-0.29, 0.01)	-0.07 (-0.29, 0.01)
Dxy	0.34 (0.01, 0.91)	-0.34 (-0.91, -0.01)	0.00 (-0.02, 0.02)	-0.00 (-0.02, 0.02)	-0.00 (-0.02, 0.02)	-0.00 (-0.02, 0.02)	-0.00 (-0.02, 0.02)	-0.00 (-0.02, 0.02)	-0.13 (-0.58, 0.01)	-0.13 (-0.58, 0.01)
gIndex	0.15 (0.00, 0.44)	-0.15 (-0.44, -0.00)	0.00 (-0.00, 0.01)	0.00 (-0.00, 0.01)	0.00 (-0.00, 0.01)	0.00 (-0.00, 0.01)	0.00 (-0.00, 0.01)	-0.01 (-0.08, 0.00)	-0.06 (-0.27, 0.01)	-0.07 (-0.28, -0.00)
U	0.00 (-0.00, 0.00)	0.13 (0.01, 0.23)	0.00 (-0.00, 0.00)	-0.00 (-0.00, 0.00)	0.00 (-0.00, 0.00)	0.00 (-0.00, 0.00)	0.00 (-0.00, 0.00)	0.00 (-0.00, 0.07)	0.00 (-0.00, 0.00)	0.00 (-0.00, 0.01)
D	0.18 (-0.00, 0.83)	-0.18 (-0.83, -0.00)	-0.00 (-0.00, 0.00)	-0.00 (-0.00, 0.00)	-0.00 (-0.00, 0.00)	-0.00 (-0.00, 0.00)	-0.00 (-0.00, 0.00)	-0.00 (-0.01, 0.00)	-0.11 (-0.58, 0.00)	-0.11 (-0.58, 0.00)
Q	0.18 (-0.00, 0.83)	-0.31 (-0.86, -0.11)	-0.00 (-0.00, 0.00)	-0.00 (-0.00, 0.00)	-0.00 (-0.00, 0.00)	-0.00 (-0.00, 0.00)	-0.00 (-0.00, 0.00)	-0.01 (-0.08, 0.00)	-0.11 (-0.58, 0.00)	-0.11 (-0.58, 0.00)
OR_caliLarge	1.00 (0.93, 1.09)	1.13 (0.19, 1.76)	0.00 (-0.00, 0.00)	0.00 (-0.00, 0.00)	0.00 (-0.00, 0.00)	-0.00 (-0.00, 0.00)	0.00 (-0.00, 0.00)	0.00 (-0.00, 0.00)	0.00 (-0.03, 0.03)	-0.00 (-0.03, 0.03)
Emax_ab	0.02 (0.00, 0.05)	0.16 (0.04, 0.23)	0.00 (-0.00, 0.02)	0.00 (-0.00, 0.02)	0.00 (-0.00, 0.02)	0.00 (-0.00, 0.02)	0.01 (-0.01, 0.10)	0.01 (-0.02, 0.13)	0.02 (-0.03, 0.13)	0.00 (-0.04, 0.04)
E_abMean	0.01 (0.00, 0.02)	NA	0.00 (-0.00, 0.01)	0.00 (-0.00, 0.01)	0.00 (-0.00, 0.01)	0.00 (-0.00, 0.01)	0.00 (-0.00, 0.01)	0.01 (-0.01, 0.07)	0.00 (-0.01, 0.02)	0.00 (-0.01, 0.02)
Emax_01	0.08 (0.00, 0.62)	0.10 (-0.39, 0.21)	-0.00 (-0.04, 0.03)	-0.00 (-0.05, 0.04)	-0.00 (-0.04, 0.04)	-0.00 (-0.04, 0.04)	0.01 (-0.14, 0.25)	0.04 (-0.10, 0.63)	0.06 (-0.16, 0.78)	0.08 (-0.22, 0.67)
E_01Mean	0.04 (0.00, 0.28)	NA	-0.00 (-0.02, 0.02)	-0.00 (-0.03, 0.02)	-0.00 (-0.03, 0.02)	-0.00 (-0.03, 0.02)	0.00 (-0.07, 0.11)	0.02 (-0.03, 0.26)	0.03 (-0.09, 0.27)	0.04 (-0.04, 0.28)
Eavg_LOWESS	0.01 (0.00, 0.02)	0.16 (0.04, 0.23)	0.00 (-0.00, 0.01)	0.00 (-0.00, 0.01)	0.00 (-0.00, 0.01)	0.00 (-0.00, 0.01)	0.00 (-0.00, 0.01)	0.01 (-0.00, 0.06)	0.00 (-0.01, 0.02)	0.00 (-0.01, 0.02)
ECI_LOWESS	0.02 (0.00, 0.07)	3.27 (0.20, 5.59)	0.01 (-0.00, 0.04)	0.01 (-0.00, 0.04)	0.01 (-0.00, 0.04)	0.01 (-0.00, 0.04)	0.01 (-0.01, 0.08)	0.05 (-0.01, 0.55)	0.02 (-0.03, 0.09)	0.02 (-0.03, 0.13)
brierScore	0.18 (0.08, 0.22)	0.07 (0.03, 0.17)	0.00 (-0.00, 0.00)	0.00 (-0.00, 0.00)	0.00 (-0.00, 0.00)	0.00 (-0.00, 0.00)	0.00 (-0.00, 0.00)	0.00 (-0.00, 0.01)	0.02 (-0.00, 0.11)	0.02 (-0.00, 0.11)
R2_Nagelkerke	0.19 (0.00, 0.76)	-0.19 (-0.76, -0.00)	-0.00 (-0.00, 0.00)	-0.00 (-0.00, 0.00)	-0.00 (-0.00, 0.00)	-0.00 (-0.00, 0.00)	-0.00 (-0.00, 0.00)	-0.00 (-0.01, 0.00)	-0.11 (-0.50, 0.00)	-0.11 (-0.50, 0.00)
R2_McFadden	0.14 (0.00, 0.61)	-0.14 (-0.61, -0.00)	-0.00 (-0.00, 0.00)	-0.00 (-0.00, 0.00)	-0.00 (-0.00, 0.00)	-0.00 (-0.00, 0.00)	-0.00 (-0.00, 0.00)	-0.00 (-0.01, 0.00)	-0.08 (-0.43, 0.00)	-0.08 (-0.43, 0.00)
R2_McFaddenAdj	0.14 (-0.00, 0.61)	-0.14 (-0.61, 0.00)	-0.00 (-0.00, -0.00)	-0.00 (-0.00, -0.00)	-0.00 (-0.00, -0.00)	-0.00 (-0.00, -0.00)	-0.00 (-0.01, 0.00)	-0.00 (-0.01, -0.00)	-0.09 (-0.43, -0.00)	-0.09 (-0.43, -0.00)
R2_CoxSnell	0.14 (0.00, 0.56)	-0.14 (-0.56, -0.00)	-0.00 (-0.00, 0.00)	-0.00 (-0.00, 0.00)	-0.00 (-0.00, 0.00)	-0.00 (-0.00, 0.00)	-0.00 (-0.00, 0.00)	-0.00 (-0.00, 0.00)	-0.08 (-0.37, 0.00)	-0.08 (-0.37, 0.00)
R2_AldrichNelson	0.12 (0.00, 0.45)	-0.12 (-0.45, -0.00)	-0.00 (-0.00, 0.00)	-0.00 (-0.00, 0.00)	-0.00 (-0.00, 0.00)	-0.00 (-0.00, 0.00)	-0.00 (-0.00, 0.00)	-0.00 (-0.00, 0.00)	-0.07 (-0.29, 0.00)	-0.07 (-0.29, 0.00)
R2_VeallZimmermann	0.21 (0.00, 0.79)	-0.21 (-0.79, -0.00)	-0.00 (-0.00, 0.00)	-0.00 (-0.00, 0.00)	-0.00 (-0.00, 0.00)	-0.00 (-0.00, 0.00)	-0.00 (-0.01, 0.00)	-0.00 (-0.01, 0.00)	-0.12 (-0.50, 0.00)	-0.12 (-0.50, 0.00)
R2_Efron	0.16 (-0.00, 0.66)	-0.31 (-0.69, -0.12)	-0.00 (-0.00, 0.00)	-0.00 (-0.00, 0.00)	-0.00 (-0.00, 0.00)	-0.00 (-0.00, 0.00)	-0.00 (-0.00, 0.00)	-0.00 (-0.04, 0.00)	-0.10 (-0.46, 0.00)	-0.10 (-0.46, 0.00)
R2_McKelveyZavoina	0.21 (0.00, 0.86)	-0.21 (-0.86, -0.00)	0.00 (-0.00, 0.00)	0.00 (-0.00, 0.00)	0.00 (-0.00, 0.00)	0.00 (-0.00, 0.00)	0.00 (-0.00, 0.02)	-0.02 (-0.27, -0.00)	-0.11 (-0.47, 0.00)	-0.12 (-0.52, -0.00)
R2_Tjur	0.16 (0.00, 0.66)	-0.16 (-0.66, -0.00)	0.00 (-0.00, 0.00)	0.00 (-0.00, 0.00)	-0.00 (-0.00, 0.00)	-0.00 (-0.00, 0.00)	0.00 (-0.00, 0.00)	-0.01 (-0.13, 0.00)	-0.09 (-0.46, 0.00)	-0.10 (-0.47, -0.00)
GlobalShrinkageFactor	0.86 (-0.26, 1.00)	NA	-0.12 (-0.79, -0.00)	-0.13 (-0.91, -0.00)	-0.13 (-0.82, -0.00)	-0.13 (-0.87, -0.00)	-0.44 (-4.36, -0.00)	-0.18 (-1.36, -0.00)	-0.89 (-8.08, -0.00)	-0.54 (-5.55, -0.00)
IDI_diff1	0.16 (0.00, 0.66)	-0.16 (-0.66, -0.00)	0.00 (-0.00, 0.00)	0.00 (-0.00, 0.00)	-0.00 (-0.00, 0.00)	-0.00 (-0.00, 0.00)	0.00 (-0.00, 0.00)	-0.01 (-0.13, 0.00)	-0.09 (-0.46, 0.00)	-0.10 (-0.47, -0.00)

* The difference of performance measure = performance measure of each model - performance measure of the true model

**Table 3:** Comparison of the model performance between the multivariate true model and other proxy models in multivariate simulation

		Difference of model performance between the other model and the true model* (Mean (95% Range))										
								Missing variable1				
		Flip a fair coin	Univariate model		Add one more variable			Add one more continuous variable			Add one more categorical variable	
	TrueModel	FlipCoin	Variable1	Variable2	AddIndCont	AddCorrCont	AddCat	AddIndVar1	AddCorrVar1	AddCorrVar2	AddIndCorrVar1	AddCorrVar2
AUC	0.81 (0.54, 0.98)	-0.31 (-0.48, -0.04)	-0.04 (-0.21, 0.00)	-0.14 (-0.41, 0.00)	-0.00 (-0.00, 0.00)	-0.00 (-0.00, 0.00)	-0.00 (-0.00, 0.00)	-0.14 (-0.41, 0.00)	-0.08 (-0.28, 0.00)	-0.13 (-0.40, 0.00)	-0.12 (-0.37, 0.00)	-0.13 (-0.40, 0.00)
Dxy	0.62 (0.09, 0.96)	-0.62 (-0.96, -0.09)	-0.09 (-0.42, 0.00)	-0.27 (-0.82, 0.00)	-0.00 (-0.00, 0.00)	-0.00 (-0.00, 0.00)	-0.00 (-0.00, 0.00)	-0.27 (-0.82, 0.00)	-0.16 (-0.57, 0.00)	-0.26 (-0.80, 0.00)	-0.23 (-0.73, 0.00)	-0.27 (-0.81, 0.00)
gIndex	0.29 (0.04, 0.48)	-0.29 (-0.48, -0.04)	-0.04 (-0.20, -0.00)	-0.13 (-0.40, -0.00)	0.00 (-0.00, 0.00)	0.00 (-0.00, 0.00)	0.00 (-0.00, 0.00)	-0.13 (-0.40, 0.00)	-0.07 (-0.27, 0.00)	-0.12 (-0.39, 0.00)	-0.11 (-0.35, 0.00)	-0.13 (-0.39, 0.00)
U	0.00 (-0.00, 0.00)	0.09 (0.01, 0.22)	0.00 (-0.00, 0.00)	0.00 (-0.00, 0.00)	0.00 (-0.00, 0.00)	0.00 (-0.00, 0.00)	0.00 (-0.00, 0.00)	0.00 (-0.00, 0.00)	0.00 (-0.00, 0.00)	0.00 (-0.00, 0.00)	0.00 (-0.00, 0.00)	0.00 (-0.00, 0.00)
D	0.42 (0.01, 1.02)	-0.42 (-1.02, -0.01)	-0.10 (-0.57, 0.00)	-0.24 (-0.79, 0.00)	-0.00 (-0.00, 0.00)	-0.00 (-0.00, 0.00)	-0.00 (-0.00, 0.00)	-0.24 (-0.79, 0.00)	-0.18 (-0.67, 0.00)	-0.24 (-0.78, 0.00)	-0.22 (-0.74, 0.00)	-0.24 (-0.79, 0.00)
Q	0.42 (0.01, 1.02)	-0.51 (-1.04, -0.14)	-0.10 (-0.57, 0.00)	-0.24 (-0.79, 0.00)	-0.00 (-0.00, 0.00)	-0.00 (-0.00, 0.00)	-0.00 (-0.00, 0.00)	-0.24 (-0.79, 0.00)	-0.18 (-0.67, 0.00)	-0.24 (-0.78, 0.00)	-0.22 (-0.74, 0.00)	-0.24 (-0.79, 0.00)
OR_caliLarge	1.00 (0.94, 1.07)	0.82 (0.17, 1.66)	0.00 (-0.02, 0.04)	-0.00 (-0.05, 0.03)	-0.00 (-0.00, 0.00)	0.00 (-0.00, 0.00)	0.00 (-0.00, 0.00)	-0.00 (-0.05, 0.03)	-0.00 (-0.04, 0.03)	-0.00 (-0.05, 0.03)	-0.00 (-0.04, 0.03)	-0.00 (-0.05, 0.03)
Emax_ab	0.02 (0.00, 0.05)	0.12 (0.02, 0.21)	0.00 (-0.02, 0.03)	-0.00 (-0.04, 0.03)	0.00 (-0.00, 0.00)	0.00 (-0.00, 0.00)	0.00 (-0.00, 0.00)	-0.00 (-0.03, 0.03)	0.00 (-0.02, 0.03)	0.00 (-0.03, 0.04)	-0.00 (-0.03, 0.03)	-0.00 (-0.03, 0.03)
E_abMean	0.01 (0.00, 0.02)	NA	0.00 (-0.01, 0.01)	-0.00 (-0.01, 0.01)	0.00 (-0.00, 0.00)	0.00 (-0.00, 0.00)	0.00 (-0.00, 0.00)	0.00 (-0.01, 0.01)	0.00 (-0.01, 0.01)	0.00 (-0.01, 0.01)	-0.00 (-0.01, 0.01)	-0.00 (-0.01, 0.01)
Emax_01	0.02 (0.00, 0.10)	0.11 (0.01, 0.21)	0.03 (-0.02, 0.38)	0.04 (-0.02, 0.37)	0.00 (-0.00, 0.00)	0.00 (-0.00, 0.00)	0.00 (-0.00, 0.00)	0.04 (-0.02, 0.36)	0.01 (-0.02, 0.07)	0.02 (-0.03, 0.21)	0.02 (-0.03, 0.14)	0.03 (-0.03, 0.25)
E_01Mean	0.01 (0.00, 0.05)	NA	0.01 (-0.01, 0.18)	0.02 (-0.01, 0.18)	0.00 (-0.00, 0.00)	0.00 (-0.00, 0.00)	0.00 (-0.00, 0.00)	0.02 (-0.01, 0.17)	0.00 (-0.01, 0.03)	0.01 (-0.01, 0.11)	0.01 (-0.01, 0.07)	0.01 (-0.01, 0.13)
Eavg_LOWESS	0.01 (0.00, 0.02)	0.13 (0.03, 0.22)	0.00 (-0.01, 0.01)	0.00 (-0.01, 0.01)	0.00 (-0.00, 0.00)	-0.00 (-0.00, 0.00)	0.00 (-0.00, 0.00)	0.00 (-0.01, 0.01)	0.00 (-0.01, 0.01)	0.00 (-0.01, 0.01)	0.00 (-0.01, 0.01)	0.00 (-0.01, 0.01)
ECI_LOWESS	0.02 (0.00, 0.06)	2.16 (0.14, 5.20)	0.00 (-0.02, 0.05)	0.00 (-0.03, 0.04)	0.00 (-0.00, 0.00)	-0.00 (-0.00, 0.00)	0.00 (-0.00, 0.00)	0.01 (-0.02, 0.06)	0.01 (-0.02, 0.04)	0.01 (-0.02, 0.06)	0.01 (-0.02, 0.05)	0.01 (-0.02, 0.06)
brierScore	0.14 (0.05, 0.22)	0.11 (0.03, 0.20)	0.02 (-0.00, 0.10)	0.05 (-0.00, 0.15)	0.00 (-0.00, 0.00)	0.00 (-0.00, 0.00)	0.00 (-0.00, 0.00)	0.05 (-0.00, 0.15)	0.03 (-0.00, 0.13)	0.05 (-0.00, 0.15)	0.04 (-0.00, 0.14)	0.05 (-0.00, 0.15)
R2_Nagelkerke	0.43 (0.01, 0.86)	-0.43 (-0.86, -0.01)	-0.08 (-0.43, 0.00)	-0.23 (-0.72, 0.00)	-0.00 (-0.00, 0.00)	-0.00 (-0.00, 0.00)	-0.00 (-0.00, 0.00)	-0.23 (-0.72, 0.00)	-0.16 (-0.58, 0.00)	-0.23 (-0.71, 0.00)	-0.20 (-0.66, 0.00)	-0.23 (-0.72, 0.00)
R2_McFadden	0.32 (0.00, 0.75)	-0.32 (-0.75, -0.00)	-0.07 (-0.42, 0.00)	-0.18 (-0.58, 0.00)	-0.00 (-0.00, 0.00)	-0.00 (-0.00, 0.00)	-0.00 (-0.00, 0.00)	-0.18 (-0.58, 0.00)	-0.14 (-0.49, 0.00)	-0.18 (-0.57, 0.00)	-0.17 (-0.54, 0.00)	-0.18 (-0.58, 0.00)
R2_McFaddenAdj	0.32 (0.00, 0.74)	-0.32 (-0.74, -0.00)	-0.07 (-0.42, 0.00)	-0.18 (-0.58, 0.00)	-0.00 (-0.00, -0.00)	-0.00 (-0.00, -0.00)	-0.00 (-0.00, -0.00)	-0.18 (-0.58, 0.00)	-0.14 (-0.49, 0.00)	-0.18 (-0.57, 0.00)	-0.17 (-0.54, 0.00)	-0.18 (-0.58, 0.00)
R2_CoxSnell	0.31 (0.01, 0.64)	-0.31 (-0.64, -0.01)	-0.06 (-0.32, 0.00)	-0.17 (-0.53, 0.00)	-0.00 (-0.00, 0.00)	-0.00 (-0.00, 0.00)	-0.00 (-0.00, 0.00)	-0.17 (-0.53, 0.00)	-0.12 (-0.43, 0.00)	-0.17 (-0.53, 0.00)	-0.15 (-0.49, 0.00)	-0.17 (-0.53, 0.00)
R2_AldrichNelson	0.26 (0.01, 0.51)	-0.26 (-0.51, -0.01)	-0.05 (-0.25, 0.00)	-0.14 (-0.43, 0.00)	-0.00 (-0.00, 0.00)	-0.00 (-0.00, 0.00)	-0.00 (-0.00, 0.00)	-0.14 (-0.43, 0.00)	-0.09 (-0.34, 0.00)	-0.13 (-0.43, 0.00)	-0.12 (-0.39, 0.00)	-0.14 (-0.43, 0.00)
R2_VeallZimmermann	0.46 (0.01, 0.87)	-0.46 (-0.87, -0.01)	-0.09 (-0.43, 0.00)	-0.24 (-0.74, 0.00)	-0.00 (-0.00, 0.00)	-0.00 (-0.00, 0.00)	-0.00 (-0.00, 0.00)	-0.24 (-0.74, 0.00)	-0.16 (-0.59, 0.00)	-0.24 (-0.74, 0.00)	-0.21 (-0.68, 0.00)	-0.24 (-0.74, 0.00)
R2_Efron	0.35 (0.01, 0.78)	-0.45 (-0.79, -0.14)	-0.08 (-0.42, 0.00)	-0.20 (-0.62, 0.00)	-0.00 (-0.00, 0.00)	-0.00 (-0.00, 0.00)	-0.00 (-0.00, 0.00)	-0.20 (-0.62, 0.00)	-0.14 (-0.52, 0.00)	-0.20 (-0.62, 0.00)	-0.18 (-0.58, 0.00)	-0.20 (-0.62, 0.00)
R2_McKelveyZavoina	0.47 (0.01, 0.94)	-0.47 (-0.94, -0.01)	-0.09 (-0.49, 0.00)	-0.26 (-0.81, -0.00)	0.00 (-0.00, 0.00)	0.00 (-0.00, 0.00)	0.00 (-0.00, 0.00)	-0.26 (-0.81, 0.00)	-0.19 (-0.67, 0.00)	-0.26 (-0.81, 0.00)	-0.23 (-0.75, 0.00)	-0.26 (-0.81, 0.00)
R2_Tjur	0.35 (0.01, 0.78)	-0.35 (-0.78, -0.01)	-0.08 (-0.41, -0.00)	-0.20 (-0.62, 0.00)	-0.00 (-0.00, 0.00)	0.00 (-0.00, 0.00)	0.00 (-0.00, 0.00)	-0.20 (-0.62, 0.00)	-0.14 (-0.52, 0.00)	-0.20 (-0.62, 0.00)	-0.18 (-0.58, 0.00)	-0.20 (-0.62, 0.00)
GlobalShrinkageFactor	0.99 (0.91, 1.00)	NA	-0.04 (-0.38, 0.00)	-0.05 (-0.42, 0.00)	-0.01 (-0.05, -0.00)	-0.01 (-0.05, -0.00)	-0.01 (-0.05, -0.00)	-0.05 (-0.40, 0.00)	-0.01 (-0.11, 0.00)	-0.04 (-0.37, 0.00)	-0.03 (-0.25, 0.00)	-0.05 (-0.40, 0.00)
IDI_diff1	0.35 (0.01, 0.78)	-0.35 (-0.78, -0.01)	-0.08 (-0.41, -0.00)	-0.20 (-0.62, 0.00)	-0.00 (-0.00, 0.00)	0.00 (-0.00, 0.00)	0.00 (-0.00, 0.00)	-0.20 (-0.62, 0.00)	-0.14 (-0.52, 0.00)	-0.20 (-0.62, 0.00)	-0.18 (-0.58, 0.00)	-0.20 (-0.62, 0.00)

*The difference of performance measure = performance measure of each model - performance measure of the true model

**Table 4:** Comparison of the model performance between the pseudo true model and other proxy models in NHANES 2017–2018

		Difference of model performance* between the other model and the pseudo true model** (Mean (95% Range))						
		Flip a fair coin	Add one more variable while considering LP1		LP1 was included before the procedure		LP1 was excluded before the procedure	
	PseudoTrueModel	FlipCoin	AddLowCorrModel	Add03Model	AICTrueModel	ShrinkTrueModel	AICAddModel	ShrinkAddModel
AUC	0.85 (0.82, 0.88)	-0.35 (-0.38, -0.32)	0.00 (0.00, 0.00)	0.00 (-0.00, 0.00)	0.01 (-0.00, 0.02)	0.01 (-0.00, 0.02)	-0.07 (-0.10, -0.04)	-0.06 (-0.09, -0.03)
Dxy	0.70 (0.64, 0.76)	-0.70 (-0.76, -0.64)	0.00 (0.00, 0.01)	0.00 (-0.00, 0.00)	0.01 (-0.00, 0.03)	0.01 (-0.00, 0.03)	-0.14 (-0.21, -0.07)	-0.12 (-0.18, -0.05)
gIndex	0.17 (0.16, 0.18)	-0.17 (-0.18, -0.16)	0.00 (0.00, 0.00)	-0.00 (-0.00, -0.00)	0.01 (0.01, 0.02)	-0.01 (-0.01, -0.01)	-0.06 (-0.07, -0.04)	-0.07 (-0.08, -0.06)
U	0.01 (-0.00, 0.02)	0.47 (0.38, 0.56)	-0.00 (-0.00, 0.00)	0.00 (0.00, 0.00)	-0.00 (-0.01, 0.00)	0.00 (-0.00, 0.01)	0.01 (-0.00, 0.02)	0.02 (0.00, 0.03)
D	0.24 (0.19, 0.30)	-0.24 (-0.30, -0.19)	0.00 (0.00, 0.00)	0.00 (-0.00, 0.00)	0.01 (-0.01, 0.03)	0.01 (-0.00, 0.03)	-0.11 (-0.16, -0.06)	-0.11 (-0.16, -0.06)
Q	0.23 (0.18, 0.28)	-0.71 (-0.79, -0.63)	0.00 (0.00, 0.00)	-0.00 (-0.00, 0.00)	0.02 (-0.00, 0.04)	0.01 (-0.00, 0.03)	-0.12 (-0.17, -0.07)	-0.12 (-0.17, -0.07)
OR_caliLarge	0.81 (0.70, 0.93)	4.16 (3.45, 4.97)	0.00 (0.00, 0.01)	-0.00 (-0.00, -0.00)	0.05 (0.03, 0.08)	0.00 (-0.02, 0.02)	-0.07 (-0.13, -0.00)	-0.12 (-0.18, -0.06)
Emax_ab	0.07 (0.02, 0.14)	0.26 (0.18, 0.32)	-0.00 (-0.00, 0.00)	0.00 (-0.00, 0.00)	-0.02 (-0.05, 0.02)	0.04 (-0.00, 0.08)	0.03 (-0.04, 0.12)	0.11 (0.01, 0.21)
E_abMean	0.03 (0.01, 0.05)	NA	-0.00 (-0.00, -0.00)	0.00 (0.00, 0.00)	-0.01 (-0.01, -0.00)	0.00 (-0.00, 0.01)	0.01 (0.00, 0.02)	0.02 (0.01, 0.03)
Emax_01	0.07 (0.02, 0.14)	0.26 (0.18, 0.32)	-0.00 (-0.00, 0.00)	0.00 (-0.00, 0.00)	-0.02 (-0.05, 0.02)	0.04 (-0.00, 0.08)	0.03 (-0.04, 0.12)	0.11 (0.01, 0.22)
E_01Mean	0.04 (0.01, 0.09)	NA	0.00 (-0.00, 0.00)	0.00 (-0.00, 0.00)	-0.01 (-0.03, 0.01)	0.02 (-0.00, 0.04)	0.02 (-0.03, 0.07)	0.07 (0.01, 0.13)
Eavg_LOWESS	0.03 (0.01, 0.05)	0.30 (0.26, 0.34)	-0.00 (-0.00, 0.00)	0.00 (-0.00, 0.00)	-0.01 (-0.01, 0.00)	0.00 (-0.01, 0.01)	0.02 (0.01, 0.04)	0.03 (0.02, 0.05)
ECI_LOWESS	0.19 (0.04, 0.45)	10.83 (8.99, 12.52)	-0.01 (-0.01, 0.00)	0.00 (-0.00, 0.00)	-0.07 (-0.16, 0.01)	0.05 (-0.06, 0.18)	0.32 (0.08, 0.62)	0.60 (0.27, 1.02)
brierScore	0.11 (0.09, 0.12)	0.14 (0.13, 0.16)	-0.00 (-0.00, -0.00)	0.00 (-0.00, 0.00)	-0.00 (-0.01, 0.00)	-0.00 (-0.00, 0.00)	0.02 (0.01, 0.03)	0.02 (0.01, 0.03)
R2_Nagelkerke	0.36 (0.29, 0.43)	-0.36 (-0.43, -0.29)	0.00 (0.00, 0.01)	0.00 (-0.00, 0.00)	0.02 (-0.01, 0.04)	0.01 (-0.00, 0.04)	-0.16 (-0.23, -0.09)	-0.15 (-0.21, -0.08)
R2_McFadden	0.27 (0.21, 0.32)	-0.27 (-0.32, -0.21)	0.00 (0.00, 0.00)	0.00 (-0.00, 0.00)	0.01 (-0.01, 0.03)	0.01 (-0.00, 0.03)	-0.13 (-0.18, -0.07)	-0.12 (-0.17, -0.06)
R2_McFaddenAdj	0.26 (0.21, 0.32)	-0.26 (-0.32, -0.21)	0.00 (-0.00, 0.00)	-0.00 (-0.00, -0.00)	-0.03 (-0.05, -0.01)	-0.05 (-0.06, -0.03)	-0.17 (-0.23, -0.12)	-0.18 (-0.23, -0.12)
R2_CoxSnell	0.21 (0.17, 0.26)	-0.21 (-0.26, -0.17)	0.00 (0.00, 0.00)	0.00 (-0.00, 0.00)	0.01 (-0.01, 0.02)	0.01 (-0.00, 0.02)	-0.10 (-0.13, -0.05)	-0.09 (-0.13, -0.05)
R2_AldrichNelson	0.19 (0.16, 0.23)	-0.19 (-0.23, -0.16)	0.00 (0.00, 0.00)	0.00 (-0.00, 0.00)	0.01 (-0.00, 0.02)	0.01 (-0.00, 0.02)	-0.08 (-0.11, -0.05)	-0.08 (-0.11, -0.04)
R2_VeallZimmermann	0.41 (0.34, 0.48)	-0.41 (-0.48, -0.34)	0.00 (0.00, 0.01)	0.00 (-0.00, 0.00)	0.02 (-0.01, 0.04)	0.01 (-0.00, 0.04)	-0.17 (-0.24, -0.10)	-0.16 (-0.23, -0.09)
R2_Efron	0.24 (0.18, 0.29)	-1.03 (-1.25, -0.84)	0.00 (0.00, 0.01)	-0.00 (-0.00, 0.00)	0.02 (-0.00, 0.05)	0.01 (-0.01, 0.03)	-0.13 (-0.19, -0.07)	-0.14 (-0.19, -0.08)
R2_McKelveyZavoina	0.46 (0.44, 0.47)	-0.46 (-0.47, -0.44)	0.00 (0.00, 0.00)	-0.00 (-0.00, 0.00)	0.03 (0.02, 0.03)	-0.06 (-0.07, -0.05)	-0.24 (-0.26, -0.21)	-0.28 (-0.30, -0.25)
R2_Tjur	0.23 (0.19, 0.26)	-0.23 (-0.26, -0.19)	0.00 (0.00, 0.00)	-0.00 (-0.00, 0.00)	0.03 (0.02, 0.04)	-0.01 (-0.02, 0.00)	-0.11 (-0.14, -0.08)	-0.13 (-0.16, -0.10)
GlobalShrinkageFactor	0.99 (0.99, 0.99)	NA	-0.00 (-0.01, -0.00)	-0.00 (-0.01, -0.00)	-0.08 (-0.10, -0.07)	-0.11 (-0.14, -0.09)	-0.18 (-0.23, -0.13)	-0.19 (-0.24, -0.14)
IDI_diff1	0.23 (0.19, 0.26)	-0.23 (-0.26, -0.19)	0.00 (0.00, 0.00)	-0.00 (-0.00, 0.00)	0.03 (0.02, 0.04)	-0.01 (-0.02, 0.00)	-0.11 (-0.14, -0.08)	-0.13 (-0.16, -0.10)

*The difference of performance measure = performance measure of each model - performance measure of the pseudo true model

**Pseudo true model was assumed as a model considers linear predictor of causal predictor set 1 (LP1)

Clear differences between the true model and the flip-coin model were shown by all performance measures across all scenarios (e.g., the differences of C-statistic were − 0.17 (-0.46, -0.01) in univariate simulation of Table 2). However, little or even none differences between the true model and proxy models with extra noises were shown by these measures across all scenarios (e.g., the differences of C-statistic were 0.00 (-0.01, 0.01) for “AddIndModel” in Table 2). Differences of these measures between proxy models missing causal predictor and the true model was noticeably smaller than the differences between the true model and the flip-coin model (e.g., difference in C statistic was − 0.07 (-0.29, 0.01) for AICAddModel but − 0.17 (-0.46, -0.01) for FlipCoin in Table 2). Such differences could even be too small in certain cases such that proxy models which miss causal predictors could be considered as “similar” performed model to the true/pseudo true model in practice (e.g., difference in C statistic was − 0.07 (-0.29, 0.01) for “AICAddModel” and “ShrinkAddModel” in Table 2). Overall, this shows that the true model was not necessarily being identified as the “outperformed” model by these measures.

The “PseudoTrueModel” with single LP1 in Table 4 for case study is an instance of the univariate true model in Table 2 in real-world. Similarly, the “PseudoTrueModel” with LP1 & LP2 in eTable 8 is an instance of the multivariate true model in Table 3. Clear differences between the pseudo-true model and the flip-coin model were verified (e.g., difference in C statistic was − 0.35 (-0.38, -0.32) for the flip-coin model in Table 4 & eTable8). Little or even none differences between the pseudo-true model and proxy models with extra noises was verified (e.g., difference in C statistic was 0.00 (-0.00, 0.00) for “Add03Model” in Table 4 & 0.00 (-0.00, 0.01) for “AICFullModel” in eTable 8). The differences of these measures to the true model for the proxy models missing causal predictor was noticeably smaller than the differences for the flip-coin model was also verified (e.g., difference in C statistic was − 0.07 (-0.10, -0.04) for “AICAddModel” in Table 4 & -0.12 (-0.16, -0.08) for “AddLowCorr” only considering LP2 in eTable 8). Overall, it results as a series similarly performed models (e.g., “PseudoTrueModel”, “Univariate model 1” & “AICFullModel” in eTable 8). Additional comparisons assuming the interaction effects of age and sex in the true model results similarly in eTables 10 and 11.

**Fig. 1:**
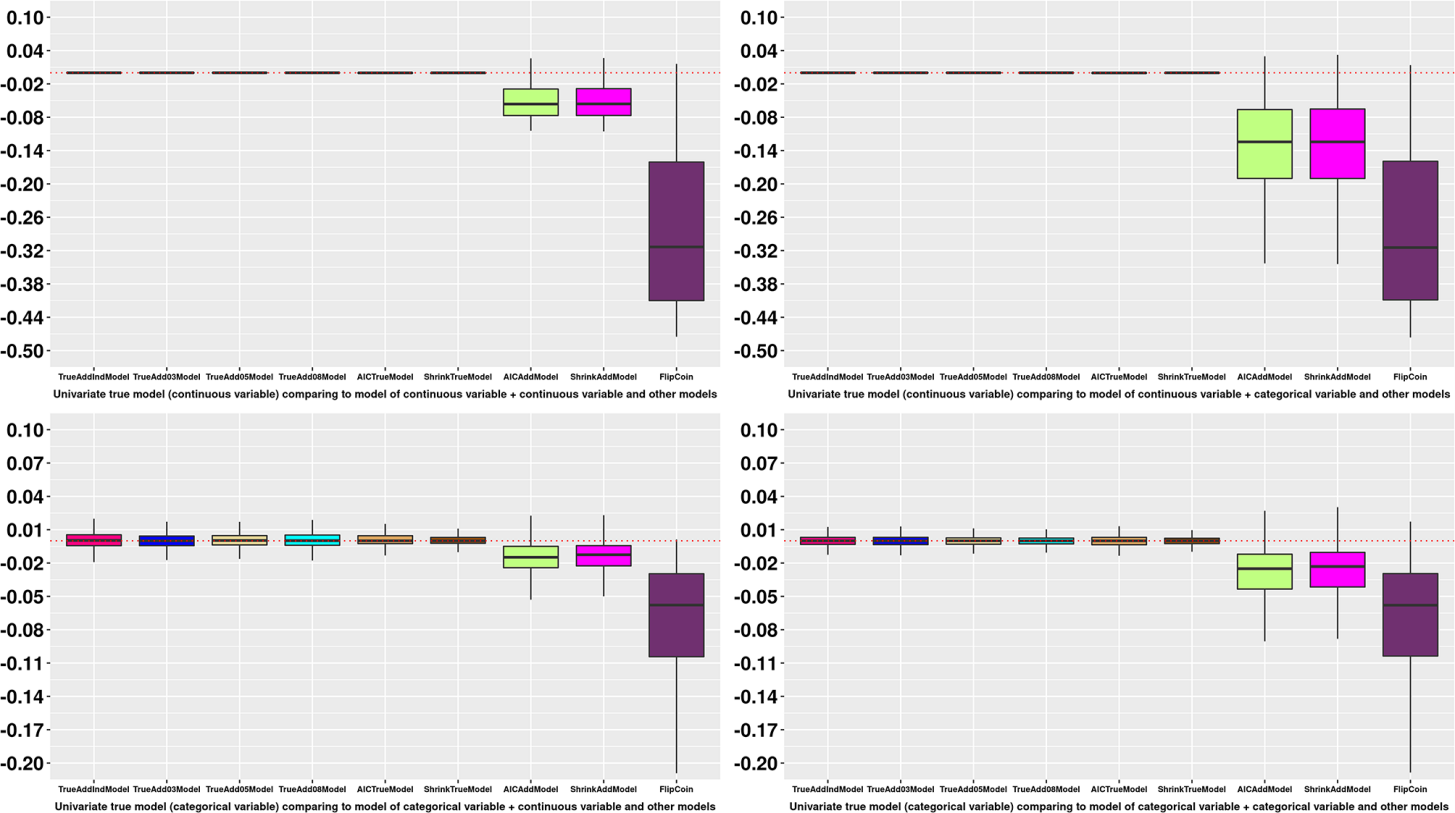
Boxplot of differences of C statistics from candidate models to the true model in univariate simulations. X axis: type of models. Y axis: Differences of C statistics from candidate models to the true model

**Fig. 2:**
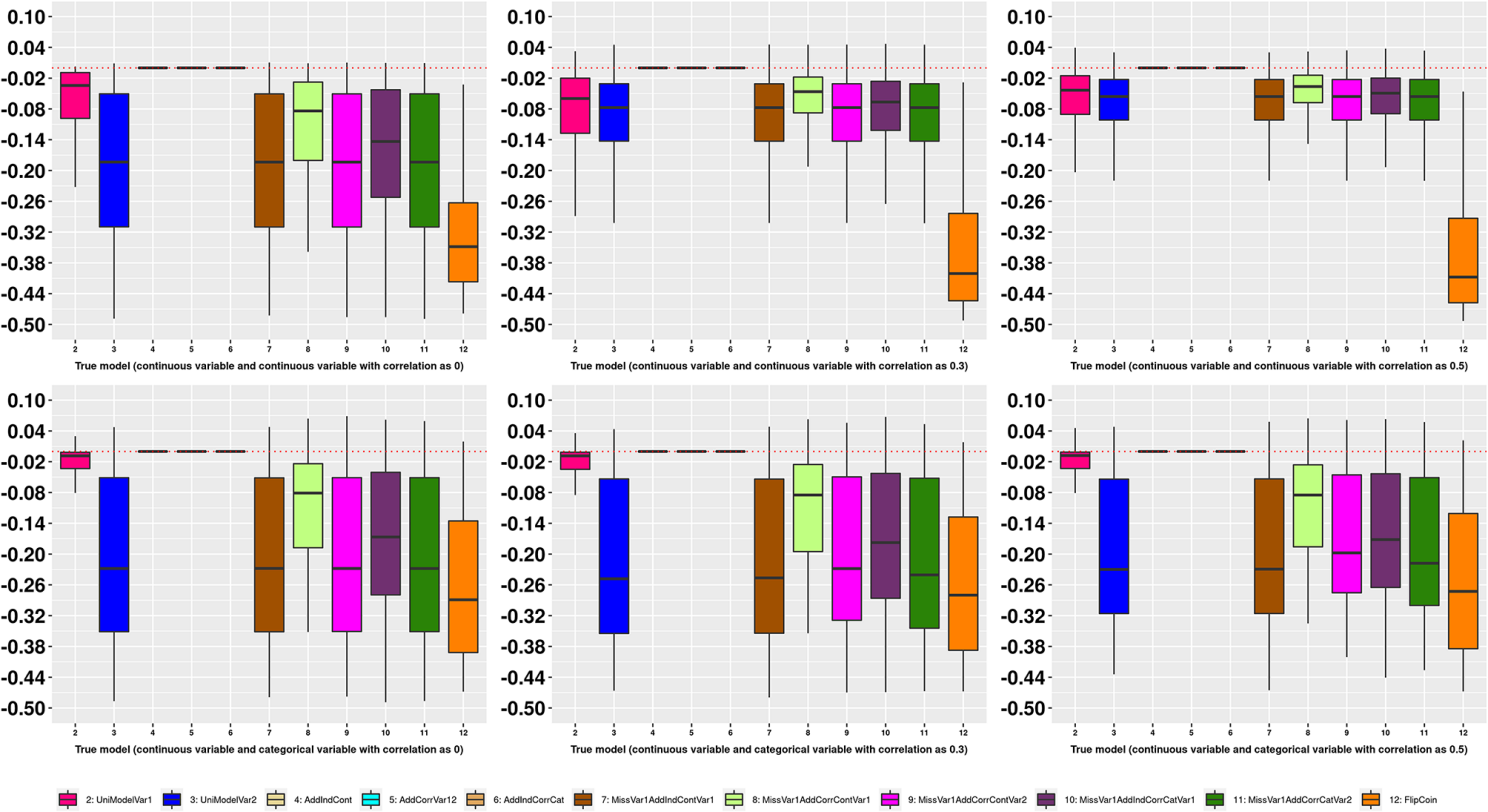
Boxplot of differences of C statistics from candidate models to the true model in multivariate simulations. X axis: type of models. Y axis: Differences of C statistics from candidate models to the true model

Figure 1, eFigure 1.2–1.25 (univariate analysis) and Fig. 2, eFigure 2.2–2.25 (multivariate analysis) visualises the differences of each performance measure between the proxy models and the true model by different scenarios of true model. They show similarly as above such as proxy models could be identified as similar model to the true model (e.g., “AICAddModel” and “ShrinkAddModel” in Fig. 1 when the true model has only single categorical variable).

## Discussion

This study found that the true model is not always identified as the “outperformed” model by current conventional measures for binary outcome, even though such true model is presented in the clinical data. The other “similar” performed model could be proxy models with extra noises or even missing causal predictors. This suggests using these measures for model comparison in binary outcome to obtain individual risk prediction for clinical decision making, would likely end up with a series of “similar” performed models, while the true model may or may not be one of them.

To tackle the issue of inconsistent individual risk prediction among models with similar model performance [6], previous studies have shown that it might not be due to the data quality [8], the issue still persists after switching to new type of models including machine learning [3], a new measure ranking was proposed to gain more insight on individual level risk prediction [7]. This study switches focus from the individual risk prediction to the robustness of model performance measures. This is because the inconsistency issue is essentially about which model to be trusted/applied in the clinical practice for decision making. Ideally, the model which describes the true causal relationship between the outcome variable (e.g., CVD) and causal predictors (i.e., the true model) is preferred. If such true model could be identified, then the issue of inconsistent individual risk prediction could be resolved by reducing similar performed models into this one causal/true model. However, this study has shown the true model is not always identified as the “outperformed” model by current measures for binary outcome in clinical practice.

Using simulation [30] or analytically-derived approaches [31] to evaluate the variability of performance measures was proposed before. The observed decrease of performance measures from the multivariate true model to the univariate proxy model (Table 3) were consistent with Peter et al. [9]. This study adds these measures may identify proxy models missing causal predictors (e.g., with proxy predictors and statistical procedure) as similar model to the true model. Previous research [32] achieved a C-statistic of 0.89 for coronary artery disease using NHANES data; this study achieves 0.85 (0.82, 0.88) for broader CVD outcomes.

The study suggests that treating predictors (i.e., Xs) as fixed values and “unimportant” comparing to outcome variable (i.e., Y) in current consent of statistical modelling is a costly assumption. Though this assumption enables any proxy model with acceptable performance could be fitted to predict outcome variable Y, it leads to swamp the potential true model with these proxy models. This then leads to the issue of models with comparable performance but inconsistent risk prediction for individual patients. Thus, the development and model selection of CPM should consider whether causal predictors were used rather than solely relying on these performance measures. An ideal clinical useful CPM should allow tracing the predicted individual risk back to contributing causal risk factors, thus supporting clinical decision making aligned with the clinical judgement.

A general consent statistical approach to identify the true model from other proxy models is currently missing. Though there were claims that “proxy”/candidate model might be just enough to solve the problem [33, 34] (say risk prediction), previous studies have shown that arbitrarily applying “proxy” models resulting inconsistent individual risk predictions [3, 6] and there is a lack of justification of such “proxy” model would work routinely [35]. Therefore, New statistical approaches or measures should be proposed to identify the true model from proxy models like how clearly current measures differentiate the true model from the flip-coin model. Such proposals might consist of adjusting current measures (e.g., with the degree of freedom) to penalty models using many proxy predictors to achieve high performance rather than causal predictors, in-direct probabilistic approach such as using machine learning to predict the probability of a model being the true model, and integrated approaches to comparing model with various measures systematically rather than individual face-value comparisons.

This study has strength that it conducts large simulations and case study analysis end up with consistent results. Yet more performance measures (e.g., calibration plots) or statistical approaches (e.g., likelihood ratio test) might be considered, but the study has covered conventional model performance measures (e.g., other calibration measures in eTable 1 and certain measures considered likelihood). More simulation might be considered but this study covered both of univariate and multivariate simulation such that for scenario with more than two predictors might be reduced to them with linear predictor. More external validation might be done as previously [3, 9, 30, 31] to further confirm the generalisability of the results. Certain candidate models with model selection procedure (e.g., stepAIC) in univariate simulation has reduced to the true model, which leads to compare the true model to itself, but the effects were small with 0.71% among all models in univariate simulation. Range of simulation parameters may be expanded, but the current range was suggested to cover the basic range of practical cases in medical/biological analysis [9]. Future study might investigate other type of outcome such as survival, other important complexities in the true model such as reverse causation, non-linear relationships or temporal dependencies, the effects of unmeasured confounding and data quality issues (e.g., sampling bias) on identifying the true model and the practical existence of the statistical true model in medicine/biology practical cases.

In conclusion, the study found the true model is not always identified as the “outperformed” model by current conventional measures for binary outcome even though such true model is presented in the clinical data. New statistical approaches or measures should be established to identify the true model from proxy models especially for those in proxy models with extra noises and/or missing causal predictors.

## Electronic supplementary material

Below is the link to the electronic supplementary material.

Supplementary Material 1

## Data Availability

Data generation process is either provided within the manuscript and supplementary information files, or can be accessed from the open accessed database (NHANES in this case).
