# Supplementary material for: Identify the underlying true model from other models for clinical practice using model performance measures
Source: BMC Med Res Methodol. 2025 Jan 9;25:4. doi: 10.1186/s12874-025-02457-w (PMC11715858; doi:10.1186/s12874-025-02457-w)
Supplement: Supplementary file 1 — Supplementary Material 1 [file 12874_2025_2457_MOESM1_ESM.docx]

**Supplementary Online Content**

Identify the underlying true model from other models for clinical practice using model performance measures

Published online XXX

Doi: XXXX

[**eFigure I**](#eFigure1)**: Overall workflow of the current study and future research directions**

[**eFigure II – part1**](#eFpt1)**: Diagram of overall methods part1**

**[eFigure II – part2](#eF2_pt2): Diagram of overall methods part2**

[**eFigure III**](#eFigureIII)**: Diagram of simulation process using multivariate simulation as exemplar (two variables in the true model are continuous)**

**[Appendix I](#ap1). Predictors and CVD outcome considered in the case study**

[**Appendix II**](#ap2)**. Simulating the outcome variable using true model and additional predictors for candidate models**

[**eTable 1**](#etable1_1)**: Brief description of model performance measurements**

[**eTable 2**](#etable1_2)**: Detailed description of model performance measurements**

[**eTable 3**](#etable2)**: Description of simulation parameters**

**[eTable 4](#etable31): Description of type of models that were compared in the simulation**

**[eTable 5](#etable32): Description of type of models that were compared in the case study analysis**

[**eTable 6**](#etable6)**: Summary of variables with missing values in the case study (patients aged 25-80 years who participated in NHANES 2017-2018)**

[**eTable 7**](#etable41)**: Comparison of the model performance between the pseudo true model and other proxy models in NHANES 2017-2018 - part II**

[**eTable 8**](#etable42)**: Comparison of the model performance between the multivariate pseudo true model and other proxy models in NHANES 2017-2018 - part I**

[**eTable 9**](#etable43)**: Comparison of the model performance between the multivariate pseudo true model and other proxy models in NHANES 2017-2018 - part II**

[**eTable 10**](#etable10)**: Comparison of the model performance between the pseudo true model (considering interaction effects of age and sex) and other proxy models in NHANES 2017-2018 - part I**

[**eTable 11**](#etable11)**: Comparison of the model performance between the pseudo true model (considering interaction effects of age and sex) and other proxy models in NHANES 2017-2018 - part II**

**[eFigure 1.2 – 1.25](#eFigure1x): Boxplot of differences of each model performance measure from candidate models to the true model in univariate simulations**

X axis: type of models

Y axis: Differences of each model performance measure from candidate models to the true model

[**eFigure 2.2 – 2.25**](#eFigure2x)**: Boxplot of differences of each model performance measure from candidate models to the true model in multivariate simulations**

X axis: type of models

Y axis: Differences of each model performance measure from candidate models to the true model

[References](#References)


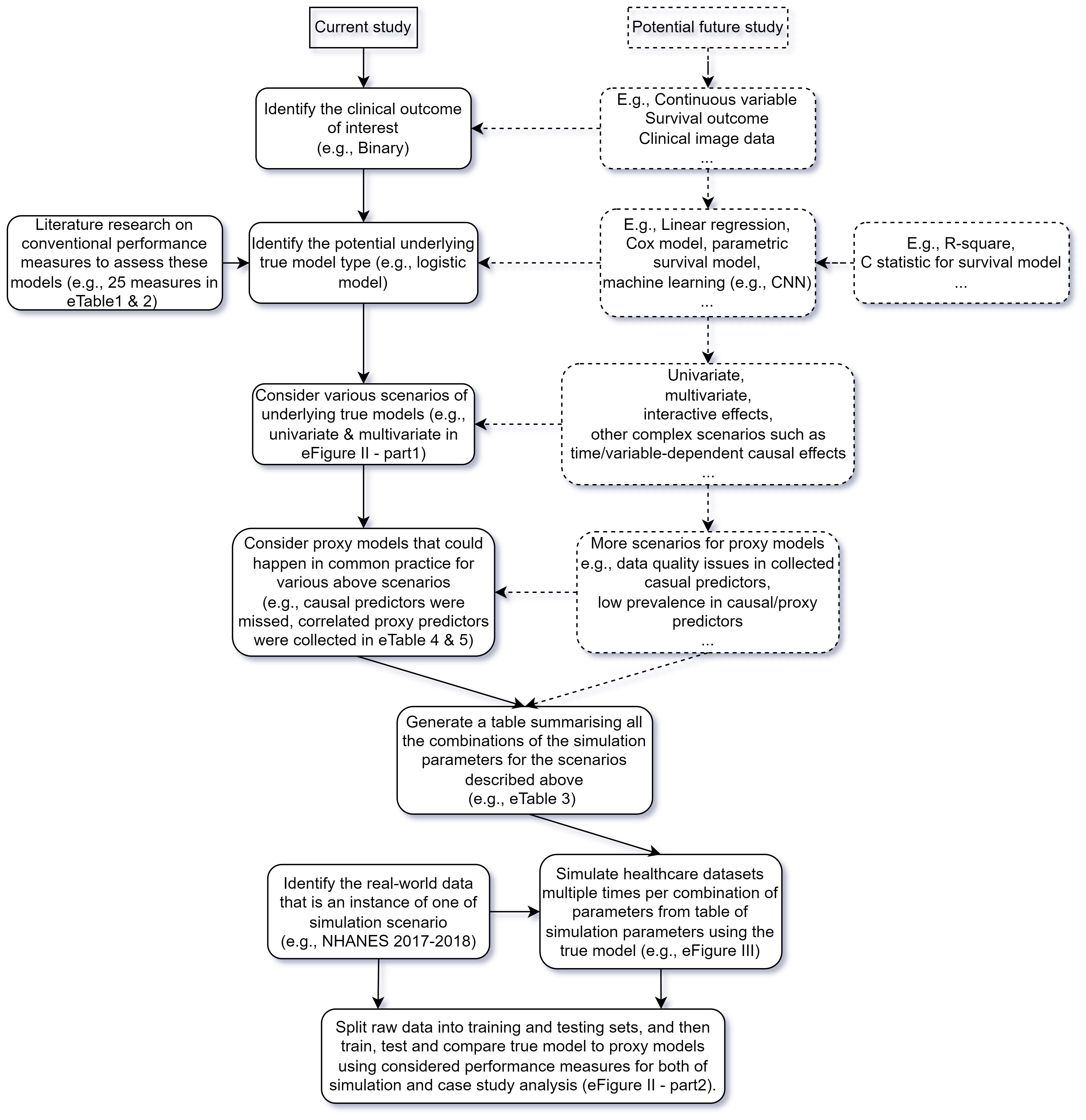


**eFigure I: Overall workflow of the current study and future research directions**


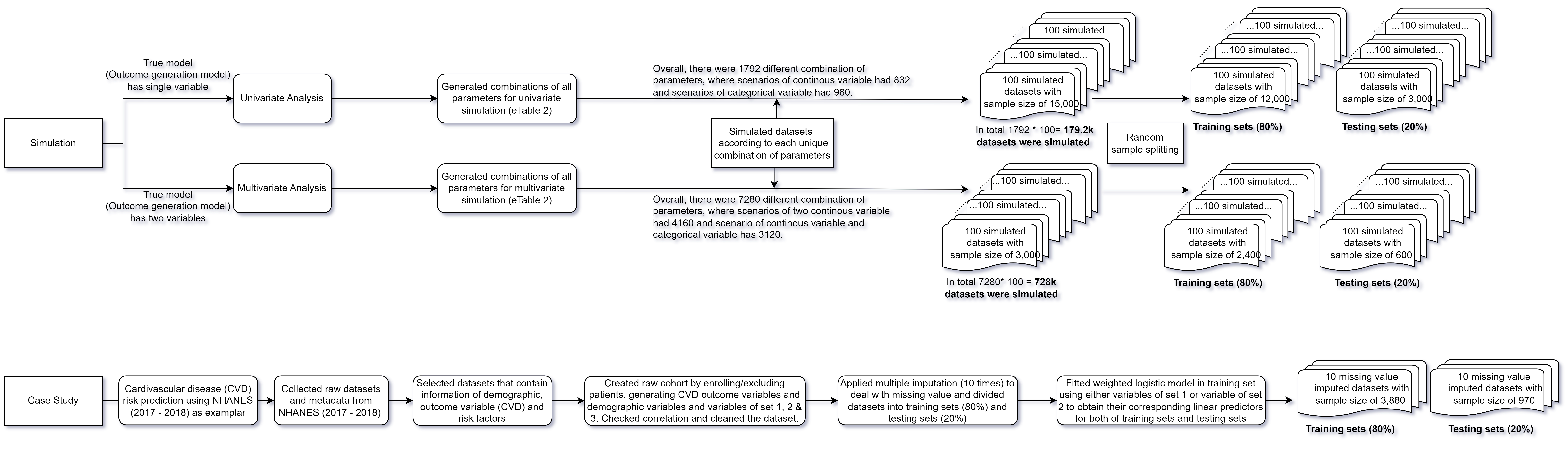


**eFigure II – part1: Diagram of overall methods part1**


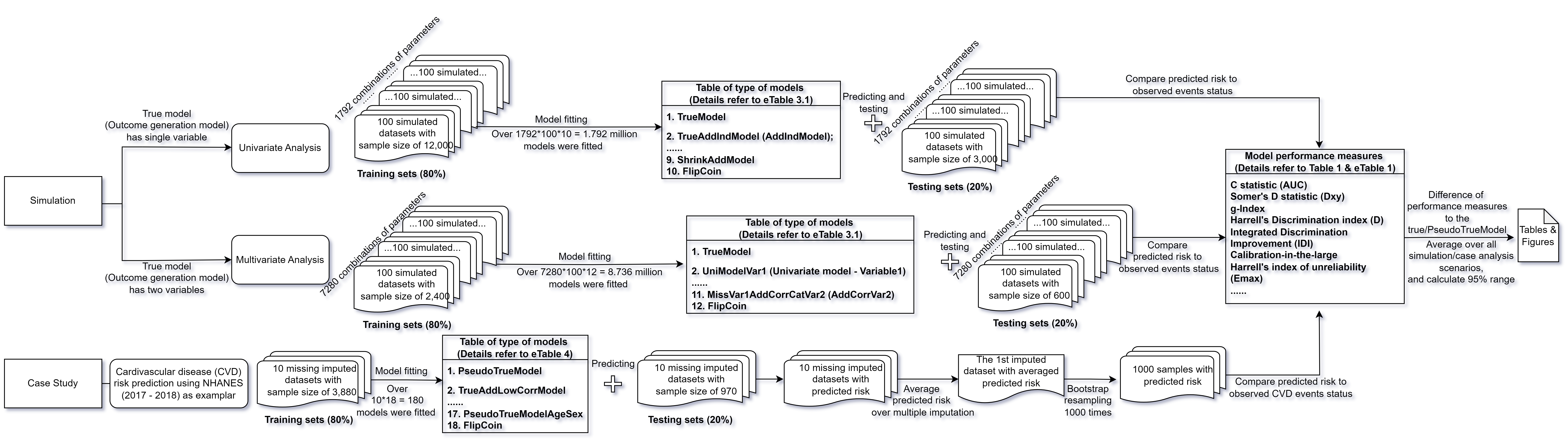


**eFigure II – part2: Diagram of overall methods part2**


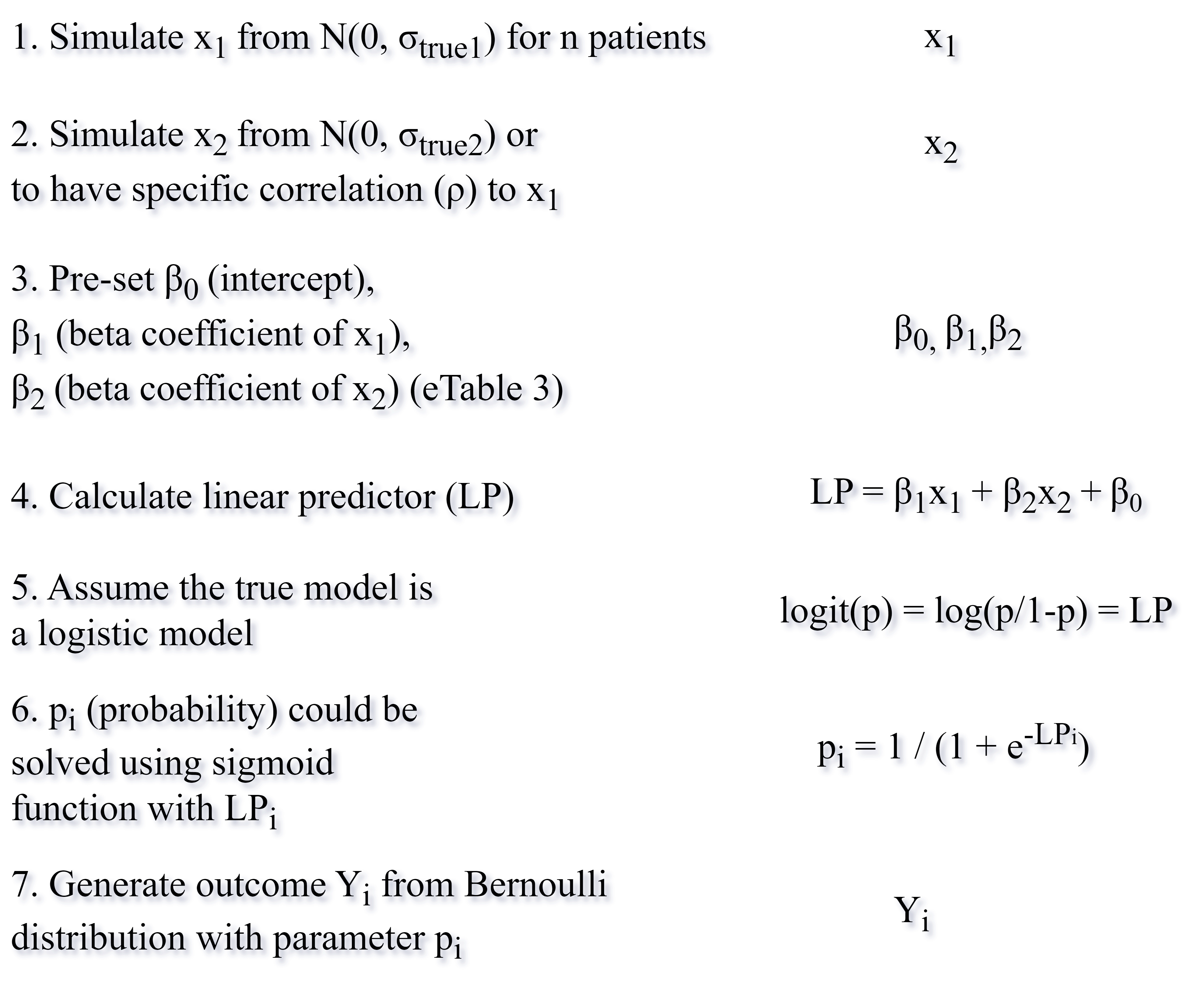


**eFigure III: Diagram of simulation process using multivariate simulation as exemplar (when two variables in the true model are continuous)**

**Note**:

1. The simulation process of univariate simulation is like above where there is only one variable in the true model.

2. When $x_{1}$ or $x_{2}$ are categorical then they were dichotomised from the simulated continuous variable.

3. Additional variables such as x_3_ (this was x_2_ for univariate simulation) were then simulated for candidate models with given parameters ([eTable 4](#etable31)).

**Appendix I. predictors and CVD outcome considered in the case study**

**Predictor set1**:

Gender, Age, Ethnicity, SBP, DBP, BMI, Ratio of Cholesterol and HDL, whether patient is on treatment of high blood pressure, Diabetes, Kidney disease, whether close relatives had heart attack?

**Predictor set2:**

Ratio of family income to poverty, Pulse, Stiffness of kidney, Albumin, Cotinine, Glycohemoglobin, HS C-Reactive Protein, Ratio of AST and ALT, Sleep hours at night

**Predictor set3:**

Marital status, Education level, Creatinine, Ferritin, Mercury, Vitamin A, Vitamin E, Vitamin C, Total Vitamin D

**Rationale of Predictor set1 (assume to be causal in Table 4 & eTable 7 - 9)**:

They were included in the external validated and clinical implemented QRISK3 CVD model.

**Rationale of Predictor set2 (assume to be causal in eTable 8 & 9):**

These are predictors that might have effects on CVD risk prediction but not directly in QRISK3 yet.

**Ratio of family income to poverty**: a measure of deprivation.

**Pulse**: pervious study has assessed its effects on CVD ^1^.

**Stiffness of kidney**: another direct measure to kidney health.

**Albumin**: Study has shown its relevance to CVD ^2^.

**Cotinine**: Study has shown its relevance to CVD ^3^.

**Glycohemoglobin**: Study has shown its relevance to CVD ^4^.

**HS C-Reactive Protein**: Study has shown its relevance to CVD ^5^.

**Ratio of AST and ALT**: Study has shown its relevance to CVD ^6^.

**Sleep hours at night:** Literature review suggests adequate sleep might help prevent CVD ^7^.

**Rationale of Predictor set3 (assume to be proxy predictors):**

These predictors might have effects to CVD but with more uncertainty comparing to set 1 & 2. Part of them could be noises.

**The 2^nd^ part: How CVD outcome was defined:**

This study referred QRISK3 ^8^ for CVD outcome definition (i.e., coronary heart disease, ischaemic stroke, or transient ischaemic attack (TIA)). The CVD outcome was derived in combination of questionnaire and ICD-10-CM drug usages that relates to CVD.

**In summary:**

**CVD patients:** have any records from CVD related questionnaire data and drug-usage

**Non-CVD (healthy) patients**: those did not meet any above CVD criteria and with non-missing negative answer for questionnaire of MCQ160b - MCQ160f.

**33 patients were removed as their CVD outcome cannot be determined**: those did not meet any above CVD criteria but have any missing value for questionnaire of MCQ160b - MCQ160f.

**Any CVD related questionnaire data:**

**Patients who have any positive answer for the following questions would be defined as having CVD:**

   MCQ160b - Ever told had congestive heart failure

   MCQ160c - Ever told you had coronary heart disease

   MCQ160d - Ever told you had angina/angina pectoris

   MCQ160e - Ever told you had heart attack

   MCQ160f - Ever told you had a stroke

**Patients who have any non-missing answer (excluded answer of “Refused” & “Don't know”) for following questions were defined as having CVD, as it relates to when did they have CVD:**

   MCD180b - Age when told you had heart failure

   MCD180c - Age when told had coronary heart disease

   MCD180d - Age when told you had angina pectoris

   MCD180e - Age when told you had heart attack

   MCD180f - Age when told you had a stroke

**Any CVD related drug usage records coded in** ICD-10-CM.

NHANES records up to three ICD-10-CM codes based on the description of certain drug usage according to various reasons. Patients who have any of these three ICD-10-CM codes which contain below CVD related drug codes would be defined as having CVD. The codes were the same to ICD-10 codes used in QRISK3:

G45 (transient ischaemic attack and related syndromes),

I20 (angina pectoris),

I21 (acute myocardial infarction),

I22 (subsequent myocardial infarction),

I23 (complications after myocardial infarction),

I24 (other acute ischaemic heart disease),

I25 (chronic ischaemic heart disease),

I63 (cerebral infarction),

I64 was not included in ICD-10-CM and also not recorded by NHANES

**33 patients were removed as their CVD outcome cannot be determined:**

Patients who have not yet been identified as CVD following above criteria (i.e., no positive non-missing answer for any of the questionnaire and no drug records relate to CVD), but has any missing value in MCQ160b - MCQ160f were considered as their CVD outcome status cannot be fully determined. This is because any missed positive answer for any of these questions (MCQ160b - MCQ160f) would flip these “healthy” patients into CVD patients. This may have noticeable impact on risk prediction model, as potential CVD patients were misclassified as non-CVD. There were 33 such patients ((33 / 4883 * 100) = 0.68%) were removed due to their CVD outcome cannot be fully determined.

MCD180b - MCD180f (age when told to have CVD) were not used for further excluding patients whose outcome cannot be determined. This is because it is reasonable a patient may answer “No” to all of MCQ160b - MCQ160f questions and leave blanks to “MCD180b - MCD180f” since they might think they are unapplicable. Therefore, non-CVDs have all non-missing negative answer for MCQ160b - MCQ160f but could have missing value for MCD180b - MCD180f.

**Appendix II -** Simulating the outcome variable using true model and additional predictors for candidate models

**Methods**: Simulating the outcome variable using true model

For univariate simulation, when the causal predictor was continuous in the true model, it was simulated from a normal distribution with 0 mean and a pre-set standard deviation ($\sigma_{true}$) ^9^. When the predictor was binary, it was dichotomised with a pre-set proportion ($p_{true}$) from a simulated underlying continuous predictor follows normal distribution with 0 mean and a pre-set standard deviation ($\sigma_{true}$) ^9^. This enables later on to generate other underlying continuous predictors (for other added categorical/binary predictors) which have certain correlations (e.g., 0.3) to the underlying continuous predictor of this binary predictor in the true model ^9^. For univariate simulation where the single causal predictor was continuous (follows a normal distribution with 0 mean and $\sigma_{true}$ as the standard deviation). It assumed the variation of this causal predictor may range from 0.25^2^ to 4^2^ in practice. $\beta_{1}$ was the true causal effects of this causal predictor on the outcome binary variable, and this assumed to have range from log(1) to log(4) in practice. $\beta_{0}$ was the intercept of the linear predictor in the causal logistic true model and assumed to be -1 ^9^ ([eTable 3](#etable2)).

For multivariate simulation, when the two predictors of the true model were both continuous and uncorrelated, the first continuous variable was simulated from a normal distribution with 0 mean and a pre-set standard deviation ($\sigma_{true1}$), the second continuous variable was simulated from a standard normal distribution ([eFigure III](#eFigureIII)). When they were correlated, the second continuous variable were simulated to have specific correlations (ρ) such as 0.3 to the first continuous predictor ^9^. When the two predictors of the true model were continuous and categorical, similar process as above was done to simulate one continuous variable and one underlying latent continuous variable, the categorical variable with proportion parameter p ($p_{true}$) was then obtained by dichotomising the underlying latent continuous variable.

A linear predictor could then be calculated with the sum of multiplication of pre-set beta coefficients (e.g.,$\beta_{1}$) and the causal predictors in the true model plus the intercept ($\beta_{0}$). Probability (i.e., $p_{i}$ ) of the $i_{th}$ observation (patient) having the binary outcome could be calculated using the sigmoid function ^10^ (i.e., solve $p_{i}$ from the logit function ^11^) with the calculated linear predictor. The binary outcome for the $i_{th}$ observation was then randomly generated from the Bernoulli distribution give parameter $p_{i}$ ^9^ for multiple times. Above illustrated how the outcome variable was simulated from the outcome generation true model which described the exact relationship between the outcome variable and causal predictors.

**Methods**: Simulated additional predictors for candidate models.

For univariate simulation, added variable $x_{2}$ could be uncorrelated or correlated (e.g., correlation of 0.3, 0.5) to the single predictor ($x_{1}$) in the true model. When$x_{2}$ was uncorrelated to $x_{1}$ and when $x_{2}$ was continuous it was simulated from a standard normal distribution. When it was categorical, it was dichotomised with parameter $p_{add}$ from a simulated underlying continuous variable assuming follows standard normal distribution. When$x_{2}$ was correlated to $x_{1}$, and when $x_{2}$ was continuous it was simulated to have the specific correlation to $x_{1}$, and when $x_{2}$ was categorical, an underlying latent continuous variable was first simulated to have the specific correlation to $x_{1}$ and then this variable was used to dichotomised to obtain $x_{2}$ given parameter $p_{add}$ ^9^.

E.g., For univariate simulation, the added proxy variable could be independent or correlated (e.g., correlation of 0.3 and 0.5) to the causal predictor in the true model. When the proxy predictor was continuous and independent of the causal predictor, it was randomly drawn from a standard normal distribution (i.e., σ_add_ = 1). When it was categorical and independent, it was dichotomised with parameter $p_{add}$ from a simulated underlying continuous variable assuming follows standard normal distribution ^9^. When the proxy predictor was continuous and correlated to the causal predictor with a pre-specified correlation (e.g., 0.3), it was simulated to have that specific correlation to the causal predictor. When the proxy predictor was binary, an underlying latent continuous variable was first simulated to have the specific pre-specified correlation to the causal predictor, and then this variable was dichotomised to obtain the binary proxy predictor given parameter $p_{add}$ ^9^.

Similar simulation process applied to the additional predictor $x_{3}$ for multivariate simulation, except that more scenarios were considered such as $x_{3}$could be correlated to either predictor (i.e., $x_{1}$ and $x_{2}$) in the true model while could also be either continuous or categorical ([eTable 4](#etable31) describes the correlation between proxy predictors and causal predictors through model formulas).

**eTable 1: Description of model performance measurements ***

|  | **Description** | **Interpretation** |
| --- | --- | --- |
| **Discrimination** | | |
| C statistic (AUC) | C statistic, also known as Area Under the Curve (AUC), measures the ability of model to rank patients with events as higher probability comparing to non-events (healthy patients) as lower probability (i.e., discriminative ability (concordance between predicted risk and observed outcome)) ^12^ . It calculates a proportion of correctly ranked pairs of events and non-events among all random pairs of patients in the population ^13^. | Value of C statistic ranges from 0 to 1. The higher C statistic suggests model has higher discriminative ability. A model with C statistic below or equal to 0.5 suggests the discriminative probability of the model is worse than or equivalent to flip a fair coin (i.e., random prediction). |
| Somer's D statistic (Dxy) | Somers’ D statistic is a rank correlation between model’s predicted probability and the observed outcome ^14^, which also measures discriminative ability of model. The D statistic can be calculated by 2 * (C - 0.5) for binary logistic model, where C represents C statistic ^15^. | Value of D statistic ranges from -1 to 1, where D statistic below or equal to 0 suggests model has less or equivalent discriminative probability as a flip fair coin. D statistic has value as 1 suggests perfect discrimination. |
| g-Index | g-Index is a measure of discrimination based on Gini’s difference (i.e., the average of absolute difference of values of all possible pairs of a variable). In the binary prediction case, the variable is defined as predicted probability ^15^, thus g-Index measures the averaged absolute difference in predicted probability among all possible pairs. | g-Index in the binary prediction case ranges from 0 to 1. g-Index equals or close to 0 could mean model fails to separate high risk patients from low risk (i.e., no discrimination) or the patients are hard to discriminate. larger value of g-Index suggests higher level discriminative. |
| Harrell's Discrimination index (D) | Harrell's Discrimination index (D) measures the discrimination of models by calculating differences of -2 log likelihood between an intercept only model (i.e., beta coefficient of linear predictor from original model was set to 0) and a re-calibrated model (i.e., both overall intercept and slope are re-calibrated) using linear predictor from the original model. | Harrell's Discrimination index (D) has value of or close to 0 suggests the model has no or almost zero discrimination, this means predictors considered in the model has no effects and adds no discriminative ability to the model comparing to a model which assumes effects of all predictors equal to 0 (i.e., the intercept only model). |
| Integrated Discrimination Improvement (IDI) | IDI measures the improvement of Integrated discrimination comparing one model (Usually the model with new predictor) to another. This was achieved by taking the difference of differences in the average predicted risk between events and non-events of the two models. | The IDI measure ranges from -1 to 1. An IDI value of 0 indicates that there is no improvement in integrated discrimination of the new model compared to the reference model. An IDI greater than 0 suggests that the new model has higher integrated discrimination than the reference model, while a negative IDI implies that the new model has worse integrated discrimination than the reference model. |
| **Calibration** | | |
| Calibration-in-the-large | Calibration-in-the-large compares overall averaged risk predictions to observed events rate (i.e., the difference between overall averaged predicted risk and observed events rate). | Calibration-in-the-large equivalents or close to 1 suggests model is well calibrated in overall general, as the overall predicted risk agrees to observed events rate. Calibration-in-the-large is below or above 1 indicates model is mis-calibrated by underestimating or overestimating risk of patients in overall general. |
| Harrell's index of unreliability (Emax) | Emax is the maximum absolute difference between predicted risk and re-calibrated predicted risk (calculated from the re-calibrated model using the linear predictor from the original model) given the range of predicted risk ^15^, which measures the maximum prediction error that the model has made comparing to the re-calibrated predicted risk. The range of predicted risk could be chosen either as minimum and maximum of predicted risk (say, $\hat{p}_{min}$ and $\hat{p}_{max}$) or full range of all possible predicted risk (i.e., between 0 and 1). | Emax equivalents or close to 0 suggests the model is well calibrated as the model predicts risks agree to calibrated predicted risk in general and no re-calibration is needed, whereas the larger Emax value suggests more likely the model needs to be re-calibrated. |
| Harrell's E statistic (Eavg) | Eavg is the averaged absolute differences between predicted risk and probability obtained by smoothing observed outcome against predicted risk, which also measures the magnitude of agreement between predicted risk and observed events rate (i.e., calibration). | Eavg equivalents or close to 0 suggests the model is well calibrated as the model predicts risks agree to the smoothed observed probability, whereas large value in Eavg suggests model need to be re-calibrated. |
| Estimated calibration index (ECI) | ECI is a similar concept as Eavg in the sense that it also considers differences between predicted risk and probability obtained by smoothing observed outcome against predicted risk, but it considers the square root of the difference rather than absolute difference. | Like Eavg, ECI equivalents or close to 0 suggests the model is well calibrated. |
| Harrell's Unreliability index (U) | Harrell's Unreliability index (U) ^15^ measures the unreliability of models by calculating differences of -2 log likelihood between an uncalibrated model (i.e., the intercept is set to 0 and beta of linear predictor is set to 1) and a re-calibrated model (i.e., both overall intercept and slope are re-calibrated using linear predictor from the original model). | Unreliability index U have value as or close to 0 suggests predictions are reliable as by definition there is no likelihood difference between refit/recalibrate model and the original model. |
| **Overall model performance measures** | | |
| Brier score | Brier score compares predicted risk to observed outcome, which is known as quadratic scoring rule ^12^ ^15^ ^16^. It measures overall prediction error of model (i.e. including both of discrimination and calibration as it can be formally decomposed into them ^12^ ^15^). | Brier score ranges from 0 to 1, and the lower value of Brier score suggests the model has better overall model performance. |
| Pesudo R-square | Pseudo R^2^ is a generalisation of R^2^ from linear model to generalised linear model. It measures overall model performance. | Pseudo R^2^ normally ranges from 0 to 1 (except say CoxSnell R-square ^17^), and the higher value suggests better overall model performance ^12^. There is current no consensus which pseudo R^2^ is more appropriate ^18^, and one should not compare face value of one version of pseudo R^2^ to another version directly. |
| Harrell's overall quality index (Q) | Harrell's overall quality index (Q) is an overall summary measure to quantify the overall quality of model’s predictions. It can be computed with Unreliability index (U) and Discrimination index (D) by "Q = discrimination - total unreliability = D – U”. The index is also known as logarithmic accuracy score. | The value of overall quality index (Q) could be interpreted with discrimination index (D) and Unreliability index (U). A well discriminated and reliable model would have D away from 0 and U close to 0 which suggests value of Q is close to value of D and away from 0. |
| **Other model performance measures** | | |
| Global Shrinkage Factor | Global Shrinkage Factor could measure overfitting of model ^17^. Model overfitting means the trained model specialised on the training dataset, thus could not be generalised to a plausible related external dataset (i.e., such model would have very high performance on training dataset but poor performance on external dataset) ^12^. | Global shrinkage factor ($S_{global}$) is close to 1 indicates less likely the model needs to be refitted/recalibrated. Empirical analysis proposed $S_{global}$ ≥ 0.9 as one of criteria in sample size calculation ^17^, as it indicates less likely model needs recalibration. |
| * More detailed description of model performance measurements could be found from eTable 2 | | |

**eTable 2: Detailed description of model performance measurements**

|  | **Description** | **Interpretation** |
| --- | --- | --- |
| **Discrimination** | | |
| C statistic (AUC) | C statistic, also known as area under the curve (AUC), measures the ability of model to rank patients with events as higher risk comparing to non-events (healthy patients) as lower risk (i.e., discriminative ability (concordance between predicted risk and observed outcome)) ^12^ . It calculates a proportion of correctly ranked pairs of events and non-events among all random pairs of patients in the population ^13^. | Value of C statistic ranges from 0 to 1. The higher C statistic suggests model has higher discriminative ability. A model with C statistic below or equal to 0.5 suggests the discriminative probability of the model is worse than or equivalent to flip a fair coin (i.e., random prediction). For example, an overfitted model may perform worse than random prediction in the external dataset. C statistic equals 1 suggests the model perfectly discriminates all events and non-events. However, model with perfect discrimination ability does not necessarily mean it predicts patients perfectly, as the calibration of such model could be poor ^12^. It was suggested that a model with C statistic above 0.8 may have some level of clinical utility for individual patients ^15^, e.g., an implemented cardiovascular disease (CVD) risk prediction model QRISK3 has C statistic above 0.8 ^19^. |
| Somer's D statistic (Dxy) | Somers’ D statistic is a rank correlation between model’s predicted probability and the observed outcome ^14^, which also measures discriminative ability of model. The D statistic can be calculated by 2 * (C - 0.5) using C statistic ^15^ for binary outcome. | Value of D statistic ranges from -1 to 1, where D statistic below or equal to 0 suggests model has less or equivalent discriminative probability to a flip fair coin. This is consistent to C statistic when value of C statistic below or equal to 0.5. Similarly, D statistic has value as 1 suggests perfect discrimination of model as C statistic equals to 1. |
| g-Index | g-Index is a measure of discrimination based on Gini’s difference (i.e., the average of absolute difference of values of all possible pairs of a variable). For the binary outcome, the variable is defined as predicted probability ^15^, thus g-Index measures the averaged absolute difference in predicted probability among all possible pairs, which can be written as $\frac{1}{m}\sum_{i \neq j} \left\vert\hat{p}_{i} - \hat{p}_{j} \right\vert$, where m is the number of all possible pair (i.e., combination of choosing 2 from n patients, which is $m= C_{n}^{2}=\frac{n (n-1)}{2}$) and $\hat{p}_{i}$, $\hat{p}_{j}$ is the predicted probability for the i-th or the j-th patient among n patients ^15^. | For the binary outcome, the range of g-Index ranges from 0 to 1. g-Index equals or close to 0 means model predicts very similar probability to all patients irrespective of events and non-events, which could mean model fails to separate high risk patients from low risk (i.e., no discrimination) or the patients are hard to discriminate (where in this case there might be some discrimination left but all predicted probability are still similar). Similarly larger value of g-Index implies higher level discriminative ability of model separate high-risk patients from low-risk patients on average. |
| Harrell's Discrimination index (D) | Harrell's Discrimination index (D) ^15^ ^20^ measures the discrimination of models by calculating differences of -2 log likelihood between an intercept only model (i.e., set beta coefficient of linear predictor from original model to 0, also known as best constant predictor model) and a re-calibrated model (i.e., both overall intercept and slope are re-calibrated using linear predictor from the original model). Suppose we define ℒ (a, b) as the -2 log likelihood of a re-calibrated model using linear predictor of the original model, where “a” represents the intercept and “b” represents the slope ^15^. Following -2 log likelihoods could be obtained using model formula from R “glm” package:  ℒ (a, b) = -2 log likelihood of the recalibrated model using formula as “outcome ~ linear predictor”.  ℒ (a, 1) = -2 log likelihood of the recalibrated model using formula as “outcome ~ offset (linear predictor)”, where beta of linear predictor is fixed to 1.  ℒ (a, 0) = -2 log likelihood of the recalibrated model using formula as “outcome ~ 1”, where beta of linear predictor is set to 0.  ℒ (0, 1) = -2 log likelihood of the uncalibrated model using formula as “outcome ~ offset(linear predictor) - 1”, where beta of linear predictor is set as 1, and intercept is set to 0 as “-1” in R formula means suppress the intercept.  Then one can define Harrell's index including Discrimination index (D), Unreliability index (U) and Overall summary index (Q) as ^15^:  $D=\frac{\left( \mathcal{L}\left( a, 0 \right)- \mathcal{L}\left( a, b \right)-1 \right)}{n}$  $U=\frac{\left( \mathcal{L}\left( 0, 1 \right)- \mathcal{L}\left( a, b \right)-2 \right)}{n}$  $Q=\frac{\left( \mathcal{L}\left( a, 0 \right)- \mathcal{L}\left( 0, 1 \right)+1 \right)}{n}$  , Where n represents number of observations in the dataset. | Harrell's Discrimination index (D) has value of or close to 0 suggests the model has no or almost zero discrimination, as by its definition this means predictors considered in the model has no effects and adds no discriminative ability to the model comparing to a model which assumes effects of all predictors equal to 0 (i.e., an intercept only model). |
| Integrated Discrimination Improvement (IDI) | IDI measures the improvement of integrated discrimination comparing one model (usually the model with new predictor) to another. This was achieved by taking the difference of differences in the average predicted risk between events and non-events of the two models. In detail,  $IDI=\left( {\bar{\hat{p}}}_{model2, events}- {\bar{\hat{p}}}_{model2, non-events} \right)- \left( {\bar{\hat{p}}}_{model1, events}- {\bar{\hat{p}}}_{model1, non-events} \right)$ , where ${\bar{\hat{p}}}_{model, events}, {\bar{\hat{p}}}_{model, non-events}$ means the averaged predicted risk of the model among events or non-events accordingly. In this study, the reference model (model1) was selected as the data generation model, so all other models were compared to the reference model to see any improvement in integrated discrimination. | The IDI measure ranges from -1 to 1 because it is calculated as the difference of probabilities. An IDI value of 0 indicates that there is no improvement in integrated discrimination of the new model compared to the reference model. An IDI greater than 0 suggests that the new model has higher integrated discrimination than the reference model, while a negative IDI implies that the new model has worse integrated discrimination than the reference model. |
| **Calibration** | | |
| Calibration-in-the-large | Calibration-in-the-large compares overall averaged risk predictions to observed events rate (i.e., the difference between overall averaged predicted risk and observed events rate). In binary case, this could be calculated as dividing odds of overall averaged predicted risk (i.e., overall averaged predicted risk of events dividing by overall averaged predicted risk of nonevents) by odds of observed risk ^12^. The formula is ^12^:  $Odds Ratio \left( OR \right)= \frac{\frac{\overline{\hat{y}}}{1-\overline{\hat{y}}}}{\frac{\bar{y}_{obs}}{1-\bar{y}_{obs}}}$  Where $\overline{\hat{y}}$ represents overall averaged predicted risk of events (i.e., mean($\hat{y}$)) and $\bar{y}_{obs}$ represents observed events rate. | Calibration-in-the-large equivalents or close to 1 suggests model is well calibrated in overall general, as the overall predicted risk agrees to observed events rate. Calibration-in-the-large is below or above 1 indicates model is mis-calibrated by underestimating or overestimating risk of patients in overall general. |
| Harrell's index of unreliability (Emax) | Emax is the maximum absolute difference between predicted risk and re-calibrated predicted risk (calculated from the re-calibrated model using the linear predictor from the original model) given the range of predicted risk ^15^, which measures the maximum prediction error that the model has made comparing to the re-calibrated predicted risk. The range of predicted risk could be chosen either as minimum and maximum of predicted risk (say, $\hat{p}_{min}$ and $\hat{p}_{max}$) or full range of all possible predicted risk (i.e., between 0 and 1), which is corresponding to Emax($\hat{p}_{min}$, $\hat{p}_{max}$) (Emax_ab) and Emax(0, 1) ^15^ (Emax_01). This can be calculated by ^15^  $\mathrm{Emax}\left( \hat{p}_{min}, \hat{p}_{max} \right)= \max_{\hat{p}_{min}\leq\hat{p} \leq\hat{p}_{max}} \left\vert\hat{p}- \hat{p}_{recal} \right\vert$  Where$\hat{p}$ represents predicted risk and $\hat{p}_{recal}$ represents predicted risk from re-calibrated model. Also, the study calculated the averaged absolute difference between predicted risk and re-calibrated predicted risk considering different range ($\hat{p}_{min}$ and $\hat{p}_{max}$) (E_abMean) or (0, 1) (E_01Mean) to further assess the calibration. | Emax equivalents or close to 0 suggests the model is well calibrated as the model predicts risks agree to calibrated predicted risk in general and no re-calibration is needed, whereas the larger Emax value suggests more likely the model needs to be re-calibrated. |
| Harrell's E statistic (Eavg) | Eavg is the averaged absolute differences between predicted risk and probability obtained by smoothing observed outcome against predicted risk, which also measures the magnitude of agreement between predicted risk and observed events rate (i.e., calibration). In detail, the smoothing process has been done to a scatter plot which plots observed outcome (i.e., 0 and 1 in this binary case) against to predicted probability. The smoothed curve was then used to interpolate the observed probability for each data point (i.e., for each predicted risk). Eavg is then calculated as averaged absolute difference between predicted risk and smoothed observed probability, which could be written as ^12^ ^15^:  $Eavg=\frac{1}{n}\sum_{i} \left\vert\hat{p}_{i} - \hat{p}_{{smooth}_{i}} \right\vert$  where n is the number of observations, $\hat{p}_{i}$ represents predicted probability of the i-th patient and $\hat{p}_{{smooth}_{i}}$ represents smoothing-interpolated probability of this patient. | Eavg equivalents or close to 0 suggests the model is well calibrated as the model predicts risks agree to the smoothed observed probability, whereas large value in Eavg suggests model need to be re-calibrated. The study considered Locally Weighted Scatterplot Smoothing (lowess) ^21^ technique for smoothing to calculate ECI. |
| Estimated calibration index (ECI) | ECI is a similar concept as Eavg in the sense that it also considers differences between predicted risk and probability obtained by smoothing observed outcome against predicted risk, but it considers the square root of the difference rather than absolute difference. In binary case, this could be written as ^12^ ^22^ ^15^:  $\mathrm{ECI}=\frac{1}{n}\sum_{i} \left( \hat{p}_{i} - \hat{p}_{{smooth}_{i}} \right)^{2}*100$  where n is the number of observations, $\hat{p}_{i}$ represents predicted probability of the i-th patient and $\hat{p}_{{smooth}_{i}}$ represents smoothing-interpolated probability of this patient. The reason to multiply 100 is to make the index within the range of 0 to 100 ^22^. | Like Eavg, ECI equivalents or close to 0 suggests the model is well calibrated, and the study calculated ECI using “lowess” ^21^ as the smoothing technique. |
| Harrell's Unreliability index (U) | Harrell's Unreliability index (U) ^15^ measures the unreliability of models by calculating differences of -2 log likelihood between an uncalibrated model (i.e., the intercept is set to 0 and beta of linear predictor is set to 1) and a re-calibrated model (i.e., both overall intercept and slope are re-calibrated using linear predictor from the original model). | Unreliability index U have value as or close to 0 suggests predictions are reliable as by definition there is no likelihood difference between refit/recalibrate model and the original model. |
| **Overall model performance measures** | | |
| Brier score | Brier score compares predicted risk to observed outcome, which is a strict proper scoring rule ^16^ (also known as quadratic scoring rule ^12^ ^15^). It measures overall prediction error of model (i.e. including both of discrimination and calibration as it can be formally decomposed into them ^12^ ^15^). In binary case, this can be written as ^12^ ^15^:  $\mathrm{BS}=\frac{1}{n}\sum_{i} \left( \hat{p}_{i} - y_{{obs}_{i}} \right)^{2}$  where n is the number of observations, $\hat{p}_{i}$ represents predicted probability of the i-th patient and $y_{{obs}_{i}}$ represents observed outcome of this patient. One can re-write its quadratic part as ^12^:  $\left( \hat{p}_{i} - y_{{obs}_{i}} \right)^{2}=y_{{obs}_{i}} \left( 1- \hat{p}_{i} \right)^{2}+(1-y_{{obs}_{i}}) {\hat{p}_{i}}^{2}$  which analogies to maximise log likelihood of logistic model, i.e., when $y_{{obs}_{i}}$ = 1 then right part equals to 0, and for left part the closer $\hat{p}_{i}$ to 1 results smaller Brier score, similar to when $y_{{obs}_{i}}$ = 0 then left part equals to 0, and for right part the closer $\hat{p}_{i}$ to 0 results smaller Brier score, thus Brier score measures overall model performance. | Brier score ranges from 0 to 1, and the lower value of Brier score suggests the model has better overall model performance. A model with Brier score equals to exact 0 suggests it has extreme prediction that predicts probability of events as 1 and non-events as 0 which may have poor generalisation to other data. |
| Pesudo R-square | Pseudo R^2^ is a generalisation of R^2^ from linear model to generalised linear model. In linear model, R^2^ is defined as variance explained by model dividing total variance (i.e., variance explained by model plus variance of error/residual). This means R^2^ measures how much/percentage of variance has been explained by the model among overall variances ^12^ ^18^. For linear model, the total variance is the sum of variance explained by model and variance from error/residual ^18^, but this may not be the case for generalised linear model as residuals may not be orthogonal to the predictions ^23^. In this case, multiple generalisations of R^2^ are proposed. Specifically, for binary logistic model, following Pesudo R^2^ were considered in this study:  **CoxSnell R-square** ^17^:  $R_{\mathrm{CoxSnell}}^{2}=1- e^{\left( \frac{-likelihood ratio}{n} \right)}= 1- e^{\left( \frac{-(-2(log{(L}_{null})- log{(L}_{model})))}{n} \right)}$  And the **maximum value** of $R_{\mathrm{CoxSnell}}^{2}$ is ^17^:  ${max(R}_{CoxSnell)}^{2}=1- e^{\left( \frac{2log{(L}_{null})}{n} \right)}$  **Nagelkerke** **R-square** ^17^**:**  $R_{\mathrm{Nagelkerke}}^{2}=\frac{1- e^{\left( \frac{-(-2(log{(L}_{null})- log{(L}_{model})))}{n} \right)}}{1- e^{\left( \frac{2log{(L}_{null})}{n} \right)}}=\frac{R_{\mathrm{CoxSnell}}^{2}}{{max(R}_{CoxSnell)}^{2})}$  **McFadden R-square** ^17^:  $R_{McFadden}^{2}=1- \frac{log{(L}_{model})}{log{(L}_{null})}$  **Adjusted McFadden R-square:**  $R_{McFadden}^{2}=1- \frac{log{(L}_{model})-N_{p}}{log{(L}_{null})}$  **AldrichNelson R-square** ^24^:  $R_{\mathrm{AldrichNelson}}^{2}=\frac{likelihood ratio}{likelihood ratio+n}$=  $\frac{-2(log{(L}_{null})- log{(L}_{model}))}{-2(log{(L}_{null})- log{(L}_{model}))+n}$  **VeallZimmermann R-square** ^24^:  $R_{\mathrm{VeallZimmermann}}^{2}=\frac{\frac{likelihood ratio}{likelihood ratio+n}}{\frac{-2log{(L}_{null})}{n- 2log{(L}_{null})}}$  $=R_{\mathrm{AldrichNelson}}^{2}*\frac{n-2log{(L}_{null})}{-2log{(L}_{null})}$  **Efron R-square** ^25^:  $R_{\mathrm{Efron}}^{2}= \frac{1- \sum_{i} \left( y_{{obs}_{i}} - \hat{p}_{i} \right)^{2}}{\sum_{i} \left( y_{{obs}_{i}} - \bar{y}_{obs} \right)^{2}}$  **McKelveyZavoina R-square** ^26^:  $R_{\mathrm{McKelveyZavoina}}^{2}= \frac{\sum_{i} \left( {lp}_{i} - \bar{lp} \right)^{2}}{\sum_{i} \left( {lp}_{i} - \bar{lp} \right)^{2}+n* \frac{2\pi}{3}}$  **Tjur R-square** ^23^:  $R_{\mathrm{Tjur}}^{2}=D= \bar{\hat{p}_{1}}-\bar{\hat{p}_{0}}$  Where n is the number of observations, $N_{p}$ represents number of predictive parameters considered in the model (i.e., all the terms derived from predictors such as interaction terms if considered and intercept ^17^), $L_{null}$ and $L_{model}$ are corresponds to likelihood of null model and fitted model, $\hat{p}_{i}$ represents predicted probability of the i-th patient, $y_{{obs}_{i}}$ represents observed outcome of this patient, $\bar{y}_{obs}$ represents observed events rate, ${lp}_{i}$ represents i-th linear predictor (i.e. sum of beta coefficients and predictor parameters) of the i-th patients, $\bar{lp}$ represents overall average of linear predictors of all patients, $\hat{p}_{1}$ represents averaged predicted risk for events and $\bar{\hat{p}_{0}}$ represents averaged predicted risk for non-events. | Pseudo R^2^ ranges from 0 to 1 in general, and the higher value suggests better overall model performance ^12^. There is current no consensus which pseudo R^2^ is more appropriate ^18^, and one should not compare one version of pseudo R^2^ to another version. It was reported that the max value of CoxSnell R-square could not reach to 1 even though the model is perfect, thus maximum value of CoxSnell R-square should be reported or Nagelkerke R-square (which divide CoxSnell R-square by its max value) should be used ^17^. It was also reported that VeallZimmermann R-square which is an adjusted version of AldrichNelson R-square better mimicked R^2^ of linear model among other pseudo R^2 24^. The study considered all these pseudo R^2^ in model comparison. |
| Harrell's overall quality index (Q) | Harrell's overall quality index (Q) is an overall summary measure to quantify the overall quality of model’s predictions, which is the differences of -2 log likelihood between an intercept only model (where beta of linear predictor is set to 0) and an uncalibrated model (i.e., both of overall intercept and slope are re-calibrated) using linear predictor from the original model. It can also be computed with Unreliability index (U) and Discrimination index (D) by "Q = discrimination - total unreliability = D – U”. The index is also known logarithmic accuracy score. | The value of overall quality index (Q) could be interpreted with discrimination index (D) and Unreliability index (U). A well discriminated and reliable model would have D away from 0 and U close to 0 which suggests value of Q is close to value of D and away from 0. A no discrimination model and unreliable model would have D close to 0 and U away from 0 which suggests Q is close to value of -U and away from 0. A no discrimination but reliable model would have both D and U close to 0 and Q is close to 0. A well discriminated model but unreliable model would have both of D and U stay away from 0 and value of Q depends on the differences of magnitude of how good the discrimination is and how bad the reliability is. |
| **Other model performance measures** | | |
| Global Shrinkage Factor | Global Shrinkage Factor could measure overfitting of model ^17^. Model overfitting means the trained model specialised on the training dataset, thus could not be generalised to a plausible related external dataset (i.e., such model would have very high performance on training dataset but poor performance on external dataset) ^12^. The measure could be estimated by ^17^ ^27^:  $S_{global}=1- \frac{N_{p}}{likelihood ratio}$  $=1- \frac{N_{p}}{-2(log{(L}_{null})- log{(L}_{model}))}$  where $N_{p}$ represents number of predictive parameters considered in the model and $L_{null}$ and $L_{model}$ are corresponds to likelihood of null model and fitted model. | Global shrinkage factor ($S_{global}$) is close to 1 indicates less likely the model needs to be refitted/recalibrated, as this means there is reasonable amount of difference between the fitted model and the null model, in which case, the right side of the formula would be close to 0 as the denominator is reasonable large. However, if there is little difference of log likelihood between the fitted model and the null model, $S_{global}$ could result either extreme small negative value or extreme large positive value given which log likelihood is slight larger. Say if log likelihood of fitted model is only slightly larger than the log likelihood of null model, then this results extreme small negative value of $S_{global}$, which suggests model is more likely overfitting as it is worse than the null model. Empirical analysis proposed $S_{global}$ ≥ 0.9 as one of criteria in sample size calculation ^17^, as it indicates less likely model needs recalibration. |

| **eTable 3: Description of simulation parameters @** | | |
| --- | --- | --- |
|  | | |
| **Type of models** | **Explanation** | **Range of values** |
| **True model is a univariate model (Univariate simulation)** | | |
| **Univariate true model (continuous variable) 208 * + 624 ** = 832 cases** | | |
| σₜᵣᵤₑ | Standard deviation of the continuous random variable in the true model | Ranged from 0.25 to 4 by 0.25 |
| β₁ | Beta coefficients of continuous random variable in the true model | log(ranged from 1 to 4 by 0.25) |
| σ_add_ | The standard deviation (std) of the added independent continuous variable for scenario of adding continuous variable or as the std of the underlying continuous variable to simulate categorical variable for scenario of adding an independent categorical variable | std = 1 |
| p_add_ | Probability which was used to simulate binary variable (parameter p) for scenario of adding categorical variable | 0.1, 0.25, 0.5 |
| β₀ | The intercept of the true model | intercept = -1 |
|  |  |  |
| **Univariate true model (categorical variable) 240 # + 720 ## = 960 cases** | | |
| σₜᵣᵤₑ | Standard deviation of the underlying continuous random variable which was used to derive categorical variable in the true model (so one can simulate correlation of categorical variable to other variables using their underlying continuous variable) | Ranged from 0.25 to 4 by 0.25 |
| pₜᵣᵤₑ | Probability was used to simulate categorical/binary variable in the true mdoel (parameter p) | 0.1, 0.25, 0.5 |
| β₁ | Beta coefficients of categorical random variable in the true model | log(1.25, 1.5, 2, 3, 5) |
| p_add_ | Probability which was used to simulate added binary variable (parameter p) for scenario of adding categorical variable | 0.1, 0.25, 0.5 |
| σ_add_ | The standard deviation (std) of the added independent continuous variable for scenario of adding continuous variable or as the std of the underlying continuous variable to simulate categorical variable for scenario of adding an independent categorical variable | std = 1 |
| β₀ | The intercept of the true model | intercept = -1 |
|  | | |
| **True model has two variables (Multivariate simulation)** | | |
| **True model has two continuous variables 4160 cases $** | | |
| σₜᵣᵤₑ₁ | Standard deviation of the first continuous random variable in the true model | Ranged from 0.25 to 4 by 0.25 |
| σₜᵣᵤₑ₂ | Standard deviation of the second continuous random variable in the true model | std = 1 for simplicity |
| ρ | Pearson correlation between two continuous variables in the true model | 0, 0.3, 0.5, 0.8 |
| β₁ | Beta coefficients of the first continuous random variable in the true model | log(ranged from 1 to 4 by 0.25) |
| β₂ | Beta coefficients of the second continuous random variable in the true model | log(1.25, 1.5, 2, 3, 5) |
| p_add_ | Probability which was used to simulate binary variable (parameter p) for scenario of adding categorical variable | 0.1, 0.25, 0.5 |
| σ_add_ | The standard deviation (std) of the added independent continuous variable for scenario of adding continuous variable or as the std of the underlying continuous variable to simulate categorical variable for scenario of adding an independent categorical variable | std = 1 |
| β₀ | The intercept of the true model | intercept = -1 |
|  | | |
| **True model has one continuous variable and one categorical variable 3120 cases ^** | | |
| σₜᵣᵤₑ₁ | Standard deviation of the first continuous random variable in the true model | Ranged from 0.25 to 4 by 1 (for simplicity) |
| pₜᵣᵤₑ | Probability was used to simulate categorical/binary variable from the standard normal distribution in the true model (parameter p) | 0.1, 0.25, 0.5 |
| ρ | Pearson correlation between the continuous variable in the true model and the underlying continuous variable that generates cateogircal variable in the true model | 0, 0.3, 0.5, 0.8 |
| β₁ | Beta coefficients of the continuous random variable in the true model | log(ranged from 1 to 4 by 0.25) |
| β₂ | Beta coefficients of the categorical random variable in the true model | log(1.25, 1.5, 2, 3, 5) |
| p_add_ | Probability which was used to simulate added binary variable (i.e., parameter p) for scenario of adding categorical variable | 0.1, 0.25, 0.5 |
| σ_add_ | The standard deviation (std) of the added independent continuous variable for scenario of adding continuous variable or as the std of the underlying continuous variable to simulate categorical variable for scenario of adding an independent categorical variable | std = 1 |
| β₀ | The intercept of the true model | intercept = -1 |
|  | | |
| @ In the simulation of the univariate analysis, adding either continuous or categorical variable was simulated separately despite that they have the same type of true | | |
| model. While in the multivariate analysis, different types of added variable were simulated at the same time when they have the same true model (as there is no need to simulate the true model repeatedly). This saves computational resources. | | |
| * Continuous variable (True Model) + continuous variable (number of cases: length(σₜᵣᵤₑ) * length(β₁) = 208) | | |
| ** Continuous variable (True Model) + categorical variable (number of cases: length(σₜᵣᵤₑ) * length(β₁) * length(p_add_) = 624) | | |
| # Categorical variable (True Model) + continuous variable (number of cases: length(σₜᵣᵤₑ) *length(pₜᵣᵤₑ) * length(β₁) = 240) | | |
| ## Categorical variable (True Model) + categorical variable (number of cases: length(σₜᵣᵤₑ) * length(pₜᵣᵤₑ) * length(β₁) * length(p_add_) = 720) | | |
| $ Continuous variable + continuous variable (True Model) (number of cases: length(σₜᵣᵤₑ₁) * length(ρ) * length(β₁) * length(β₂) = 4160) | | |
| ^ Continuous variable + categorical variable (True Model) (number of cases: length(σₜᵣᵤₑ₁) * length(pₜᵣᵤₑ)) * length(ρ) * length(β₁) * length(β₂) = 3120) | | |

| **eTable 4: Description of type of models that were compared in the simulation** | |
| --- | --- |
|  | |
| **Type of models** | **Model formula** |
| **True model is a univariate model (Univariate simulation)** | |
| **The outcome generation model that being used to simulate the dataset given a set of simulation parameters** | |
| 1. TrueModel | Logit(p(Y=1)) ~ x1, where x1 could be continuous or categorical in different simulation scenarios |
| **Models that adding one new variable based on the true model** | |
| 2. TrueAddIndModel (AddIndModel); | Logit(p(Y=1)) ~ x1+ x2, where x2 could be continuous or categorical variable in different simulation scenarios, and it (or its underlying continuous variable) is independent to the variable x1 in the true model |
| 3. TrueAdd03Model (Add03Model); | Logit(p(Y=1)) ~ x1 + x2, where x2 could be continuous or categorical variable in different simulation scenarios, and it (or its underlying continuous variable) has correlation of 0.3 to the variable x1 in the true model |
| 4. TrueAdd05Model (Add05Model); | Logit(p(Y=1)) ~ x1+ x2, where x2 could be continuous or categorical variable in different simulation scenarios, and it (or its underlying continuous variable) has correlation of 0.5 to the variable x1 in the true model |
| 5. TrueAdd08Model (Add08Model); | Logit(p(Y=1)) ~ x1 + x2, where x2 could be continuous or categorical variable in different simulation scenarios, and it (or its underlying continuous variable) has correlation of 0.8 to the variable x1 in the true model |
| **True variable was included in the full model with additional variables before the statistical procedure** | |
| 6. AICTrueModel; | stepAIC(Logit(p(Y=1)) ~ x1 * x2_03 * x2_05) with "both" direction, where x2_03 and x2_05 are the variables that have correlation of 0.3 and 0.5 to the variable x1 in the true model. |
| 7. ShrinkTrueModel; | 7.1: Elastic(Logit(p(Y=1)) ~ x1 + x2_03 + x2_05), 7.2: Lasso(Logit(p(Y=1)) ~ x1 + x2_03 + x2_05), 7.3: Ridge(Logit(p(Y=1)) ~ x1 + x2_03 + x2_05), x2_03 and x2_05 are the variables that have correlation of 0.3 and 0.5 to the variable x1 in the true model considering three types of shrinkage. |
| **True variable was excluded but with additional variables in the full model before the statistical procedure** | |
| 8. AICAddModel | stepAIC(Logit(p(Y=1)) ~ x2_03 * x2_05 * x2_08) with "both" direction, where x1 is not collected, and x2_03, x2_05 and x2_08 are the variables that have correlation of 0.3, 0.5 and 0.8 to x1. |
| 9. ShrinkAddModel | 9.1: Elastic(Logit(p(Y=1)) ~ x2_03 + x2_05 + x2_08), 9.2: Lasso(Logit(p(Y=1)) ~ x2_03 + x2_05 + x2_08), 9.3: Ridge(Logit(p(Y=1)) ~ x2_03 + x2_05 + x2_08), where x1 is not collected, and x2_03, x2_05 and x2_08 are the variables that have correlation of 0.3, 0.5 and 0.8 to x1. |
| **A flip fair coin model that does not use any collected information** | |
| 10. FlipCoin | p(Y = 1) = p(Y = 0) = 0.5 |
|  | |
| **True model has two variables (Multivariate simulation)** | |
| **The outcome generation model that being used to simulate the dataset given a set of simulation parameters** | |
| 1. TrueModel | Logit(p(Y=1)) ~ x1 + x2, where x1 is a continuous variable and x2 could be a continuous variable or a categorical variable in different simulation scenarios. x1 and x2 could also have Pearson correlation as 0, 0.3, 0.5, 0.8 in different simulation settings. When x2 is a categorical variable, the correlation is between the variable x1 and the underlying continuous variable that being used to generate the categorical variable x2. |
| **Univariate models which only have one of variables that was in the true model** | |
| 2. UniModelVar1 (Univariate model - Variable1) | Logit(p(Y=1)) ~ x1, where x1 is the same first variable in the true model; |
| 3. UniModelVar2 (Univariate model - Variable2) | Logit(p(Y=1)) ~ x2, where x2 is the same second variable in the true model; |
| **Models that adding one new variable based on the true model (Add one more variable)** | |
| 4. AddIndCont | Logit(p(Y=1)) ~ x1 + x2 + x3, where x3 is an independent continuous variablel; |
| 5. AddCorrVar12 (AddCorrCont) | Logit(p(Y=1)) ~ x1 + x2 + x3, where x3 is a correlated variable to either x1 or x2 used in the true model; |
| 6. AddIndCorrCat (AddCat) | Logit(p(Y=1)) ~ x1 + x2 + x3, where x3 is a categorical/binary variable (i.e., p in 0.1, 0.25, 0.5) which was generated from an underlying continuous variable which was either independent or correlated (i.e., 0.3, 0.5, 0.8 to either x1 or x2 used in the true model); |
| **Models that missing variable1 from the true model but adding one new continuous variable (Missing variable1 - Add one more continuous variable)** | |
| 7. MissVar1AddIndContVar1 (AddIndVar1) | Logit(p(Y=1)) ~ x2 + x3, where x1 is missing and added x3 is independent of x1; |
| 8. MissVar1AddCorrContVar1 (AddCorrVar1) | Logit(p(Y=1)) ~ x2 + x3, where x1 is missing but x3 is correlated to x1 (0.3, 0.5, 0.8); |
| 9. MissVar1AddCorrContVar2 (AddCorrVar2) | Logit(p(Y=1)) ~ x2 + x3, where x1 is missing but x3 is correlated to x2 (0.3, 0.5, 0.8); |
| **Models that missing variable1 from the true model but adding one new categorical/binary variable (Missing variable1 - Add one more categorical variable)** | |
| 10. MissVar1AddIndCorrCatVar1 (AddIndCorrVar1) | Logit(p(Y=1)) ~ x2 + x3, where x3 is a categorical variable (i.e., p in 0.1, 0.25, 0.5) and it has underlying continuous variable which was either independent or correlated (i.e., 0.3, 0.5, 0.8) to excluded variable x1; |
| 11. MissVar1AddCorrCatVar2 (AddCorrVar2) | Logit(p(Y=1)) ~ x2 + x3, where x3 is a categorical variable (i.e., p in 0.1, 0.25, 0.5) and it has underlying continuous variable which was correlated (i.e., 0.3, 0.5, 0.8) to x2; |
| **A flip fair coin model that does not use any collected information** | |
| 12. FlipCoin | p(Y = 1) = p(Y = 0) = 0.5 |

| **eTable 5: Description of type of models that were compared in the case study analysis** | |
| --- | --- |
|  | |
| **Type of models** | **Model formula** |
| **The model only considers linear predictor of variable set 1 (LP1)** | |
| 1. UniVarModel1 (PseudoTrueModel) | Logit(p(Y=1)) ~ LP1, where LP1 is the linear predictor of a pre-trained model using all the variables from variable set 1. This model mimics the true model in univariate simulation. |
| **Add a new variable to the PseudoTrueModel** | |
| 2. TrueAddLowCorrModel | Logit(p(Y=1)) ~ LP1 + VitaminC,  where Pearson correlation between LP1 and VitaminC is 0.02. This model mimics the model which additionally considers an extremely low correlated variable from the true model in the univariate simulation. |
| 3. TrueAdd03Model | Logit(p(Y=1)) ~ LP1 + Glycohemoglobin,  where Pearson correlation between LP1 and Glycohemoglobin is 0.37. This model mimics the model which additionally considers one correlated variable (0.3) from the true model in the univariate simulation. |
| **Linear predictor of variable set 1 (LP1) were considered in the full model with additional variables before conducting the statistical procedure** | |
| 4. AICTrueModel | stepAIC(Logit(p(Y=1)) ~ LP1 + all variables in variable set 2 and variable set 3) with "both" direction, This model mimics the corresponding stepAIC model in the univariate simulation. |
| 5. ShrinkTrueModel | 5.1: Elastic(Logit(p(Y=1)) ~ LP1 + all variables in variable set 2 and variable set 3),  5.2: Lasso(Logit(p(Y=1)) ~ LP1 + all variables in variable set 2 and variable set 3),  5.3: Ridge(Logit(p(Y=1)) ~ LP1 + all variables in variable set 2 and variable set 3), This model mimics the corresponding penalised model in the univariate simulation. |
| **Linear predictor of variable set 1 (LP1) were missed in the full model with additional variables before conducting the statistical procedure** | |
| 6. AICAddModel | stepAIC(Logit(p(Y=1)) ~ all variables in variable set 2 and variable set 3) with "both" direction. This model mimics the corresponding stepAIC model (i.e., true variable was not collected) in the univariate simulation. |
| 7. ShrinkAddModel | 7.1: Elastic(Logit(p(Y=1)) ~ all variables in variable set 2 and variable set 3), 7.2: Lasso(Logit(p(Y=1)) ~ all variables in variable set 2 and variable set 3), 7.3: Ridge(Logit(p(Y=1)) ~ all variables in variable set 2 and variable set 3). This model mimics the corresponding penalised model (i.e., true variable was not collected) in the univariate simulation. |
| **The model considers both of linear predictor of variable set 1 (pseudo casual predictors) and linear predictor of variable set 2 (plausibly related predictors)** | |
| 8. TwoVarModel (PseudoTrueModel) | Logit(p(Y=1)) ~ LP1 + LP2, where LP1 is the linear predictor of a pre-trained model using all the variables from variable set 1, and LP2 is the linear predictor of a pre-trained model using all the variables from variable set 2. This model mimics the two variable model in the multivariate simulation. |
| **Model which only considers linear predictor of variable set 2** | |
| 9. UniVarModel2 | Logit(p(Y=1)) ~ LP2, where LP2 is the linear predictor of a pre-trained model using all the variables from variable set 2. This model mimics the univariate model in the multivariate simulation. |
| **Models that adding one new variable to TwoVarModel (Add one more variable)** | |
| 10. TwoVarAddLowCorrModel | Logit(p(Y=1)) ~ LP1 + LP2 + VitaminC, where the correlation between VitaminC and LP1 is 0.02 and the correlation between VitaminC and LP2 is -0.05. This model mimics the model adding one more variable (low correlated) in the multivariate simulation. |
| 11. TwoVarAddHighCorrModel | Logit(p(Y=1)) ~ LP1 + LP2 + Glycohemoglobin, where the correlation between Glycohemoglobin and LP1 is 0.37 and the correlation between Glycohemoglobin and LP2 is 0.68. This model mimics the model adding one more variable (correlated) in the multivariate simulation. |
| **LP1 is missing but adding one new correlated variable based on TwoVarModel (Missing LP1 - Add one more variable)** | |
| 12. MissAddLowCorrVar1 | Logit(p(Y=1)) ~ LP2 + VitaminC. This model mimics the scenario that LP1 is missing but adding one more variable (low correlated to LP1 and in this case 0.02) to the model as in the multivariate simulation. |
| 13. MissAddHighCorrVar1 | Logit(p(Y=1)) ~ LP2 + Glycohemoglobin. This model mimics the scenario that LP1 is missing but adding one more variable (correlated to LP1 and in this case 0.37) to the model as in the multivariate simulation.; |
| **Models that consider all predictors instead of linear predictors from variable set 1, 2 and 3** | |
| 14. FullModel | Logit(p(Y=1)) ~ all variables in variable set 1, 2 and 3. This model uses all the possible information from collected data. |
| 15. AICFullModel | stepAIC(Logit(p(Y=1)) ~ all variables in variable set 1, 2 and 3) with "both" direction |
| 16. ShrinkFullModel | 16.1: Elastic(Logit(p(Y=1)) ~ all variables in variable set 1, 2 and 3), 16.2: Lasso(Logit(p(Y=1)) ~ all variables in variable set 1, 2 and 3), 16.3: Ridge(Logit(p(Y=1)) ~ all variables in variable set 1, 2 and 3). |
| **The pseudo true model considers predictors in causal variable set 1 and interaction effects of age and sex** | |
| 17. PseudoTrueModelAgeSex | 17. Logit(p(Y=1)) ~ all variables in variable set 1 + age * sex |
| **A flip fair coin model that does not use any collected information** | |
| 18. FlipCoin | p(Y = 1) = p(Y = 0) = 0.5 |

| **eTable 6: Summary of variables with missing values in the case study (patients aged 25-80 years** | | |
| --- | --- | --- |
| **who participated in NHANES 2017-2018*)** | | |
|  | | |
| **Variables with missing values** | **No. patients with missing values** | **Pct. patients with missing values (%)** |
| Ratio of family income to poverty | 615 | 12.7 |
| Median stiffness of kidney | 362 | 7.5 |
| Vitamin E | 357 | 7.4 |
| AST/ALT ratio | 334 | 6.9 |
| C-Reactive Protein | 328 | 6.8 |
| Vitamin C | 304 | 6.3 |
| Cholesterol/HDL ratio | 299 | 6.2 |
| Cotinine | 282 | 5.8 |
| Ferritin | 268 | 5.5 |
| Vitamin A | 264 | 5.4 |
| Total Vitamin D | 258 | 5.3 |
| Mercury | 234 | 4.8 |
| Glycohemoglobin | 227 | 4.7 |
| Whether on hypertension treatment | 175 | 3.6 |
| Those with diabetes or are taking insulin | 163 | 3.4 |
| Diastolic Blood Pressure | 141 | 2.9 |
| Systolic Blood Pressure | 141 | 2.9 |
| Pulse | 140 | 2.9 |
| Albumin | 125 | 2.6 |
| Creatinine | 125 | 2.6 |
| Those with close relative had heart attack | 100 | 2.1 |
| Height | 73 | 1.5 |
| Weight | 73 | 1.5 |
| Sleep Hours | 40 | 0.8 |
| Education | 11 | 0.2 |
| Whether have kidney disease | 9 | 0.2 |
| Marriage | 5 | 0.1 |
|  | | |
| * There were in total 4850 patients in the case study population | | |

| **eTable 7: Comparison of the model performance between the pseudo true model and other proxy models in NHANES 2017-2018 - part II** | | | | | | | | | | |
| --- | --- | --- | --- | --- | --- | --- | --- | --- | --- | --- |
|  | | | | | | | | | | |
|  |  | **Difference of model performance* between the other model and the Pseudo true model** (Mean (95% Range))** | | | | | | | | |
|  |  | **Models only consider LP1 and/or LP2** | |  |  |  |  |  |  |  |
|  |  | **consider LP1 and LP2** | **only consider LP2** | **Add one more variable while considering LP1 and LP2** | | **Missing LP1 but add one new variable while considering LP2** | | **Consider all variables or apply procedures considering all variables** | | |
|  | PseudoTrueModel | TwoVarModel | Univariate model 2 | AddLowCorr | AddHighCorr | AddLowCorr | AddHighCorr | FullModel | AICFullModel | ShrinkFullModel |
| AUC | 0.85 (0.82, 0.88) | 0.00 (-0.00, 0.01) | -0.11 (-0.15, -0.07) | 0.00 (-0.00, 0.01) | 0.01 (-0.00, 0.01) | -0.12 (-0.16, -0.08) | -0.11 (-0.15, -0.07) | 0.01 (-0.00, 0.01) | 0.01 (-0.00, 0.01) | 0.01 (0.00, 0.02) |
| Dxy | 0.70 (0.64, 0.76) | 0.00 (-0.01, 0.01) | -0.22 (-0.30, -0.14) | 0.00 (-0.01, 0.01) | 0.01 (-0.00, 0.02) | -0.24 (-0.32, -0.16) | -0.22 (-0.30, -0.14) | 0.01 (-0.01, 0.03) | 0.01 (-0.01, 0.03) | 0.02 (0.00, 0.03) |
| gIndex | 0.17 (0.16, 0.18) | 0.01 (0.00, 0.01) | -0.08 (-0.10, -0.07) | 0.01 (0.00, 0.01) | 0.01 (0.00, 0.01) | -0.08 (-0.10, -0.07) | -0.08 (-0.10, -0.07) | 0.01 (0.01, 0.02) | 0.01 (0.01, 0.02) | -0.00 (-0.01, 0.00) |
| U | 0.01 (-0.00, 0.02) | -0.00 (-0.00, -0.00) | 0.01 (-0.00, 0.02) | -0.00 (-0.00, -0.00) | -0.00 (-0.00, 0.00) | 0.01 (-0.00, 0.02) | 0.01 (-0.00, 0.02) | -0.00 (-0.01, 0.00) | -0.00 (-0.01, 0.00) | -0.00 (-0.01, 0.01) |
| D | 0.24 (0.19, 0.30) | 0.00 (-0.00, 0.01) | -0.16 (-0.21, -0.11) | 0.01 (-0.00, 0.01) | 0.01 (0.00, 0.02) | -0.17 (-0.22, -0.11) | -0.16 (-0.21, -0.11) | 0.01 (-0.01, 0.03) | 0.01 (-0.01, 0.03) | 0.02 (0.00, 0.03) |
| Q | 0.23 (0.18, 0.28) | 0.01 (-0.00, 0.01) | -0.17 (-0.22, -0.11) | 0.01 (-0.00, 0.02) | 0.01 (0.00, 0.03) | -0.18 (-0.23, -0.12) | -0.17 (-0.22, -0.11) | 0.01 (-0.01, 0.03) | 0.01 (-0.01, 0.03) | 0.02 (0.00, 0.03) |
| OR_caliLarge | 0.81 (0.70, 0.93) | 0.02 (0.01, 0.04) | -0.08 (-0.15, -0.02) | 0.02 (0.01, 0.04) | 0.02 (0.01, 0.03) | -0.09 (-0.15, -0.02) | -0.08 (-0.15, -0.02) | 0.03 (0.01, 0.06) | 0.03 (0.01, 0.06) | 0.02 (-0.01, 0.04) |
| Emax_ab | 0.07 (0.02, 0.14) | -0.01 (-0.02, 0.01) | 0.04 (-0.05, 0.17) | -0.01 (-0.02, 0.00) | -0.00 (-0.02, 0.01) | 0.03 (-0.05, 0.16) | 0.04 (-0.05, 0.17) | -0.01 (-0.04, 0.02) | -0.01 (-0.04, 0.02) | 0.03 (-0.01, 0.06) |
| E_abMean | 0.03 (0.01, 0.05) | -0.00 (-0.01, -0.00) | 0.01 (0.00, 0.02) | -0.00 (-0.01, -0.00) | -0.00 (-0.01, -0.00) | 0.01 (0.00, 0.02) | 0.01 (0.00, 0.02) | -0.00 (-0.01, 0.00) | -0.00 (-0.01, 0.00) | -0.00 (-0.01, 0.01) |
| Emax_01 | 0.07 (0.02, 0.14) | -0.01 (-0.02, 0.01) | 0.04 (-0.05, 0.17) | -0.01 (-0.02, 0.00) | -0.00 (-0.02, 0.01) | 0.03 (-0.05, 0.16) | 0.04 (-0.05, 0.17) | -0.01 (-0.04, 0.02) | -0.01 (-0.04, 0.02) | 0.03 (-0.01, 0.06) |
| E_01Mean | 0.04 (0.01, 0.09) | -0.01 (-0.01, 0.00) | 0.02 (-0.04, 0.10) | -0.01 (-0.01, 0.00) | -0.00 (-0.01, 0.01) | 0.02 (-0.04, 0.09) | 0.02 (-0.04, 0.10) | -0.01 (-0.03, 0.01) | -0.01 (-0.03, 0.01) | 0.02 (-0.00, 0.03) |
| Eavg_LOWESS | 0.03 (0.01, 0.05) | -0.00 (-0.01, 0.00) | 0.03 (0.01, 0.04) | -0.00 (-0.01, 0.00) | -0.00 (-0.01, 0.00) | 0.02 (0.01, 0.04) | 0.03 (0.01, 0.04) | -0.00 (-0.01, 0.00) | -0.00 (-0.01, 0.00) | 0.00 (-0.01, 0.01) |
| ECI_LOWESS | 0.19 (0.04, 0.45) | -0.03 (-0.08, 0.01) | 0.73 (0.31, 1.27) | -0.03 (-0.08, 0.01) | -0.03 (-0.08, 0.02) | 0.73 (0.30, 1.26) | 0.73 (0.31, 1.27) | -0.05 (-0.14, 0.02) | -0.05 (-0.14, 0.02) | 0.02 (-0.09, 0.13) |
| brierScore | 0.11 (0.09, 0.12) | -0.00 (-0.00, 0.00) | 0.03 (0.02, 0.03) | -0.00 (-0.00, 0.00) | -0.00 (-0.00, -0.00) | 0.03 (0.02, 0.03) | 0.03 (0.02, 0.03) | -0.00 (-0.01, 0.00) | -0.00 (-0.01, 0.00) | -0.00 (-0.01, 0.00) |
| R2_Nagelkerke | 0.36 (0.29, 0.43) | 0.01 (-0.00, 0.02) | -0.23 (-0.30, -0.16) | 0.01 (-0.00, 0.02) | 0.02 (0.00, 0.03) | -0.24 (-0.31, -0.16) | -0.23 (-0.30, -0.16) | 0.01 (-0.01, 0.04) | 0.01 (-0.01, 0.04) | 0.02 (0.00, 0.04) |
| R2_McFadden | 0.27 (0.21, 0.32) | 0.01 (-0.00, 0.01) | -0.18 (-0.23, -0.12) | 0.01 (-0.00, 0.02) | 0.01 (0.00, 0.03) | -0.18 (-0.24, -0.13) | -0.18 (-0.23, -0.12) | 0.01 (-0.01, 0.03) | 0.01 (-0.01, 0.03) | 0.02 (0.00, 0.04) |
| R2_McFaddenAdj | 0.26 (0.21, 0.32) | 0.00 (-0.01, 0.01) | -0.18 (-0.23, -0.12) | 0.00 (-0.01, 0.01) | 0.01 (-0.00, 0.02) | -0.19 (-0.24, -0.13) | -0.18 (-0.24, -0.13) | -0.07 (-0.09, -0.05) | -0.07 (-0.09, -0.05) | -0.06 (-0.08, -0.04) |
| R2_CoxSnell | 0.21 (0.17, 0.26) | 0.00 (-0.00, 0.01) | -0.14 (-0.18, -0.09) | 0.00 (-0.00, 0.01) | 0.01 (0.00, 0.02) | -0.14 (-0.18, -0.10) | -0.14 (-0.18, -0.09) | 0.01 (-0.01, 0.02) | 0.01 (-0.01, 0.02) | 0.01 (0.00, 0.03) |
| R2_AldrichNelson | 0.19 (0.16, 0.23) | 0.00 (-0.00, 0.01) | -0.12 (-0.16, -0.08) | 0.00 (-0.00, 0.01) | 0.01 (0.00, 0.01) | -0.13 (-0.16, -0.09) | -0.12 (-0.16, -0.08) | 0.01 (-0.01, 0.02) | 0.01 (-0.01, 0.02) | 0.01 (0.00, 0.02) |
| R2_VeallZimmermann | 0.41 (0.34, 0.48) | 0.01 (-0.00, 0.02) | -0.26 (-0.33, -0.17) | 0.01 (-0.00, 0.02) | 0.02 (0.00, 0.03) | -0.26 (-0.34, -0.18) | -0.26 (-0.33, -0.17) | 0.01 (-0.02, 0.04) | 0.01 (-0.02, 0.04) | 0.02 (0.00, 0.04) |
| R2_Efron | 0.24 (0.18, 0.29) | 0.01 (-0.00, 0.02) | -0.18 (-0.24, -0.12) | 0.01 (-0.00, 0.02) | 0.02 (0.00, 0.03) | -0.19 (-0.24, -0.12) | -0.18 (-0.24, -0.12) | 0.02 (-0.00, 0.04) | 0.02 (-0.00, 0.04) | 0.02 (-0.00, 0.04) |
| R2_McKelveyZavoina | 0.46 (0.44, 0.47) | 0.01 (0.00, 0.01) | -0.32 (-0.35, -0.30) | 0.01 (0.00, 0.01) | 0.01 (0.00, 0.01) | -0.32 (-0.35, -0.30) | -0.32 (-0.35, -0.30) | 0.03 (0.02, 0.04) | 0.03 (0.02, 0.04) | -0.05 (-0.06, -0.04) |
| R2_Tjur | 0.23 (0.19, 0.26) | 0.01 (0.01, 0.02) | -0.16 (-0.19, -0.13) | 0.01 (0.01, 0.02) | 0.01 (0.01, 0.02) | -0.16 (-0.19, -0.13) | -0.16 (-0.19, -0.13) | 0.03 (0.02, 0.04) | 0.03 (0.02, 0.04) | 0.00 (-0.01, 0.02) |
| GlobalShrinkageFactor | 0.99 (0.99, 0.99) | -0.00 (-0.01, -0.00) | -0.02 (-0.03, -0.01) | -0.01 (-0.01, -0.01) | -0.01 (-0.01, -0.01) | -0.04 (-0.06, -0.02) | -0.03 (-0.06, -0.02) | -0.15 (-0.18, -0.12) | -0.14 (-0.18, -0.12) | -0.14 (-0.18, -0.11) |
| IDI_diff1 | 0.23 (0.19, 0.26) | 0.01 (0.01, 0.02) | -0.16 (-0.19, -0.13) | 0.01 (0.01, 0.02) | 0.01 (0.01, 0.02) | -0.16 (-0.19, -0.13) | -0.16 (-0.19, -0.13) | 0.03 (0.02, 0.04) | 0.03 (0.02, 0.04) | 0.00 (-0.01, 0.02) |
|  | | | | | | | | | | |
| * The difference of performance measure = performance measure of each model - performance measure of the pseudo true model. | | | | | | | | | | |
| ** Pseudo true model was assumed as a model considers linear predictor of causal predictor set 1 (LP1). | | | | | | | | | | |

| **eTable 8: Comparison of the model performance between the multivariate pseudo true model and other proxy models in NHANES 2017-2018 - part I** | | | | | | | | | | | |
| --- | --- | --- | --- | --- | --- | --- | --- | --- | --- | --- | --- |
|  | | | | | | | | | | | |
|  |  | **Difference of model performance* between the other model and the Pseudo true model** (Mean (95% Range))** | | | | | | | | | |
|  |  | **Flip a fair coin** | **only consider LP1** | **only consider LP2** | **Add one more variable while considering LP1 and LP2** | | **Missing LP1 but add one new variable while considering LP2** | | **Consider all variables or apply procedures considering all variables** | | |
|  | PseudoTrueModel | FlipCoin | Univariate model 1 | Univariate model 2 | AddLowCorr | AddHighCorr | AddLowCorr | AddHighCorr | FullModel | AICFullModel | ShrinkFullModel |
| AUC | 0.85 (0.82, 0.88) | -0.35 (-0.38, -0.32) | -0.00 (-0.01, 0.00) | -0.11 (-0.15, -0.07) | 0.00 (0.00, 0.00) | 0.00 (0.00, 0.01) | -0.12 (-0.16, -0.08) | -0.11 (-0.15, -0.07) | 0.00 (-0.00, 0.01) | 0.00 (-0.00, 0.01) | 0.01 (0.00, 0.02) |
| Dxy | 0.70 (0.64, 0.76) | -0.70 (-0.76, -0.64) | -0.00 (-0.01, 0.01) | -0.22 (-0.30, -0.15) | 0.00 (0.00, 0.00) | 0.01 (0.00, 0.01) | -0.24 (-0.32, -0.17) | -0.22 (-0.30, -0.15) | 0.01 (-0.01, 0.02) | 0.01 (-0.01, 0.02) | 0.02 (0.00, 0.03) |
| gIndex | 0.18 (0.16, 0.19) | -0.18 (-0.19, -0.16) | -0.01 (-0.01, -0.00) | -0.09 (-0.10, -0.08) | 0.00 (-0.00, 0.00) | -0.00 (-0.00, 0.00) | -0.09 (-0.10, -0.08) | -0.09 (-0.10, -0.08) | 0.01 (0.00, 0.01) | 0.01 (0.00, 0.01) | -0.01 (-0.01, -0.01) |
| U | 0.01 (-0.00, 0.02) | 0.47 (0.38, 0.56) | 0.00 (0.00, 0.00) | 0.01 (0.00, 0.02) | -0.00 (-0.00, 0.00) | 0.00 (-0.00, 0.00) | 0.01 (0.00, 0.02) | 0.01 (0.00, 0.02) | 0.00 (-0.00, 0.00) | 0.00 (-0.00, 0.00) | 0.00 (-0.00, 0.01) |
| D | 0.25 (0.19, 0.30) | -0.25 (-0.30, -0.19) | -0.00 (-0.01, 0.00) | -0.17 (-0.22, -0.12) | 0.00 (0.00, 0.00) | 0.01 (0.00, 0.01) | -0.17 (-0.22, -0.12) | -0.17 (-0.22, -0.12) | 0.01 (-0.01, 0.02) | 0.01 (-0.01, 0.02) | 0.01 (-0.00, 0.02) |
| Q | 0.24 (0.19, 0.29) | -0.72 (-0.79, -0.64) | -0.01 (-0.01, 0.00) | -0.18 (-0.23, -0.12) | 0.00 (0.00, 0.00) | 0.01 (0.00, 0.01) | -0.18 (-0.23, -0.13) | -0.18 (-0.23, -0.12) | 0.01 (-0.01, 0.02) | 0.01 (-0.01, 0.02) | 0.01 (-0.00, 0.02) |
| OR_caliLarge | 0.84 (0.72, 0.96) | 4.14 (3.43, 4.95) | -0.02 (-0.04, -0.01) | -0.11 (-0.17, -0.05) | 0.00 (0.00, 0.00) | -0.00 (-0.01, 0.01) | -0.11 (-0.17, -0.05) | -0.11 (-0.17, -0.05) | 0.01 (-0.01, 0.03) | 0.01 (-0.01, 0.03) | -0.00 (-0.03, 0.01) |
| Emax_ab | 0.06 (0.02, 0.13) | 0.27 (0.19, 0.33) | 0.01 (-0.01, 0.02) | 0.05 (-0.03, 0.17) | 0.00 (-0.00, 0.00) | 0.00 (-0.01, 0.02) | 0.04 (-0.03, 0.16) | 0.05 (-0.03, 0.17) | -0.01 (-0.03, 0.02) | -0.01 (-0.03, 0.02) | 0.04 (-0.01, 0.07) |
| E_abMean | 0.02 (0.01, 0.04) | NA | 0.00 (0.00, 0.01) | 0.02 (0.01, 0.03) | -0.00 (-0.00, 0.00) | 0.00 (-0.00, 0.00) | 0.02 (0.01, 0.03) | 0.02 (0.01, 0.03) | -0.00 (-0.00, 0.00) | -0.00 (-0.00, 0.00) | 0.00 (-0.00, 0.01) |
| Emax_01 | 0.06 (0.02, 0.13) | 0.27 (0.19, 0.33) | 0.01 (-0.01, 0.02) | 0.05 (-0.03, 0.18) | 0.00 (-0.00, 0.00) | 0.00 (-0.01, 0.02) | 0.04 (-0.03, 0.16) | 0.05 (-0.03, 0.18) | -0.01 (-0.03, 0.02) | -0.01 (-0.03, 0.02) | 0.04 (-0.01, 0.07) |
| E_01Mean | 0.04 (0.01, 0.08) | NA | 0.01 (-0.00, 0.01) | 0.03 (-0.02, 0.10) | 0.00 (-0.00, 0.00) | 0.00 (-0.00, 0.01) | 0.02 (-0.03, 0.10) | 0.03 (-0.02, 0.10) | -0.00 (-0.02, 0.01) | -0.00 (-0.02, 0.01) | 0.02 (-0.00, 0.04) |
| Eavg_LOWESS | 0.03 (0.01, 0.05) | 0.30 (0.26, 0.34) | 0.00 (-0.00, 0.01) | 0.03 (0.01, 0.04) | -0.00 (-0.00, 0.00) | -0.00 (-0.00, 0.00) | 0.03 (0.01, 0.04) | 0.03 (0.01, 0.04) | -0.00 (-0.01, 0.00) | -0.00 (-0.01, 0.00) | 0.00 (-0.01, 0.01) |
| ECI_LOWESS | 0.16 (0.03, 0.39) | 10.86 (9.03, 12.53) | 0.03 (-0.01, 0.08) | 0.77 (0.34, 1.31) | -0.00 (-0.01, 0.00) | 0.00 (-0.04, 0.04) | 0.76 (0.33, 1.29) | 0.77 (0.34, 1.31) | -0.02 (-0.10, 0.04) | -0.02 (-0.10, 0.04) | 0.05 (-0.08, 0.17) |
| brierScore | 0.11 (0.09, 0.12) | 0.14 (0.13, 0.16) | 0.00 (-0.00, 0.00) | 0.03 (0.02, 0.04) | -0.00 (-0.00, -0.00) | -0.00 (-0.00, 0.00) | 0.03 (0.02, 0.04) | 0.03 (0.02, 0.04) | -0.00 (-0.00, 0.00) | -0.00 (-0.00, 0.00) | -0.00 (-0.00, 0.00) |
| R2_Nagelkerke | 0.37 (0.30, 0.43) | -0.37 (-0.43, -0.30) | -0.01 (-0.02, 0.00) | -0.24 (-0.30, -0.17) | 0.00 (0.00, 0.00) | 0.01 (0.00, 0.02) | -0.25 (-0.31, -0.18) | -0.24 (-0.30, -0.17) | 0.01 (-0.02, 0.03) | 0.01 (-0.02, 0.03) | 0.01 (-0.00, 0.03) |
| R2_McFadden | 0.27 (0.22, 0.33) | -0.27 (-0.33, -0.22) | -0.01 (-0.01, 0.00) | -0.18 (-0.23, -0.13) | 0.00 (0.00, 0.00) | 0.01 (0.00, 0.01) | -0.19 (-0.24, -0.14) | -0.18 (-0.23, -0.13) | 0.01 (-0.01, 0.02) | 0.01 (-0.01, 0.02) | 0.01 (-0.00, 0.03) |
| R2_McFaddenAdj | 0.27 (0.21, 0.32) | -0.27 (-0.32, -0.21) | -0.00 (-0.01, 0.01) | -0.18 (-0.23, -0.13) | -0.00 (-0.00, -0.00) | 0.01 (-0.00, 0.01) | -0.19 (-0.24, -0.14) | -0.18 (-0.23, -0.13) | -0.07 (-0.10, -0.05) | -0.07 (-0.09, -0.05) | -0.07 (-0.08, -0.05) |
| R2_CoxSnell | 0.22 (0.18, 0.26) | -0.22 (-0.26, -0.18) | -0.00 (-0.01, 0.00) | -0.14 (-0.18, -0.10) | 0.00 (0.00, 0.00) | 0.01 (0.00, 0.01) | -0.15 (-0.18, -0.10) | -0.14 (-0.18, -0.10) | 0.00 (-0.01, 0.02) | 0.00 (-0.01, 0.02) | 0.01 (-0.00, 0.02) |
| R2_AldrichNelson | 0.20 (0.16, 0.23) | -0.20 (-0.23, -0.16) | -0.00 (-0.01, 0.00) | -0.12 (-0.16, -0.09) | 0.00 (0.00, 0.00) | 0.00 (0.00, 0.01) | -0.13 (-0.16, -0.09) | -0.12 (-0.16, -0.09) | 0.00 (-0.01, 0.01) | 0.00 (-0.01, 0.01) | 0.01 (-0.00, 0.02) |
| R2_VeallZimmermann | 0.42 (0.35, 0.48) | -0.42 (-0.48, -0.35) | -0.01 (-0.02, 0.00) | -0.26 (-0.33, -0.19) | 0.00 (0.00, 0.00) | 0.01 (0.00, 0.02) | -0.27 (-0.33, -0.20) | -0.26 (-0.33, -0.19) | 0.01 (-0.02, 0.03) | 0.01 (-0.02, 0.03) | 0.01 (-0.00, 0.03) |
| R2_Efron | 0.25 (0.19, 0.30) | -1.04 (-1.25, -0.85) | -0.01 (-0.02, 0.00) | -0.19 (-0.24, -0.13) | 0.00 (0.00, 0.00) | 0.01 (-0.00, 0.02) | -0.19 (-0.25, -0.13) | -0.19 (-0.24, -0.13) | 0.01 (-0.01, 0.03) | 0.01 (-0.01, 0.03) | 0.01 (-0.01, 0.03) |
| R2_McKelveyZavoina | 0.46 (0.45, 0.48) | -0.46 (-0.48, -0.45) | -0.01 (-0.01, -0.00) | -0.33 (-0.35, -0.31) | 0.00 (-0.00, 0.00) | 0.00 (-0.00, 0.00) | -0.33 (-0.35, -0.30) | -0.33 (-0.35, -0.31) | 0.02 (0.02, 0.03) | 0.02 (0.02, 0.03) | -0.05 (-0.06, -0.04) |
| R2_Tjur | 0.24 (0.20, 0.27) | -0.24 (-0.27, -0.20) | -0.01 (-0.02, -0.01) | -0.17 (-0.20, -0.14) | 0.00 (0.00, 0.00) | 0.00 (-0.00, 0.01) | -0.17 (-0.20, -0.14) | -0.17 (-0.20, -0.14) | 0.02 (0.00, 0.03) | 0.02 (0.00, 0.03) | -0.01 (-0.02, 0.01) |
| GlobalShrinkageFactor | 0.99 (0.98, 0.99) | NA | 0.00 (0.00, 0.00) | -0.02 (-0.03, -0.01) | -0.00 (-0.01, -0.00) | -0.00 (-0.00, -0.00) | -0.03 (-0.06, -0.01) | -0.03 (-0.05, -0.01) | -0.14 (-0.18, -0.12) | -0.14 (-0.17, -0.11) | -0.14 (-0.17, -0.11) |
| IDI_diff1 | 0.24 (0.20, 0.27) | -0.24 (-0.27, -0.20) | -0.01 (-0.02, -0.01) | -0.17 (-0.20, -0.14) | 0.00 (0.00, 0.00) | 0.00 (-0.00, 0.01) | -0.17 (-0.20, -0.14) | -0.17 (-0.20, -0.14) | 0.02 (0.00, 0.03) | 0.02 (0.00, 0.03) | -0.01 (-0.02, 0.01) |
|  | | | | | | | | | | | |
| * The difference of performance measure = performance measure of each model - performance measure of the pseudo true model. | | | | | | | | | | | |
| ** Pseudo true model was assumed as a model considers linear predictor of variable set 1 (LP1) and variable set 2 (LP2) | | | | | | | | | | | |

| **eTable 9: Comparison of the model performance between the multivariate pseudo true model and other proxy models in NHANES 2017-2018 - part II** | | | | | | | |
| --- | --- | --- | --- | --- | --- | --- | --- |
|  | | | | | | | |
|  |  | **Difference of model performance* between the other model and the pseudo true model** (Mean (95% Range))** | | | | | |
|  |  | **Add one more variable while only considering LP1** | | **LP1 was included before the procedure** | | **LP1 was excluded before the procedure** | |
|  | PseudoTrueModel | AddLowCorrModel | Add03Model | AICTrueModel | ShrinkTrueModel | AICAddModel | ShrinkAddModel |
| AUC | 0.85 (0.82, 0.88) | 0.00 (-0.00, 0.01) | -0.00 (-0.01, 0.00) | 0.01 (-0.00, 0.01) | 0.00 (-0.00, 0.01) | -0.07 (-0.10, -0.04) | -0.06 (-0.09, -0.03) |
| Dxy | 0.70 (0.64, 0.76) | 0.00 (-0.01, 0.01) | -0.00 (-0.01, 0.01) | 0.01 (-0.00, 0.03) | 0.01 (-0.00, 0.03) | -0.14 (-0.20, -0.08) | -0.12 (-0.18, -0.06) |
| gIndex | 0.18 (0.16, 0.19) | -0.00 (-0.01, -0.00) | -0.01 (-0.01, -0.00) | 0.01 (0.00, 0.01) | -0.01 (-0.02, -0.01) | -0.06 (-0.07, -0.05) | -0.08 (-0.09, -0.07) |
| U | 0.01 (-0.00, 0.02) | 0.00 (-0.00, 0.00) | 0.00 (0.00, 0.00) | -0.00 (-0.01, 0.00) | 0.00 (-0.00, 0.01) | 0.01 (0.00, 0.02) | 0.02 (0.00, 0.04) |
| D | 0.25 (0.19, 0.30) | -0.00 (-0.01, 0.01) | -0.00 (-0.01, 0.00) | 0.01 (-0.01, 0.02) | 0.00 (-0.01, 0.02) | -0.12 (-0.16, -0.07) | -0.11 (-0.16, -0.07) |
| Q | 0.24 (0.19, 0.29) | -0.00 (-0.01, 0.00) | -0.01 (-0.01, 0.00) | 0.01 (-0.01, 0.03) | 0.00 (-0.01, 0.02) | -0.13 (-0.17, -0.08) | -0.13 (-0.18, -0.08) |
| OR_caliLarge | 0.84 (0.72, 0.96) | -0.02 (-0.03, -0.01) | -0.02 (-0.04, -0.01) | 0.03 (0.01, 0.05) | -0.02 (-0.04, -0.01) | -0.09 (-0.15, -0.03) | -0.15 (-0.20, -0.09) |
| Emax_ab | 0.06 (0.02, 0.13) | 0.01 (-0.01, 0.02) | 0.01 (-0.01, 0.02) | -0.01 (-0.04, 0.02) | 0.05 (-0.01, 0.08) | 0.04 (-0.03, 0.12) | 0.12 (0.02, 0.21) |
| E_abMean | 0.02 (0.01, 0.04) | 0.00 (0.00, 0.00) | 0.00 (0.00, 0.01) | -0.00 (-0.01, 0.00) | 0.00 (-0.00, 0.01) | 0.01 (0.00, 0.02) | 0.02 (0.01, 0.03) |
| Emax_01 | 0.06 (0.02, 0.13) | 0.01 (-0.01, 0.02) | 0.01 (-0.01, 0.02) | -0.01 (-0.04, 0.02) | 0.05 (-0.01, 0.08) | 0.04 (-0.03, 0.12) | 0.12 (0.02, 0.22) |
| E_01Mean | 0.04 (0.01, 0.08) | 0.01 (-0.00, 0.01) | 0.01 (-0.00, 0.01) | -0.01 (-0.02, 0.01) | 0.03 (-0.00, 0.05) | 0.02 (-0.02, 0.08) | 0.07 (0.02, 0.13) |
| Eavg_LOWESS | 0.03 (0.01, 0.05) | 0.00 (-0.00, 0.01) | 0.00 (-0.00, 0.01) | -0.00 (-0.01, 0.00) | 0.01 (-0.01, 0.01) | 0.03 (0.01, 0.04) | 0.04 (0.02, 0.05) |
| ECI_LOWESS | 0.16 (0.03, 0.39) | 0.03 (-0.02, 0.07) | 0.03 (-0.01, 0.08) | -0.04 (-0.12, 0.02) | 0.08 (-0.05, 0.23) | 0.35 (0.10, 0.68) | 0.63 (0.30, 1.06) |
| brierScore | 0.11 (0.09, 0.12) | 0.00 (-0.00, 0.00) | 0.00 (-0.00, 0.00) | -0.00 (-0.00, 0.00) | 0.00 (-0.00, 0.00) | 0.02 (0.01, 0.03) | 0.02 (0.01, 0.03) |
| R2_Nagelkerke | 0.37 (0.30, 0.43) | -0.00 (-0.01, 0.01) | -0.01 (-0.02, 0.00) | 0.01 (-0.01, 0.03) | 0.01 (-0.01, 0.03) | -0.17 (-0.23, -0.10) | -0.15 (-0.22, -0.09) |
| R2_McFadden | 0.27 (0.22, 0.33) | -0.00 (-0.01, 0.01) | -0.01 (-0.01, 0.00) | 0.01 (-0.01, 0.03) | 0.01 (-0.01, 0.02) | -0.13 (-0.18, -0.08) | -0.12 (-0.17, -0.07) |
| R2_McFaddenAdj | 0.27 (0.21, 0.32) | -0.00 (-0.01, 0.01) | -0.01 (-0.01, 0.00) | -0.04 (-0.05, -0.02) | -0.05 (-0.07, -0.03) | -0.18 (-0.22, -0.13) | -0.18 (-0.23, -0.13) |
| R2_CoxSnell | 0.22 (0.18, 0.26) | -0.00 (-0.01, 0.00) | -0.00 (-0.01, 0.00) | 0.01 (-0.01, 0.02) | 0.00 (-0.01, 0.02) | -0.10 (-0.14, -0.06) | -0.09 (-0.13, -0.06) |
| R2_AldrichNelson | 0.20 (0.16, 0.23) | -0.00 (-0.01, 0.00) | -0.00 (-0.01, 0.00) | 0.00 (-0.01, 0.01) | 0.00 (-0.00, 0.01) | -0.08 (-0.12, -0.05) | -0.08 (-0.11, -0.05) |
| R2_VeallZimmermann | 0.42 (0.35, 0.48) | -0.00 (-0.01, 0.01) | -0.01 (-0.02, 0.00) | 0.01 (-0.01, 0.03) | 0.01 (-0.01, 0.03) | -0.18 (-0.24, -0.11) | -0.17 (-0.23, -0.10) |
| R2_Efron | 0.25 (0.19, 0.30) | -0.01 (-0.02, 0.01) | -0.01 (-0.02, 0.00) | 0.01 (-0.01, 0.03) | 0.00 (-0.02, 0.02) | -0.14 (-0.19, -0.08) | -0.15 (-0.20, -0.10) |
| R2_McKelveyZavoina | 0.46 (0.45, 0.48) | -0.00 (-0.01, -0.00) | -0.01 (-0.01, -0.00) | 0.02 (0.01, 0.03) | -0.06 (-0.08, -0.06) | -0.24 (-0.26, -0.22) | -0.28 (-0.30, -0.26) |
| R2_Tjur | 0.24 (0.20, 0.27) | -0.01 (-0.01, -0.00) | -0.01 (-0.02, -0.01) | 0.02 (0.01, 0.03) | -0.02 (-0.03, -0.01) | -0.12 (-0.15, -0.10) | -0.14 (-0.17, -0.12) |
| GlobalShrinkageFactor | 0.99 (0.98, 0.99) | -0.00 (-0.00, 0.00) | -0.00 (-0.00, 0.00) | -0.08 (-0.10, -0.06) | -0.10 (-0.13, -0.08) | -0.17 (-0.23, -0.12) | -0.19 (-0.23, -0.14) |
| IDI_diff1 | 0.24 (0.20, 0.27) | -0.01 (-0.01, -0.00) | -0.01 (-0.02, -0.01) | 0.02 (0.01, 0.03) | -0.02 (-0.03, -0.01) | -0.12 (-0.15, -0.10) | -0.14 (-0.17, -0.12) |
|  | | | | | | | |
| * The difference of performance measure = performance measure of each model - performance measure of the pseudo true model. | | | | | | | |
| ** Pseudo true model was assumed as a model considers linear predictor of variable set 1 (LP1) and variable set 2 (LP2). | | | | | | | |

| **eTable 10: Comparison of the model performance between the pseudo true model (consider interaction effects of age and sex) and other proxy models in NHANES 2017-2018 - part I** | | | | | | | | | |
| --- | --- | --- | --- | --- | --- | --- | --- | --- | --- |
|  | | | | | | | | | |
|  |  | **Difference of model performance* between the other model and the pseudo true model** (Mean (95% Range))** | | | | | | | |
|  |  | **Flip a fair coin** | **only consider LP1** | **Add one more variable while considering LP1** | | **LP1 was included before the procedure** | | **LP1 was excluded before the procedure** | |
|  | PseudoTrueModel | FlipCoin | Univariate model 1 | AddLowCorrModel | Add03Model | AICTrueModel | ShrinkTrueModel | AICAddModel | ShrinkAddModel |
| AUC | 0.85 (0.82, 0.88) | -0.35 (-0.38, -0.32) | -0.00 (-0.00, 0.00) | 0.00 (-0.00, 0.00) | -0.00 (-0.00, 0.00) | 0.01 (-0.00, 0.02) | 0.00 (-0.00, 0.02) | -0.07 (-0.10, -0.04) | -0.06 (-0.09, -0.03) |
| Dxy | 0.70 (0.65, 0.76) | -0.70 (-0.76, -0.65) | -0.00 (-0.01, 0.00) | 0.00 (-0.00, 0.01) | -0.00 (-0.01, 0.00) | 0.01 (-0.01, 0.03) | 0.01 (-0.00, 0.03) | -0.14 (-0.21, -0.07) | -0.12 (-0.18, -0.06) |
| gIndex | 0.17 (0.16, 0.18) | -0.17 (-0.18, -0.16) | -0.00 (-0.00, -0.00) | -0.00 (-0.00, 0.00) | -0.00 (-0.00, -0.00) | 0.01 (0.01, 0.02) | -0.01 (-0.01, -0.01) | -0.06 (-0.07, -0.04) | -0.07 (-0.09, -0.06) |
| U | 0.01 (-0.00, 0.02) | 0.47 (0.38, 0.56) | 0.00 (0.00, 0.00) | 0.00 (-0.00, 0.00) | 0.00 (0.00, 0.00) | -0.00 (-0.01, 0.00) | 0.00 (-0.00, 0.01) | 0.01 (-0.00, 0.02) | 0.02 (0.00, 0.04) |
| D | 0.24 (0.19, 0.30) | -0.24 (-0.30, -0.19) | -0.00 (-0.00, 0.00) | 0.00 (-0.00, 0.00) | -0.00 (-0.00, 0.00) | 0.01 (-0.01, 0.03) | 0.01 (-0.00, 0.03) | -0.12 (-0.16, -0.06) | -0.11 (-0.16, -0.06) |
| Q | 0.23 (0.18, 0.28) | -0.71 (-0.79, -0.64) | -0.00 (-0.00, 0.00) | 0.00 (-0.00, 0.00) | -0.00 (-0.00, 0.00) | 0.01 (-0.01, 0.04) | 0.01 (-0.01, 0.03) | -0.12 (-0.17, -0.07) | -0.12 (-0.17, -0.07) |
| OR_caliLarge | 0.82 (0.70, 0.94) | 4.16 (3.44, 4.96) | -0.01 (-0.01, -0.00) | -0.00 (-0.01, 0.00) | -0.01 (-0.01, -0.00) | 0.05 (0.03, 0.07) | -0.01 (-0.02, 0.01) | -0.07 (-0.14, -0.01) | -0.13 (-0.19, -0.07) |
| Emax_ab | 0.07 (0.02, 0.14) | 0.26 (0.18, 0.33) | 0.00 (-0.00, 0.01) | 0.00 (-0.00, 0.01) | 0.00 (-0.00, 0.01) | -0.02 (-0.05, 0.02) | 0.04 (-0.00, 0.08) | 0.03 (-0.04, 0.12) | 0.11 (0.02, 0.21) |
| E_abMean | 0.03 (0.01, 0.05) | NA | 0.00 (0.00, 0.00) | 0.00 (-0.00, 0.00) | 0.00 (0.00, 0.00) | -0.01 (-0.01, 0.00) | 0.00 (-0.00, 0.01) | 0.01 (0.00, 0.02) | 0.02 (0.01, 0.03) |
| Emax_01 | 0.07 (0.02, 0.14) | 0.26 (0.18, 0.33) | 0.00 (-0.00, 0.01) | 0.00 (-0.00, 0.01) | 0.00 (-0.00, 0.01) | -0.02 (-0.05, 0.02) | 0.04 (-0.00, 0.08) | 0.03 (-0.04, 0.12) | 0.11 (0.02, 0.22) |
| E_01Mean | 0.04 (0.01, 0.09) | NA | 0.00 (-0.00, 0.00) | 0.00 (-0.00, 0.00) | 0.00 (-0.00, 0.00) | -0.01 (-0.03, 0.01) | 0.02 (-0.00, 0.04) | 0.02 (-0.03, 0.07) | 0.07 (0.01, 0.13) |
| Eavg_LOWESS | 0.03 (0.01, 0.05) | 0.30 (0.26, 0.34) | 0.00 (-0.00, 0.00) | -0.00 (-0.00, 0.00) | 0.00 (-0.00, 0.00) | -0.01 (-0.01, 0.00) | 0.00 (-0.01, 0.01) | 0.02 (0.01, 0.04) | 0.03 (0.02, 0.05) |
| ECI_LOWESS | 0.19 (0.04, 0.45) | 10.82 (8.98, 12.51) | -0.01 (-0.03, 0.02) | -0.01 (-0.04, 0.01) | -0.00 (-0.03, 0.02) | -0.07 (-0.18, 0.01) | 0.05 (-0.09, 0.18) | 0.31 (0.07, 0.63) | 0.59 (0.26, 1.03) |
| brierScore | 0.11 (0.09, 0.12) | 0.14 (0.13, 0.16) | 0.00 (-0.00, 0.00) | -0.00 (-0.00, 0.00) | 0.00 (-0.00, 0.00) | -0.00 (-0.01, 0.00) | -0.00 (-0.00, 0.00) | 0.02 (0.01, 0.03) | 0.02 (0.01, 0.03) |
| R2_Nagelkerke | 0.36 (0.29, 0.43) | -0.36 (-0.43, -0.29) | -0.00 (-0.00, 0.00) | 0.00 (-0.00, 0.01) | -0.00 (-0.00, 0.00) | 0.01 (-0.01, 0.04) | 0.01 (-0.01, 0.04) | -0.16 (-0.23, -0.09) | -0.15 (-0.22, -0.08) |
| R2_McFadden | 0.27 (0.22, 0.32) | -0.27 (-0.32, -0.22) | -0.00 (-0.00, 0.00) | 0.00 (-0.00, 0.01) | -0.00 (-0.00, 0.00) | 0.01 (-0.01, 0.03) | 0.01 (-0.01, 0.03) | -0.13 (-0.18, -0.07) | -0.12 (-0.17, -0.06) |
| R2_McFaddenAdj | 0.23 (0.18, 0.28) | -0.23 (-0.28, -0.18) | 0.03 (0.03, 0.04) | 0.03 (0.03, 0.04) | 0.03 (0.03, 0.03) | 0.00 (-0.02, 0.02) | -0.02 (-0.03, 0.01) | -0.14 (-0.19, -0.09) | -0.14 (-0.20, -0.09) |
| R2_CoxSnell | 0.22 (0.17, 0.26) | -0.22 (-0.26, -0.17) | -0.00 (-0.00, 0.00) | 0.00 (-0.00, 0.00) | -0.00 (-0.00, 0.00) | 0.01 (-0.01, 0.02) | 0.01 (-0.00, 0.02) | -0.10 (-0.14, -0.05) | -0.09 (-0.13, -0.05) |
| R2_AldrichNelson | 0.20 (0.16, 0.23) | -0.20 (-0.23, -0.16) | -0.00 (-0.00, 0.00) | 0.00 (-0.00, 0.00) | -0.00 (-0.00, 0.00) | 0.01 (-0.01, 0.02) | 0.01 (-0.00, 0.02) | -0.08 (-0.12, -0.05) | -0.08 (-0.11, -0.04) |
| R2_VeallZimmermann | 0.41 (0.34, 0.48) | -0.41 (-0.48, -0.34) | -0.00 (-0.01, 0.00) | 0.00 (-0.00, 0.01) | -0.00 (-0.01, 0.00) | 0.02 (-0.01, 0.04) | 0.01 (-0.01, 0.04) | -0.17 (-0.25, -0.10) | -0.16 (-0.23, -0.09) |
| R2_Efron | 0.24 (0.18, 0.29) | -1.03 (-1.25, -0.84) | -0.00 (-0.00, 0.00) | 0.00 (-0.00, 0.01) | -0.00 (-0.00, 0.00) | 0.02 (-0.00, 0.05) | 0.01 (-0.01, 0.04) | -0.13 (-0.19, -0.07) | -0.14 (-0.19, -0.08) |
| R2_McKelveyZavoina | 0.45 (0.44, 0.47) | -0.45 (-0.47, -0.44) | 0.00 (0.00, 0.01) | 0.01 (0.00, 0.01) | 0.00 (0.00, 0.01) | 0.03 (0.02, 0.04) | -0.05 (-0.07, -0.05) | -0.23 (-0.26, -0.21) | -0.27 (-0.30, -0.25) |
| R2_Tjur | 0.23 (0.20, 0.26) | -0.23 (-0.26, -0.20) | -0.00 (-0.00, -0.00) | 0.00 (-0.00, 0.00) | -0.00 (-0.00, -0.00) | 0.03 (0.02, 0.04) | -0.01 (-0.02, 0.00) | -0.12 (-0.15, -0.09) | -0.13 (-0.16, -0.10) |
| GlobalShrinkageFactor | 0.93 (0.91, 0.94) | NA | 0.06 (0.05, 0.07) | 0.06 (0.05, 0.07) | 0.06 (0.05, 0.07) | -0.02 (-0.03, -0.01) | -0.04 (-0.06, -0.03) | -0.11 (-0.17, -0.07) | -0.13 (-0.17, -0.08) |
| IDI_diff1 | 0.23 (0.20, 0.26) | -0.23 (-0.26, -0.20) | -0.00 (-0.00, -0.00) | 0.00 (-0.00, 0.00) | -0.00 (-0.00, -0.00) | 0.03 (0.02, 0.04) | -0.01 (-0.02, 0.00) | -0.12 (-0.15, -0.09) | -0.13 (-0.16, -0.10) |
|  | | | | | | | | | |
| * The difference of performance measure = performance measure of each model - performance measure of the pseudo true model. | | | | | | | | | |
| ** The pseudo true model was the model considers all pseudo causal predictors in the variable set 1 and the interaction effects of age and sex. | | | | | | | | | |

| **eTable 11: Comparison of the model performance between the pseudo true model (consider interaction effects of age and sex) and other proxy models in NHANES 2017-2018 - part II** | | | | | | | | | | |
| --- | --- | --- | --- | --- | --- | --- | --- | --- | --- | --- |
|  | | | | | | | | | | |
|  |  | **Difference of model performance* between the other model and the Pseudo true model** (Mean (95% Range))** | | | | | | | | |
|  |  | **Models only consider LP1 and/or LP2** | |  |  |  |  |  |  |  |
|  |  | **consider LP1 and LP2** | **only consider LP2** | **Add one more variable while considering LP1 and LP2** | | **Missing LP1 but add one new variable while considering LP2** | | **Consider all variables or apply procedures considering all variables** | | |
|  | PseudoTrueModel | TwoVarModel | Univariate model 2 | AddLowCorr | AddHighCorr | AddLowCorr | AddHighCorr | FullModel | AICFullModel | ShrinkFullModel |
| AUC | 0.85 (0.82, 0.88) | 0.00 (-0.00, 0.01) | -0.11 (-0.15, -0.07) | 0.00 (-0.00, 0.01) | 0.00 (-0.00, 0.01) | -0.12 (-0.16, -0.08) | -0.11 (-0.15, -0.07) | 0.01 (-0.00, 0.01) | 0.01 (-0.00, 0.01) | 0.01 (0.00, 0.02) |
| Dxy | 0.70 (0.65, 0.76) | 0.00 (-0.01, 0.01) | -0.22 (-0.30, -0.14) | 0.00 (-0.01, 0.01) | 0.01 (-0.00, 0.02) | -0.24 (-0.32, -0.16) | -0.22 (-0.30, -0.14) | 0.01 (-0.01, 0.03) | 0.01 (-0.01, 0.03) | 0.02 (0.00, 0.03) |
| gIndex | 0.17 (0.16, 0.18) | 0.00 (0.00, 0.01) | -0.08 (-0.10, -0.07) | 0.00 (0.00, 0.01) | 0.00 (0.00, 0.01) | -0.08 (-0.10, -0.07) | -0.08 (-0.10, -0.07) | 0.01 (0.01, 0.02) | 0.01 (0.01, 0.02) | -0.01 (-0.01, -0.00) |
| U | 0.01 (-0.00, 0.02) | -0.00 (-0.00, 0.00) | 0.01 (-0.00, 0.02) | -0.00 (-0.00, -0.00) | -0.00 (-0.00, 0.00) | 0.01 (0.00, 0.02) | 0.01 (-0.00, 0.02) | -0.00 (-0.01, 0.00) | -0.00 (-0.01, 0.00) | 0.00 (-0.01, 0.01) |
| D | 0.24 (0.19, 0.30) | 0.00 (-0.01, 0.01) | -0.16 (-0.22, -0.11) | 0.00 (-0.00, 0.01) | 0.01 (-0.00, 0.02) | -0.17 (-0.22, -0.11) | -0.16 (-0.22, -0.11) | 0.01 (-0.01, 0.03) | 0.01 (-0.01, 0.03) | 0.01 (-0.00, 0.03) |
| Q | 0.23 (0.18, 0.28) | 0.01 (-0.00, 0.01) | -0.17 (-0.22, -0.11) | 0.01 (-0.00, 0.02) | 0.01 (0.00, 0.02) | -0.18 (-0.23, -0.12) | -0.17 (-0.22, -0.11) | 0.01 (-0.01, 0.03) | 0.01 (-0.01, 0.03) | 0.01 (-0.00, 0.03) |
| OR_caliLarge | 0.82 (0.70, 0.94) | 0.02 (0.01, 0.03) | -0.09 (-0.15, -0.02) | 0.02 (0.01, 0.03) | 0.01 (0.00, 0.03) | -0.09 (-0.16, -0.03) | -0.09 (-0.15, -0.02) | 0.03 (0.00, 0.05) | 0.03 (0.00, 0.05) | 0.01 (-0.01, 0.03) |
| Emax_ab | 0.07 (0.02, 0.14) | -0.01 (-0.02, 0.01) | 0.04 (-0.05, 0.17) | -0.01 (-0.02, 0.01) | -0.00 (-0.02, 0.01) | 0.03 (-0.05, 0.16) | 0.04 (-0.05, 0.17) | -0.01 (-0.04, 0.02) | -0.01 (-0.04, 0.02) | 0.03 (-0.01, 0.06) |
| E_abMean | 0.03 (0.01, 0.05) | -0.00 (-0.00, -0.00) | 0.01 (0.00, 0.02) | -0.00 (-0.00, -0.00) | -0.00 (-0.00, 0.00) | 0.01 (0.00, 0.02) | 0.01 (0.00, 0.02) | -0.00 (-0.01, 0.00) | -0.00 (-0.01, 0.00) | -0.00 (-0.01, 0.01) |
| Emax_01 | 0.07 (0.02, 0.14) | -0.01 (-0.02, 0.01) | 0.04 (-0.05, 0.17) | -0.01 (-0.02, 0.01) | -0.00 (-0.02, 0.01) | 0.03 (-0.05, 0.16) | 0.04 (-0.05, 0.17) | -0.01 (-0.04, 0.02) | -0.01 (-0.04, 0.02) | 0.03 (-0.01, 0.06) |
| E_01Mean | 0.04 (0.01, 0.09) | -0.00 (-0.01, 0.00) | 0.02 (-0.03, 0.10) | -0.00 (-0.01, 0.00) | -0.00 (-0.01, 0.01) | 0.02 (-0.04, 0.09) | 0.02 (-0.03, 0.10) | -0.01 (-0.03, 0.01) | -0.01 (-0.03, 0.01) | 0.02 (-0.01, 0.03) |
| Eavg_LOWESS | 0.03 (0.01, 0.05) | -0.00 (-0.01, 0.00) | 0.03 (0.01, 0.04) | -0.00 (-0.01, 0.00) | -0.00 (-0.01, 0.00) | 0.02 (0.01, 0.04) | 0.03 (0.01, 0.04) | -0.00 (-0.01, 0.00) | -0.00 (-0.01, 0.00) | 0.00 (-0.01, 0.01) |
| ECI_LOWESS | 0.19 (0.04, 0.45) | -0.04 (-0.09, 0.01) | 0.73 (0.30, 1.27) | -0.04 (-0.09, 0.01) | -0.03 (-0.09, 0.01) | 0.73 (0.30, 1.26) | 0.73 (0.30, 1.27) | -0.06 (-0.16, 0.02) | -0.06 (-0.16, 0.02) | 0.02 (-0.12, 0.14) |
| brierScore | 0.11 (0.09, 0.12) | -0.00 (-0.00, 0.00) | 0.03 (0.02, 0.03) | -0.00 (-0.00, 0.00) | -0.00 (-0.00, -0.00) | 0.03 (0.02, 0.04) | 0.03 (0.02, 0.03) | -0.00 (-0.01, 0.00) | -0.00 (-0.01, 0.00) | -0.00 (-0.01, 0.00) |
| R2_Nagelkerke | 0.36 (0.29, 0.43) | 0.00 (-0.01, 0.02) | -0.23 (-0.30, -0.16) | 0.01 (-0.01, 0.02) | 0.01 (-0.00, 0.03) | -0.24 (-0.31, -0.17) | -0.23 (-0.30, -0.16) | 0.01 (-0.02, 0.04) | 0.01 (-0.01, 0.04) | 0.02 (-0.00, 0.04) |
| R2_McFadden | 0.27 (0.22, 0.32) | 0.00 (-0.01, 0.01) | -0.18 (-0.23, -0.12) | 0.01 (-0.00, 0.01) | 0.01 (-0.00, 0.03) | -0.19 (-0.24, -0.13) | -0.18 (-0.23, -0.12) | 0.01 (-0.01, 0.03) | 0.01 (-0.01, 0.03) | 0.02 (-0.00, 0.04) |
| R2_McFaddenAdj | 0.23 (0.18, 0.28) | 0.04 (0.03, 0.05) | -0.15 (-0.20, -0.09) | 0.04 (0.02, 0.05) | 0.04 (0.03, 0.06) | -0.15 (-0.21, -0.09) | -0.15 (-0.20, -0.09) | -0.04 (-0.06, -0.01) | -0.03 (-0.06, -0.01) | -0.03 (-0.05, -0.01) |
| R2_CoxSnell | 0.22 (0.17, 0.26) | 0.00 (-0.00, 0.01) | -0.14 (-0.18, -0.09) | 0.00 (-0.00, 0.01) | 0.01 (-0.00, 0.02) | -0.14 (-0.18, -0.10) | -0.14 (-0.18, -0.09) | 0.01 (-0.01, 0.02) | 0.01 (-0.01, 0.02) | 0.01 (-0.00, 0.02) |
| R2_AldrichNelson | 0.20 (0.16, 0.23) | 0.00 (-0.00, 0.01) | -0.12 (-0.16, -0.08) | 0.00 (-0.00, 0.01) | 0.01 (-0.00, 0.01) | -0.13 (-0.16, -0.09) | -0.12 (-0.16, -0.08) | 0.01 (-0.01, 0.02) | 0.01 (-0.01, 0.02) | 0.01 (-0.00, 0.02) |
| R2_VeallZimmermann | 0.41 (0.34, 0.48) | 0.01 (-0.01, 0.02) | -0.26 (-0.33, -0.17) | 0.01 (-0.01, 0.02) | 0.01 (-0.00, 0.03) | -0.27 (-0.34, -0.18) | -0.26 (-0.33, -0.17) | 0.01 (-0.02, 0.04) | 0.01 (-0.02, 0.04) | 0.02 (-0.00, 0.04) |
| R2_Efron | 0.24 (0.18, 0.29) | 0.01 (-0.00, 0.02) | -0.18 (-0.24, -0.12) | 0.01 (-0.00, 0.02) | 0.02 (0.00, 0.03) | -0.19 (-0.24, -0.12) | -0.18 (-0.24, -0.12) | 0.02 (-0.01, 0.04) | 0.02 (-0.01, 0.04) | 0.02 (-0.00, 0.04) |
| R2_McKelveyZavoina | 0.45 (0.44, 0.47) | 0.01 (0.01, 0.01) | -0.32 (-0.34, -0.29) | 0.01 (0.01, 0.01) | 0.01 (0.01, 0.02) | -0.32 (-0.34, -0.29) | -0.32 (-0.34, -0.29) | 0.03 (0.02, 0.04) | 0.03 (0.02, 0.04) | -0.05 (-0.05, -0.03) |
| R2_Tjur | 0.23 (0.20, 0.26) | 0.01 (0.00, 0.02) | -0.16 (-0.19, -0.13) | 0.01 (0.00, 0.02) | 0.01 (0.01, 0.02) | -0.16 (-0.19, -0.13) | -0.16 (-0.19, -0.13) | 0.02 (0.01, 0.04) | 0.02 (0.01, 0.04) | -0.00 (-0.01, 0.02) |
| GlobalShrinkageFactor | 0.93 (0.91, 0.94) | 0.06 (0.05, 0.07) | 0.05 (0.03, 0.06) | 0.06 (0.05, 0.07) | 0.06 (0.05, 0.07) | 0.03 (0.00, 0.05) | 0.03 (0.01, 0.05) | -0.08 (-0.11, -0.07) | -0.08 (-0.10, -0.06) | -0.08 (-0.10, -0.06) |
| IDI_diff1 | 0.23 (0.20, 0.26) | 0.01 (0.00, 0.02) | -0.16 (-0.19, -0.13) | 0.01 (0.00, 0.02) | 0.01 (0.01, 0.02) | -0.16 (-0.19, -0.13) | -0.16 (-0.19, -0.13) | 0.02 (0.01, 0.04) | 0.02 (0.01, 0.04) | -0.00 (-0.01, 0.02) |
|  | | | | | | | | | | |
| * The difference of performance measure = performance measure of each model - performance measure of the pseudo true model. | | | | | | | | | | |
| ** The pseudo true model was the model considers all pseudo causal predictors in the variable set 1 and the interaction effects of age and sex. | | | | | | | | | | |

**eFigure 1.2 – 1.25**


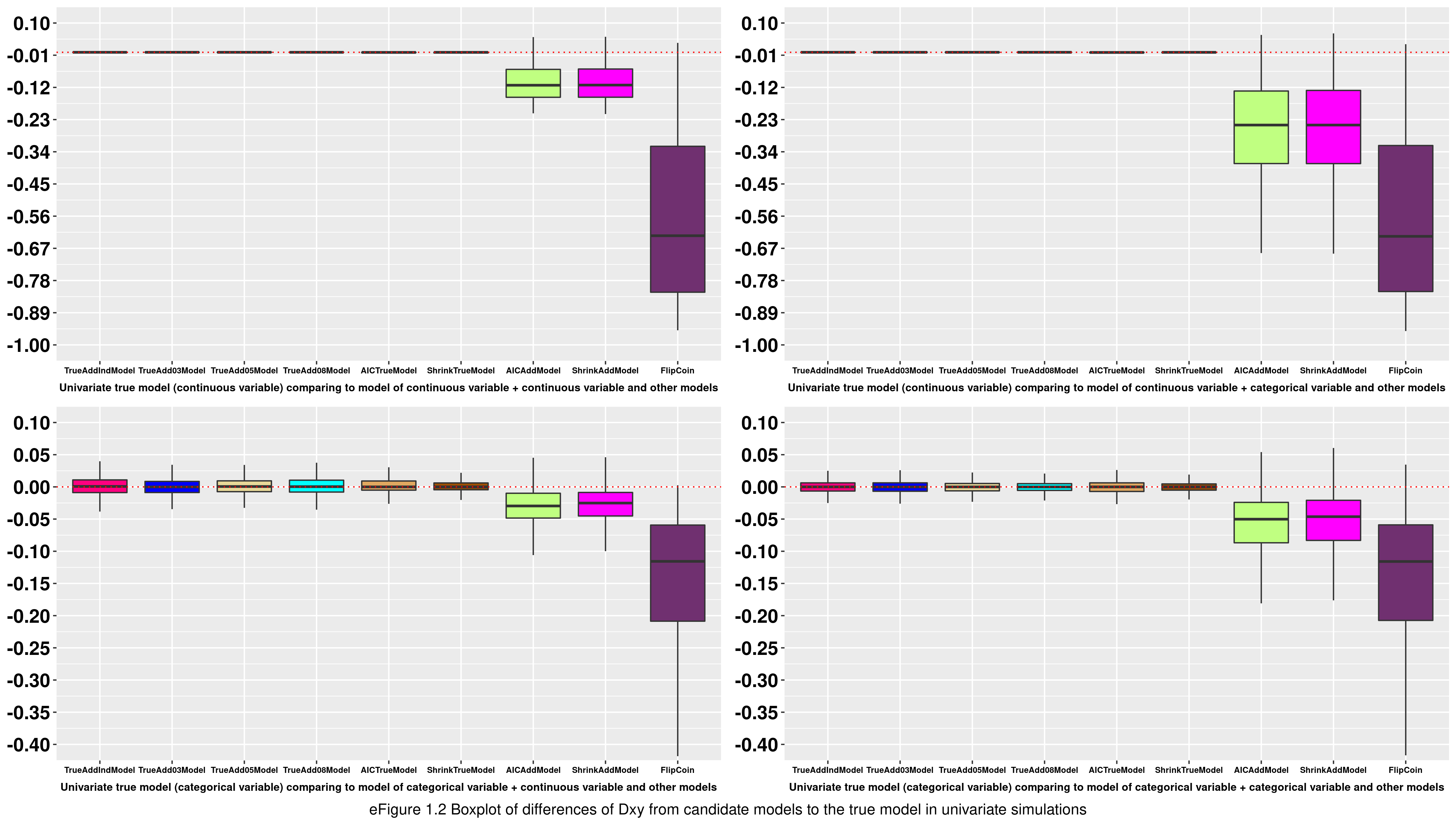

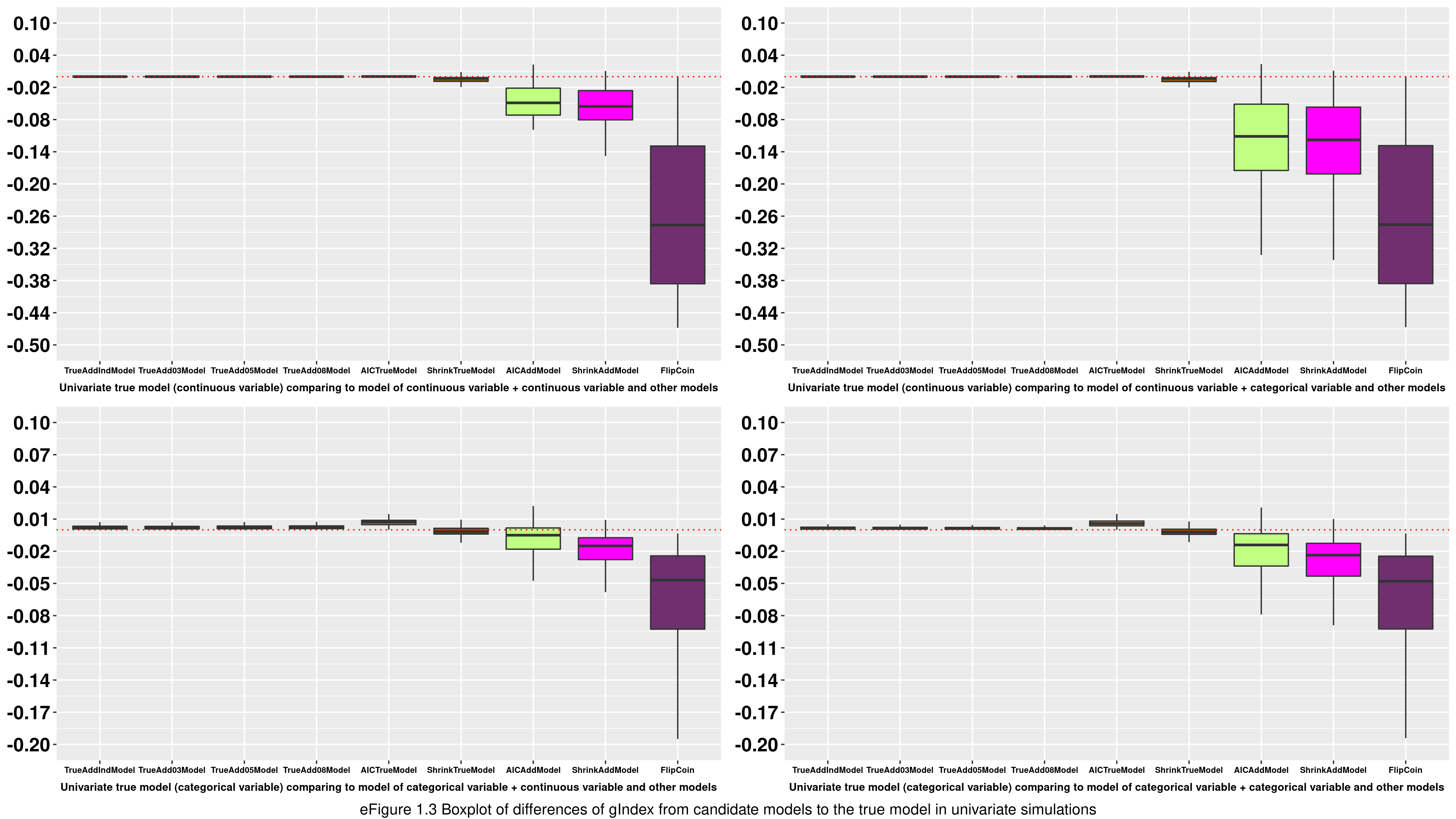

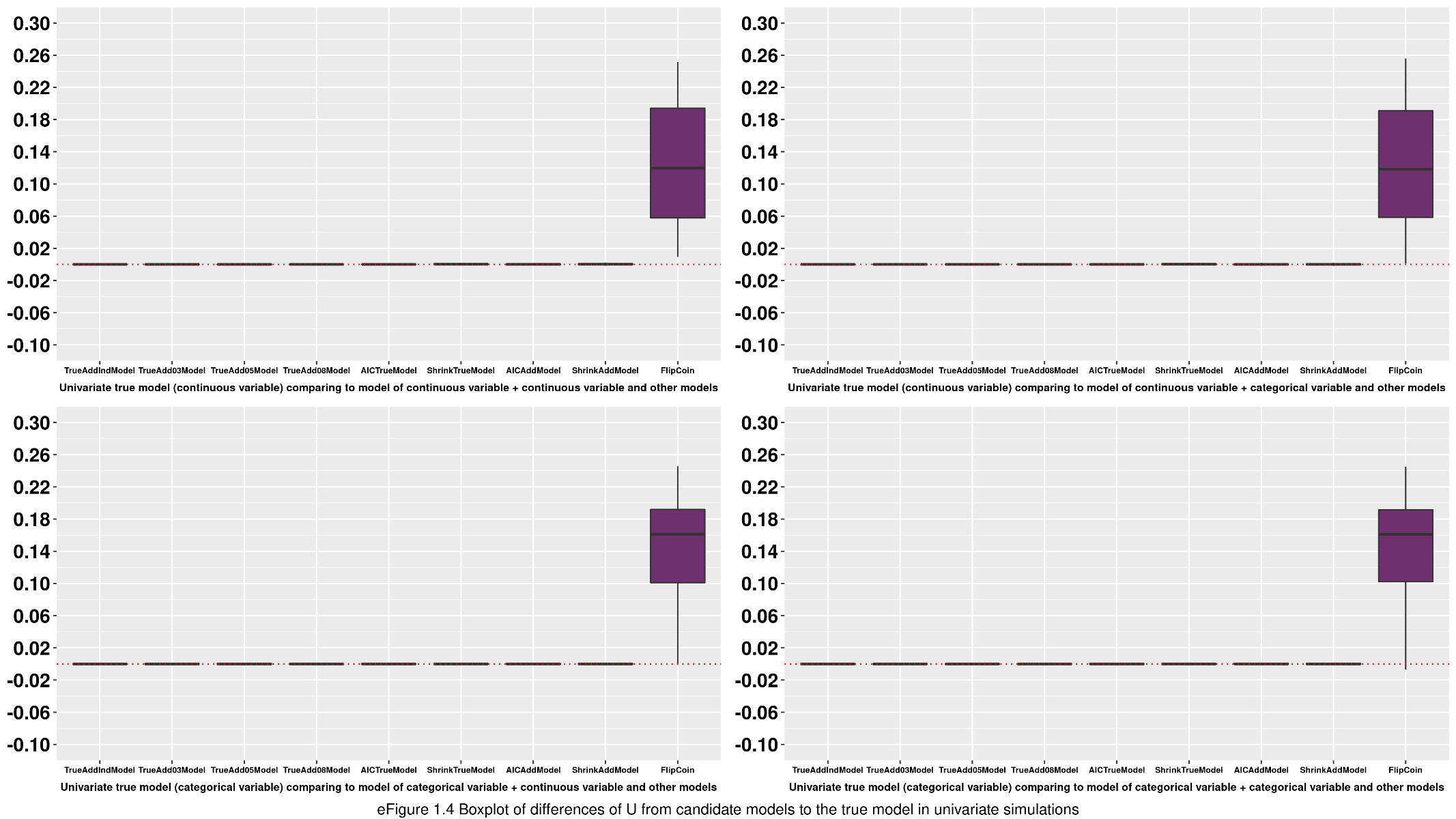

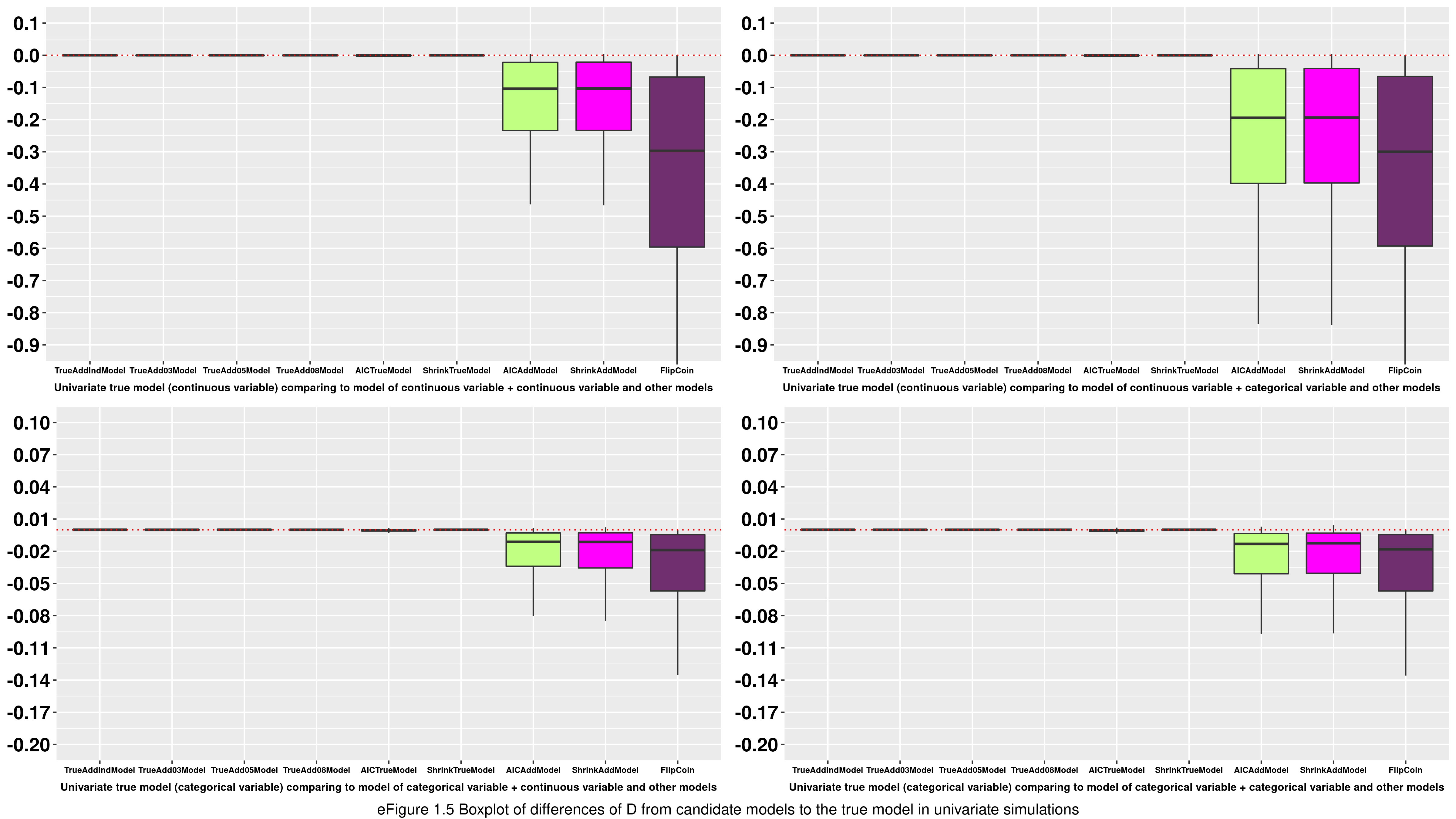

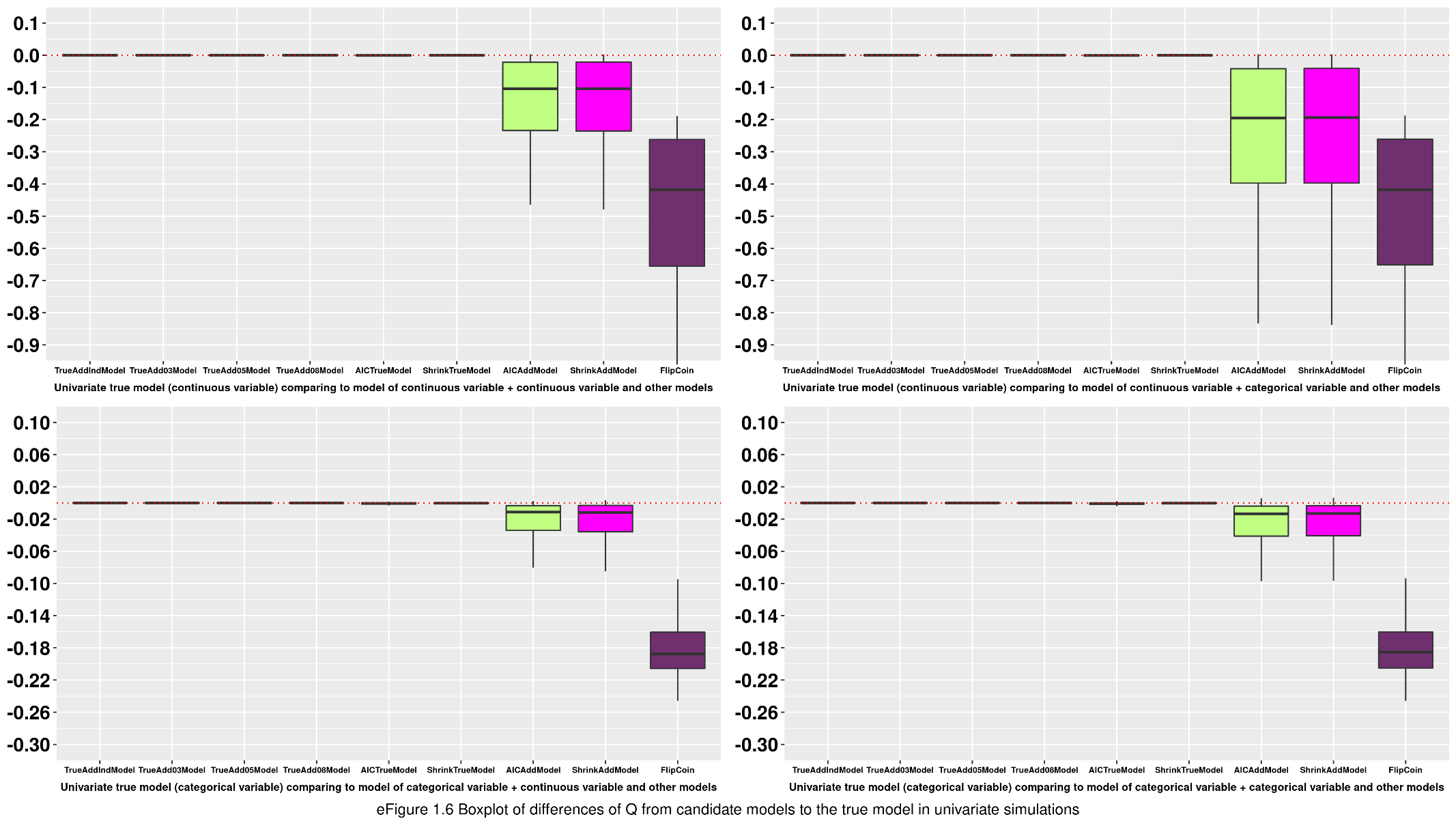

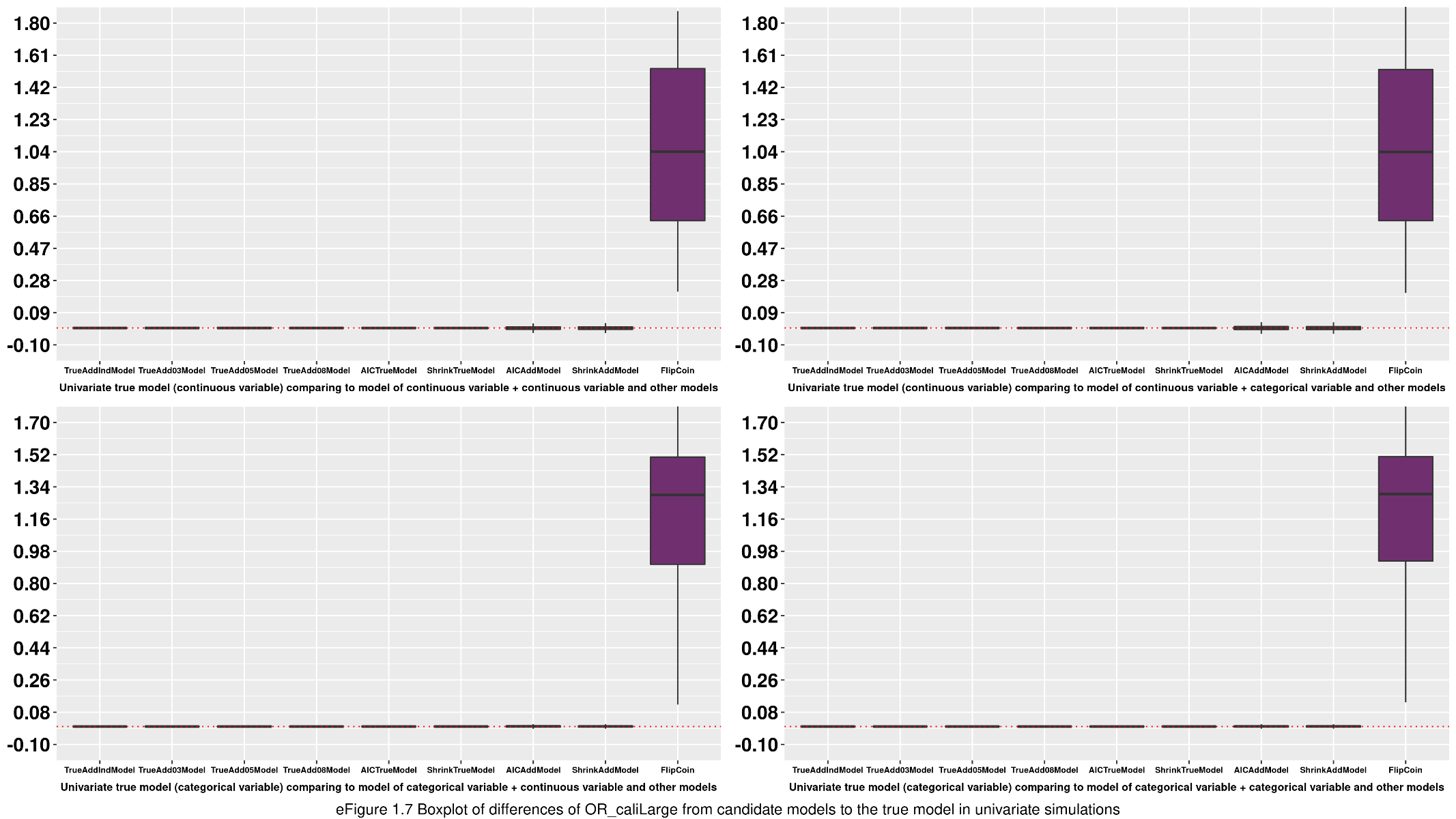

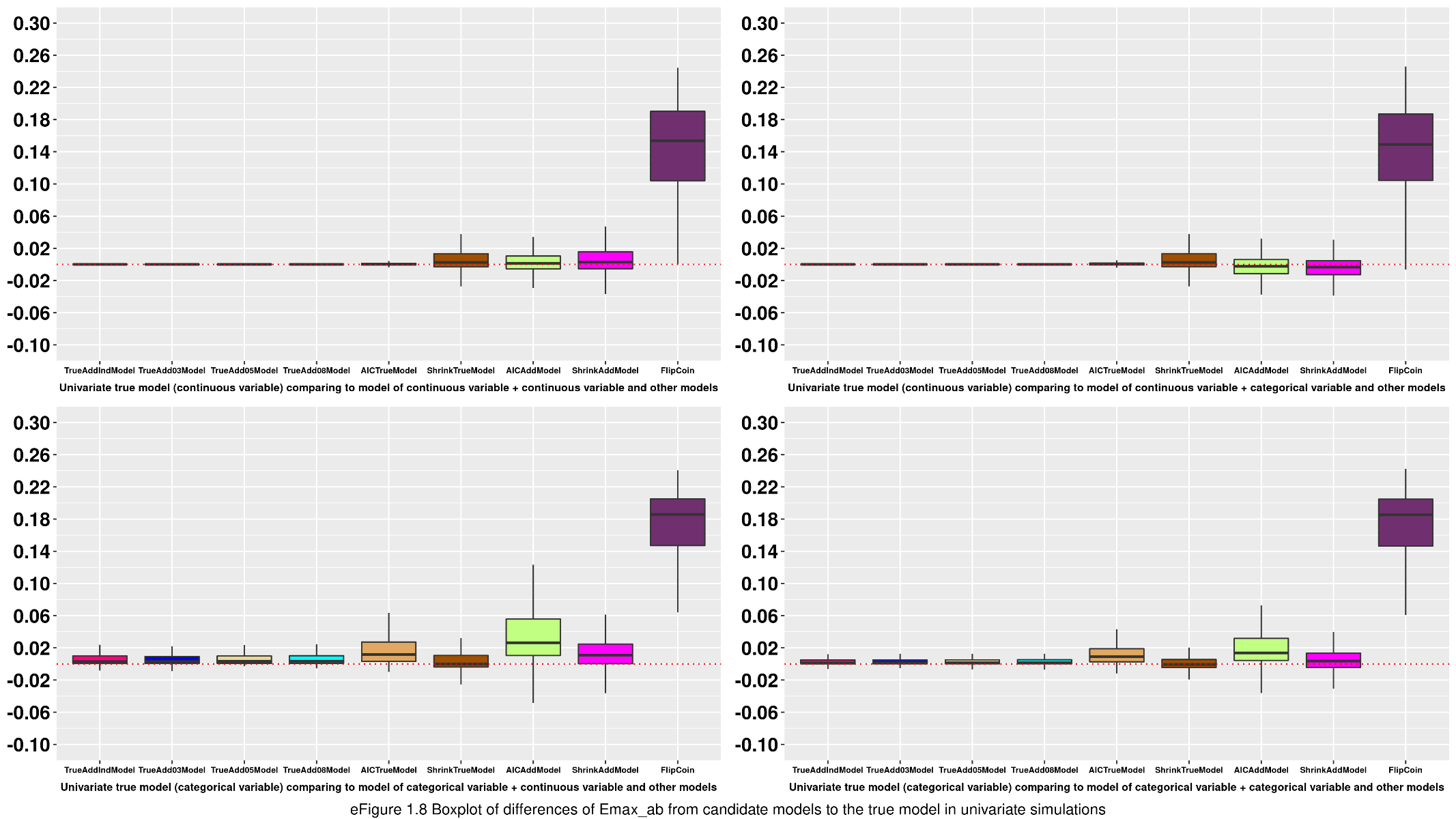

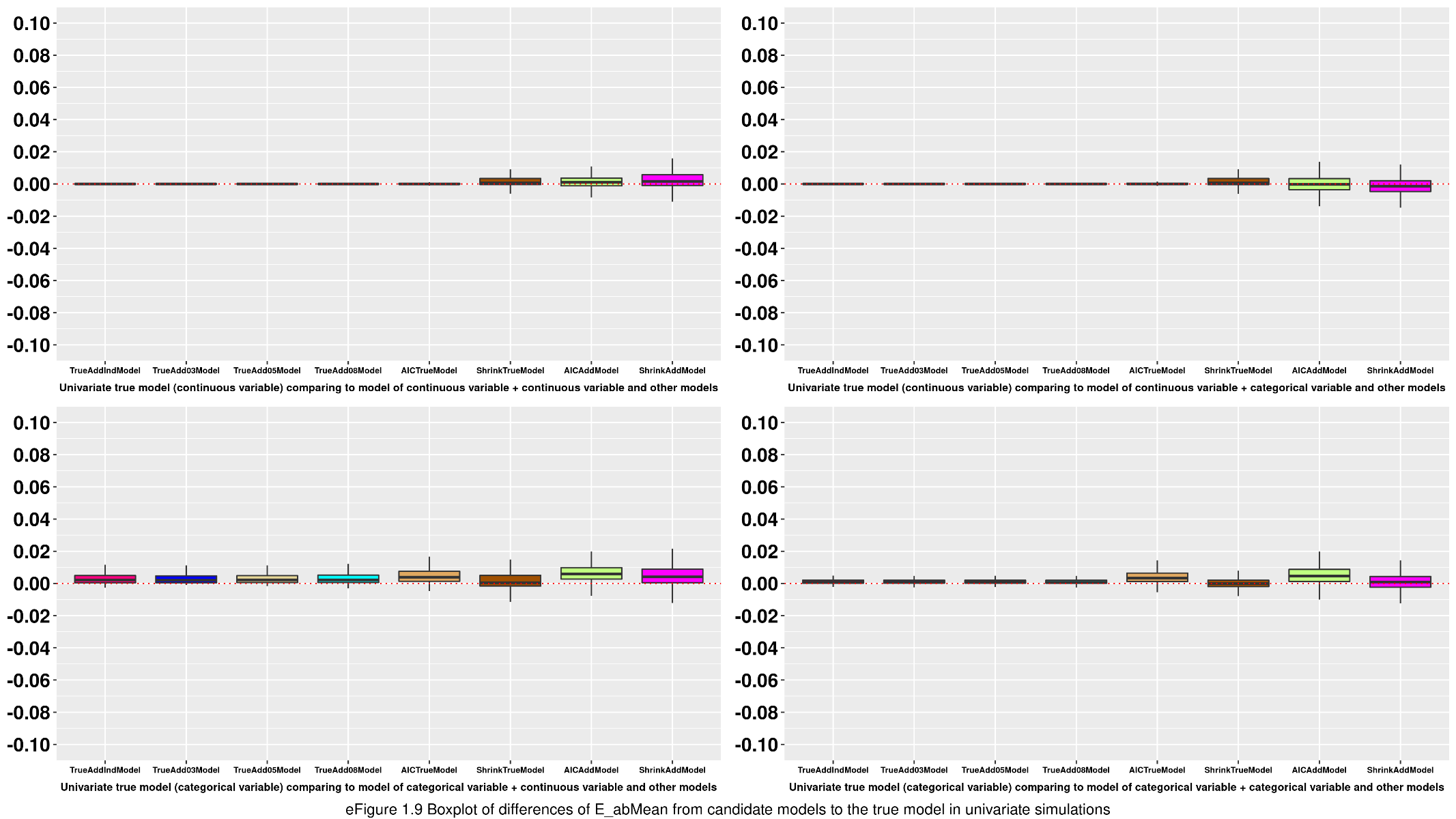

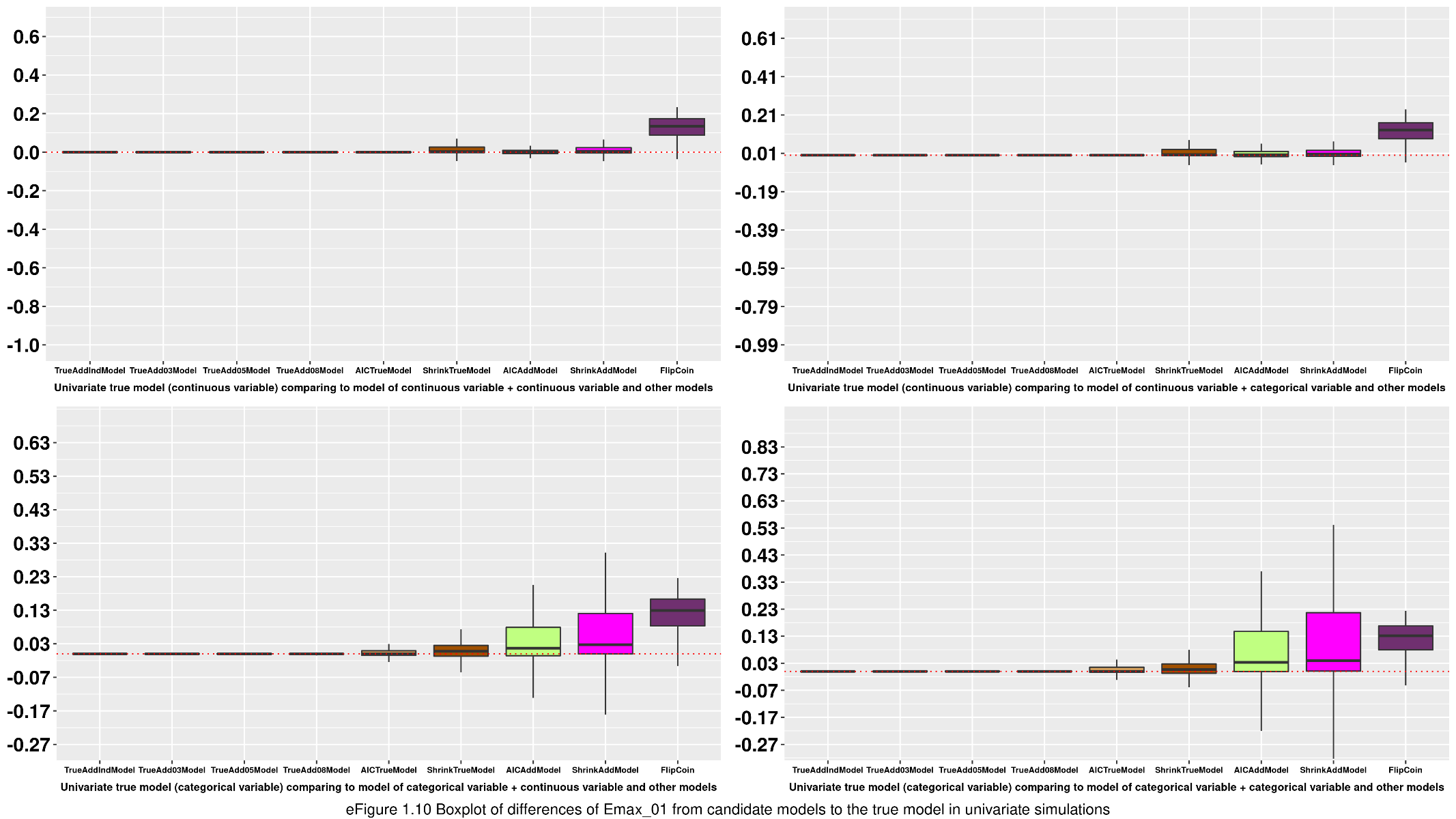

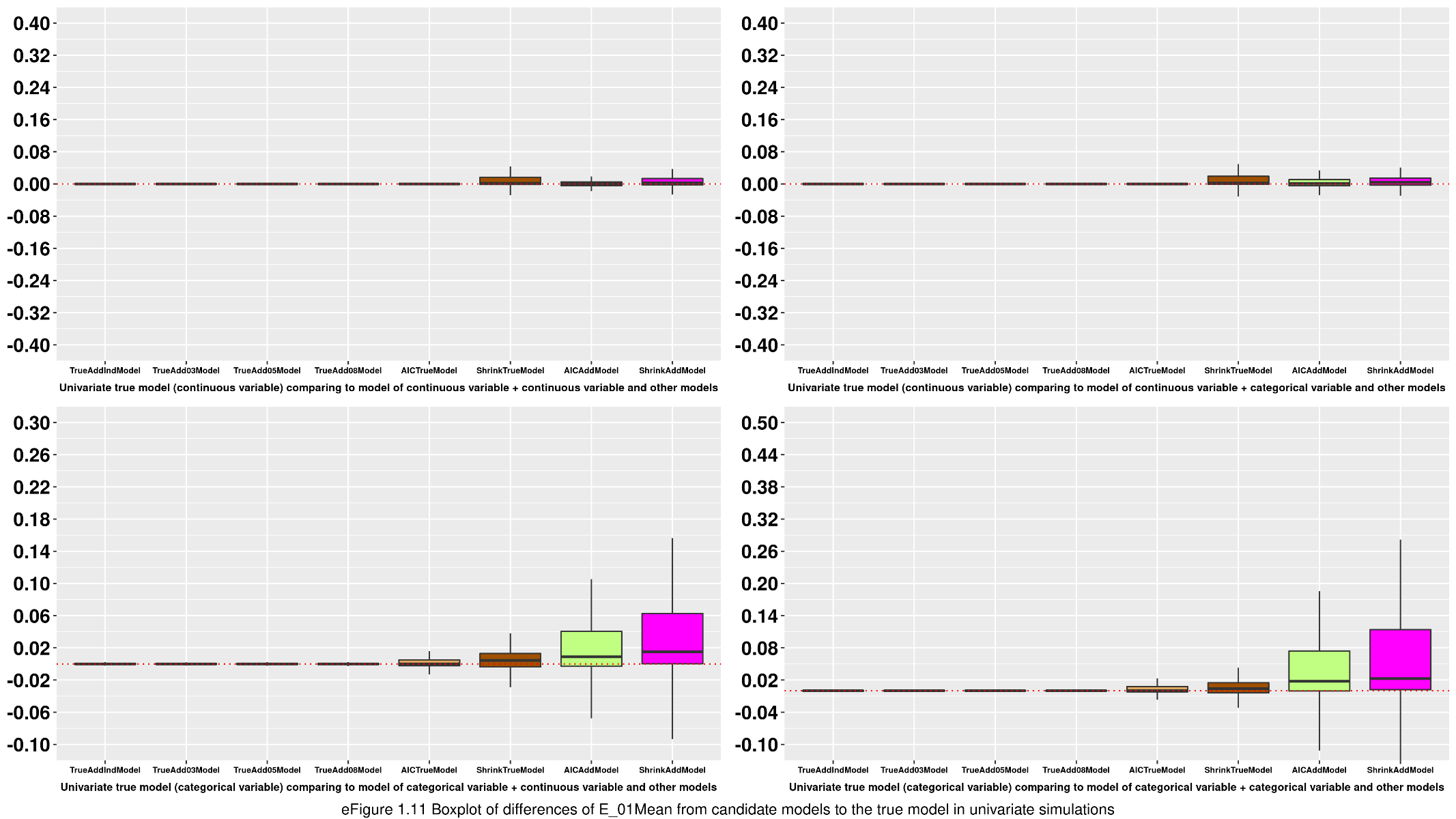

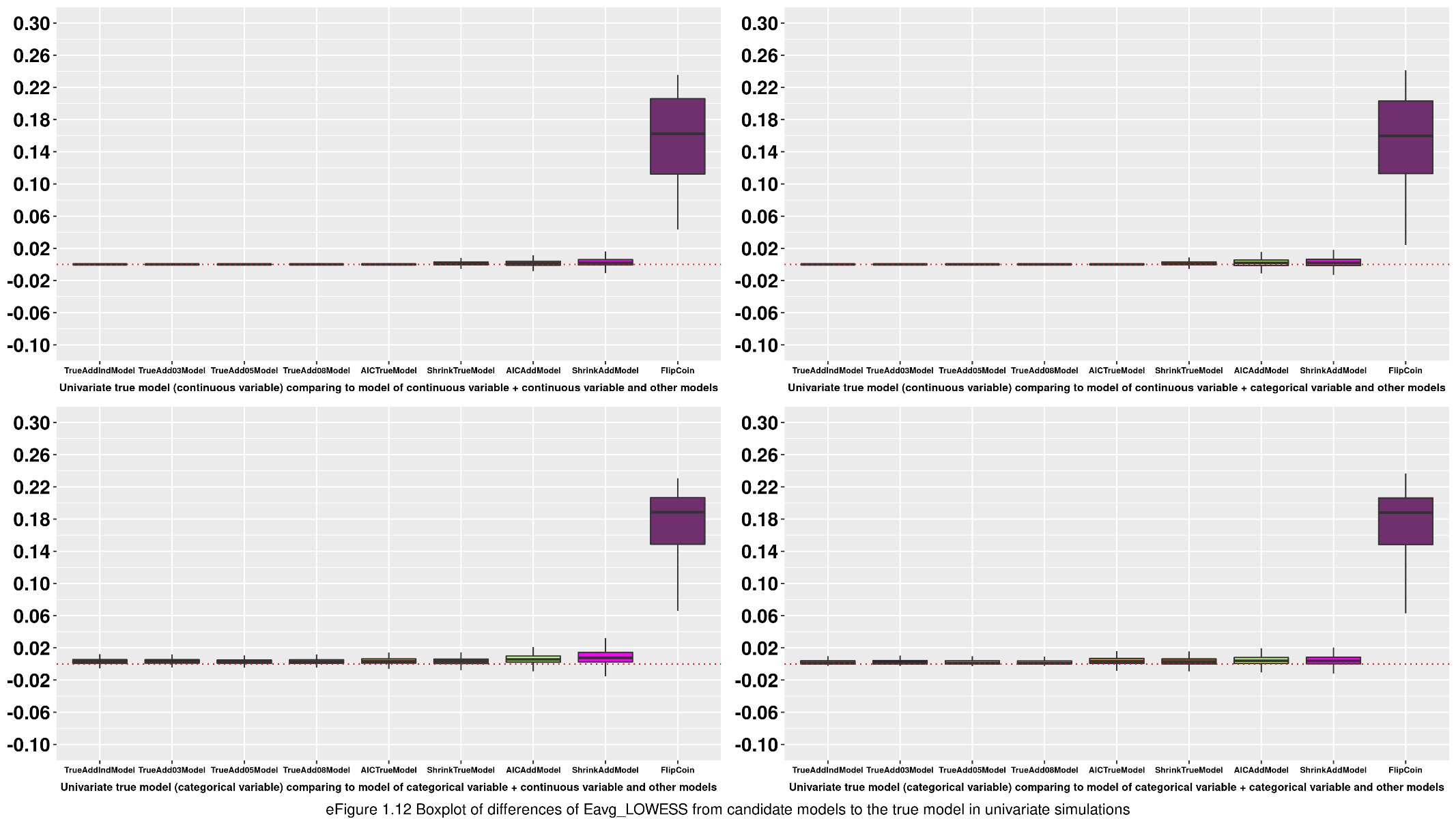

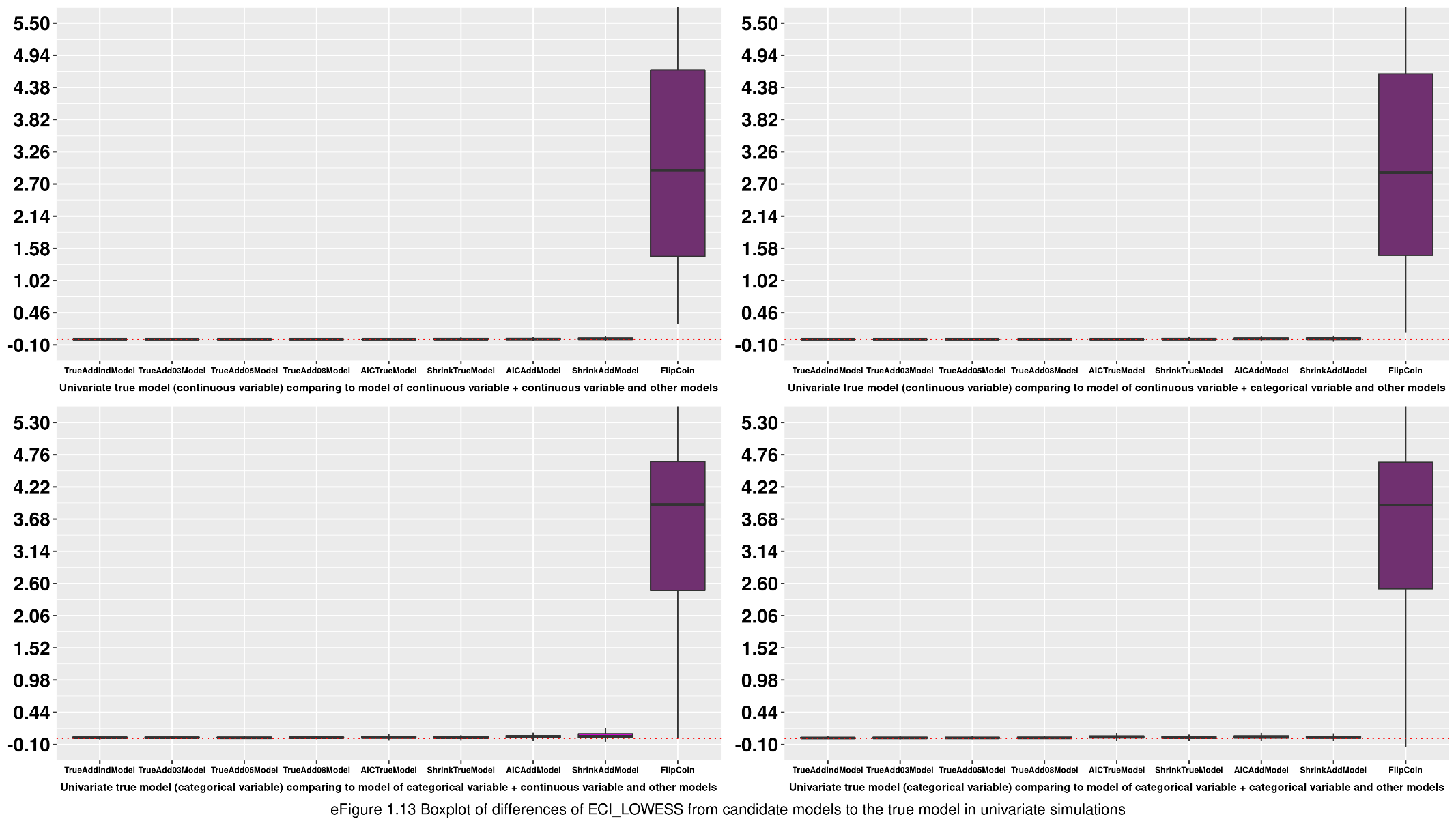

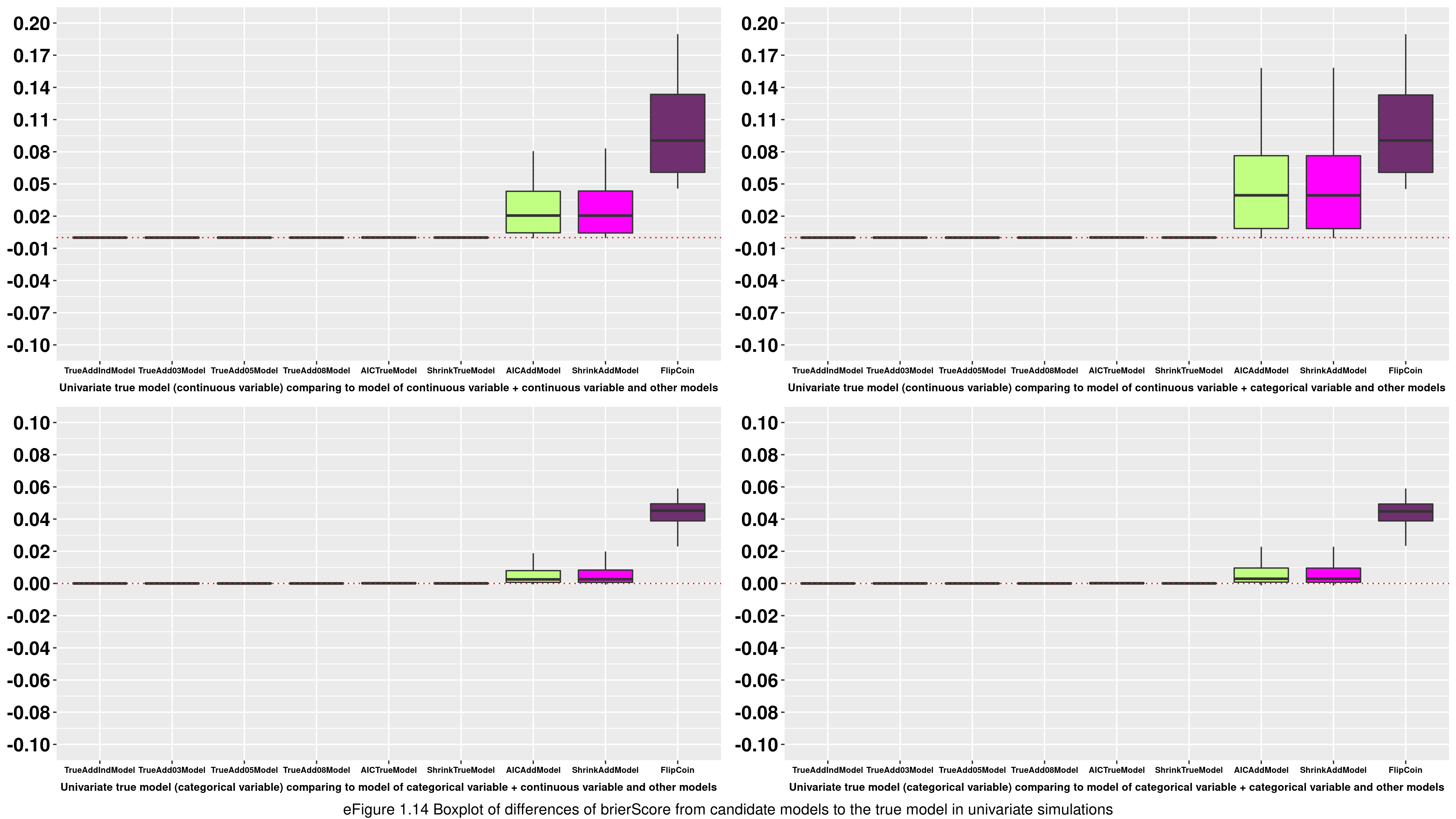

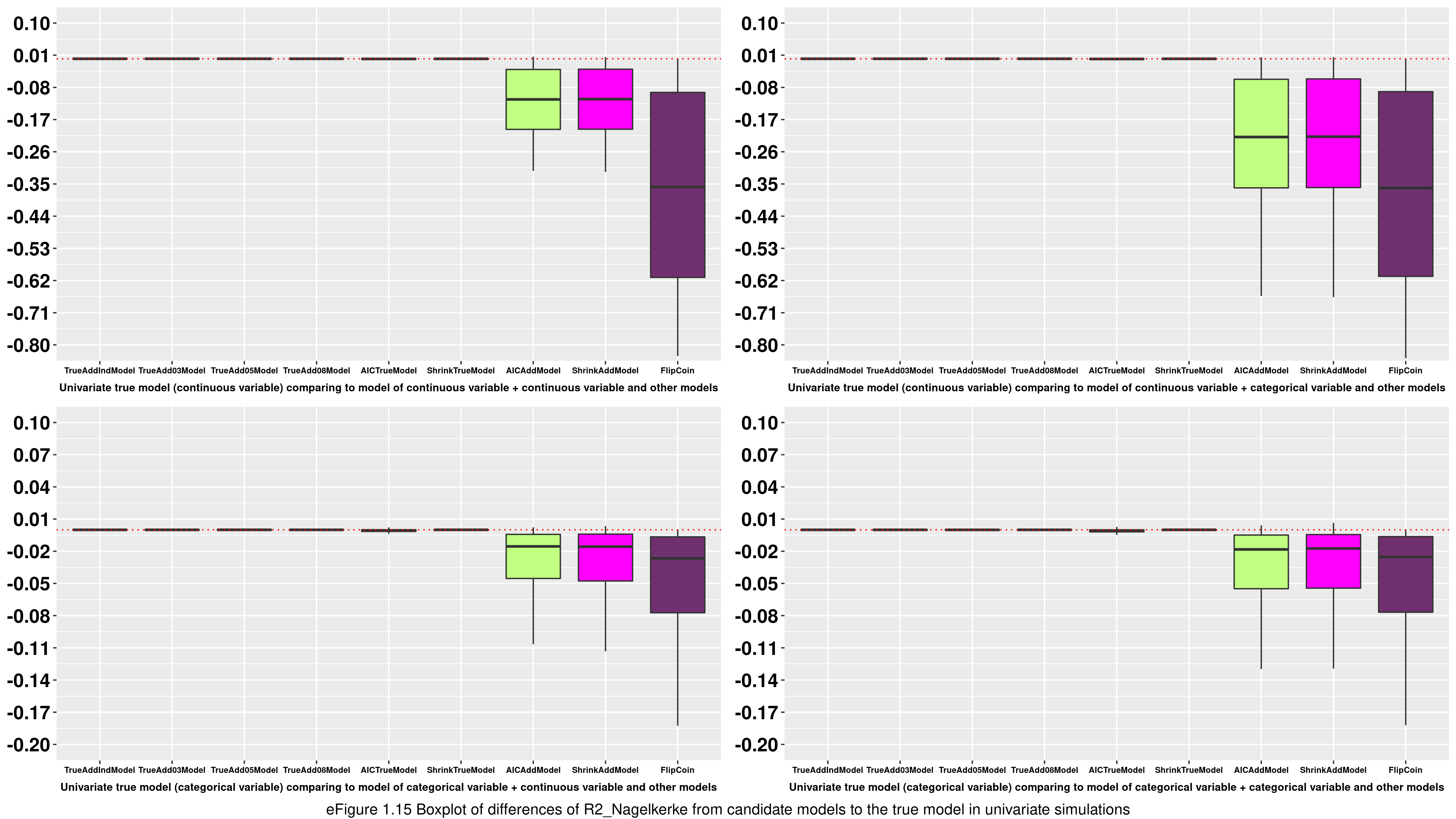

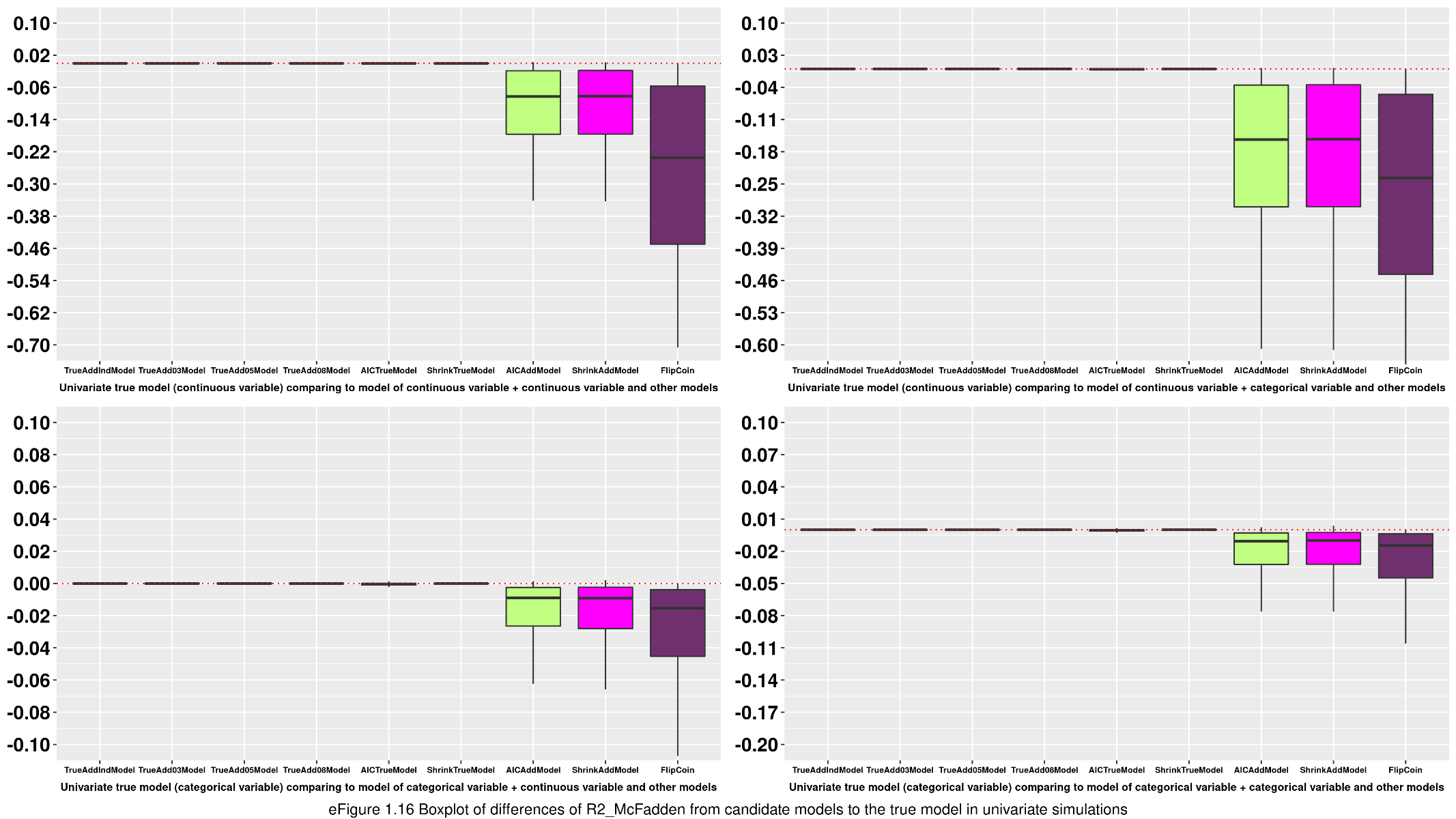

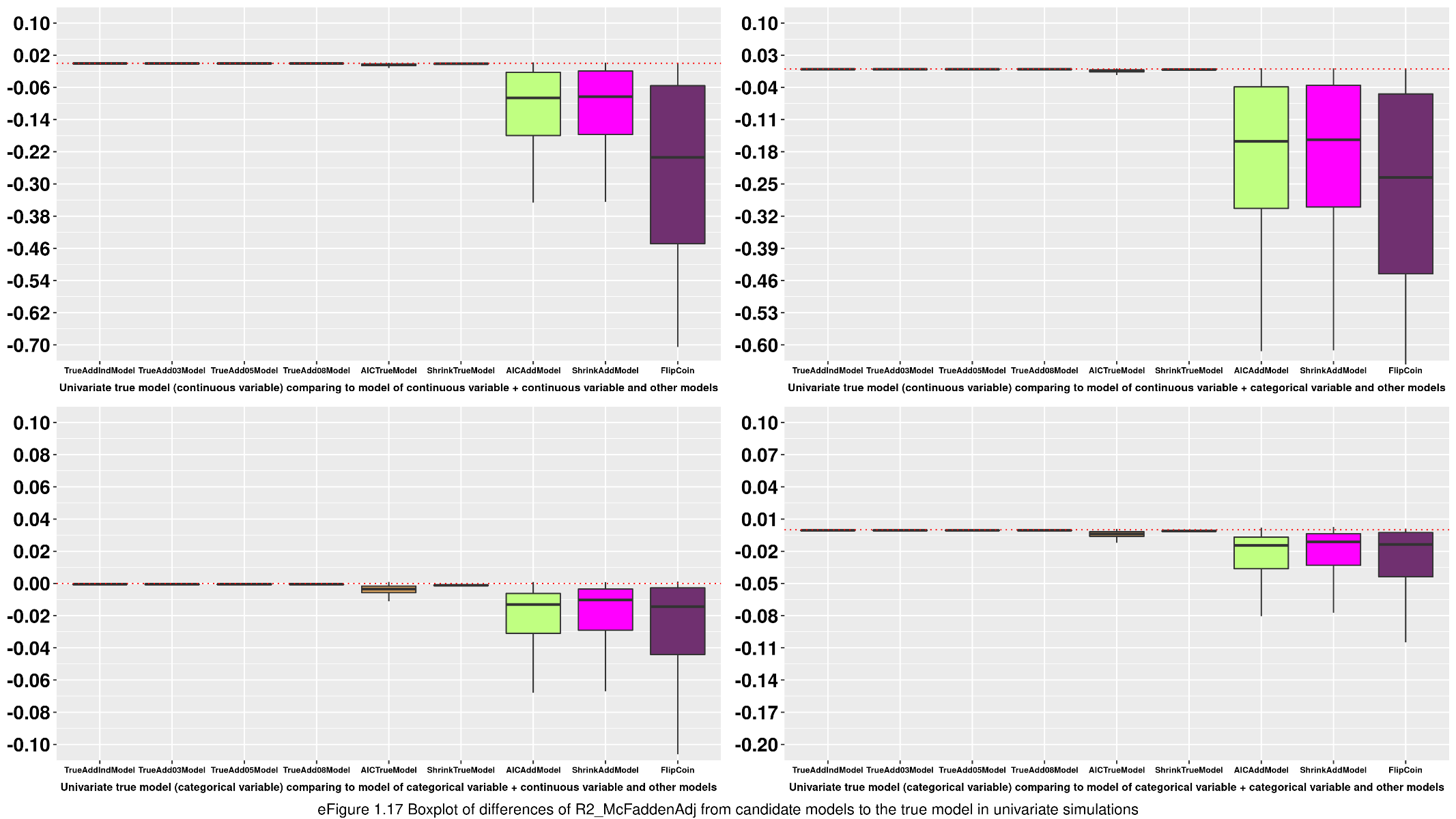

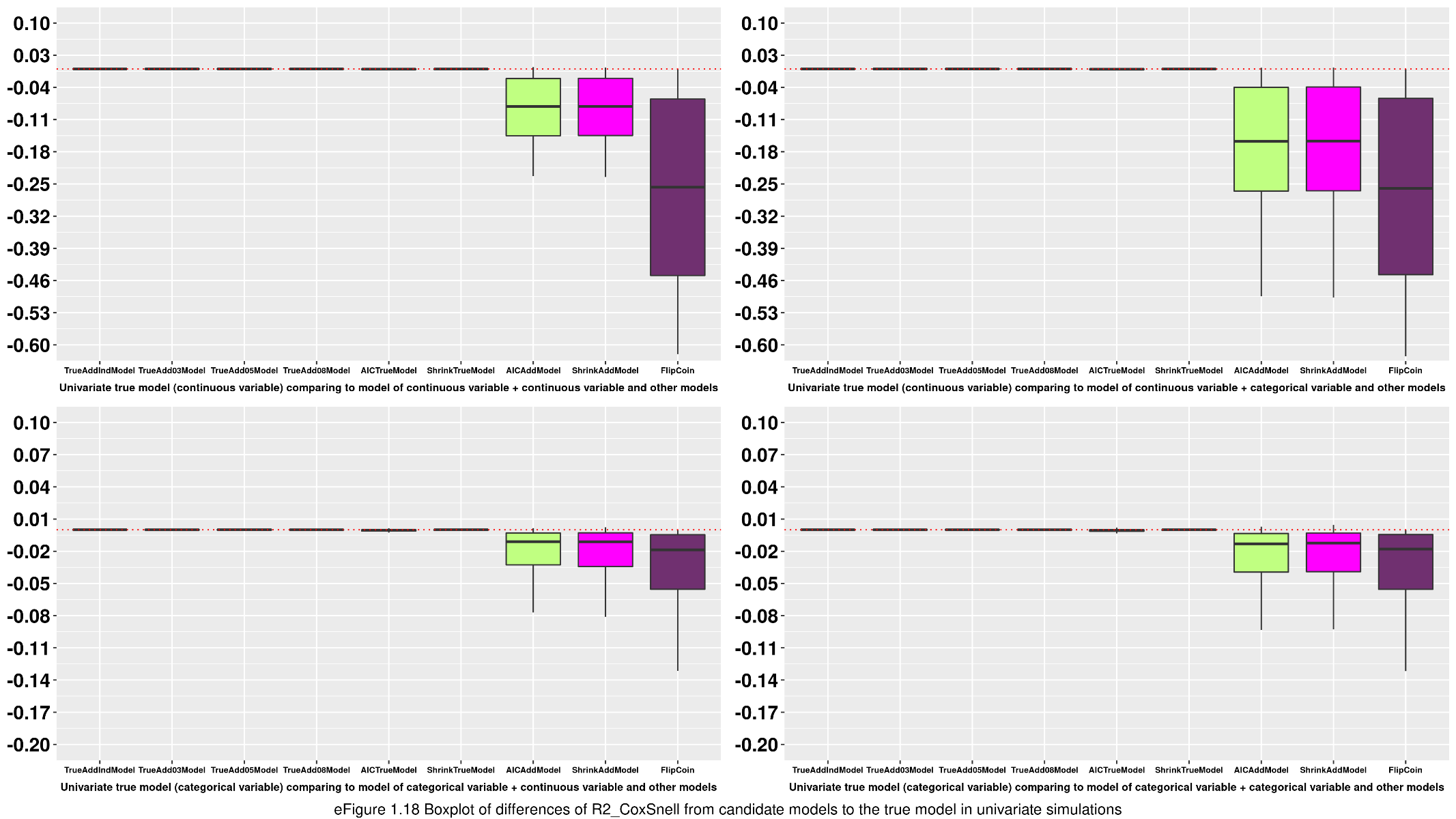

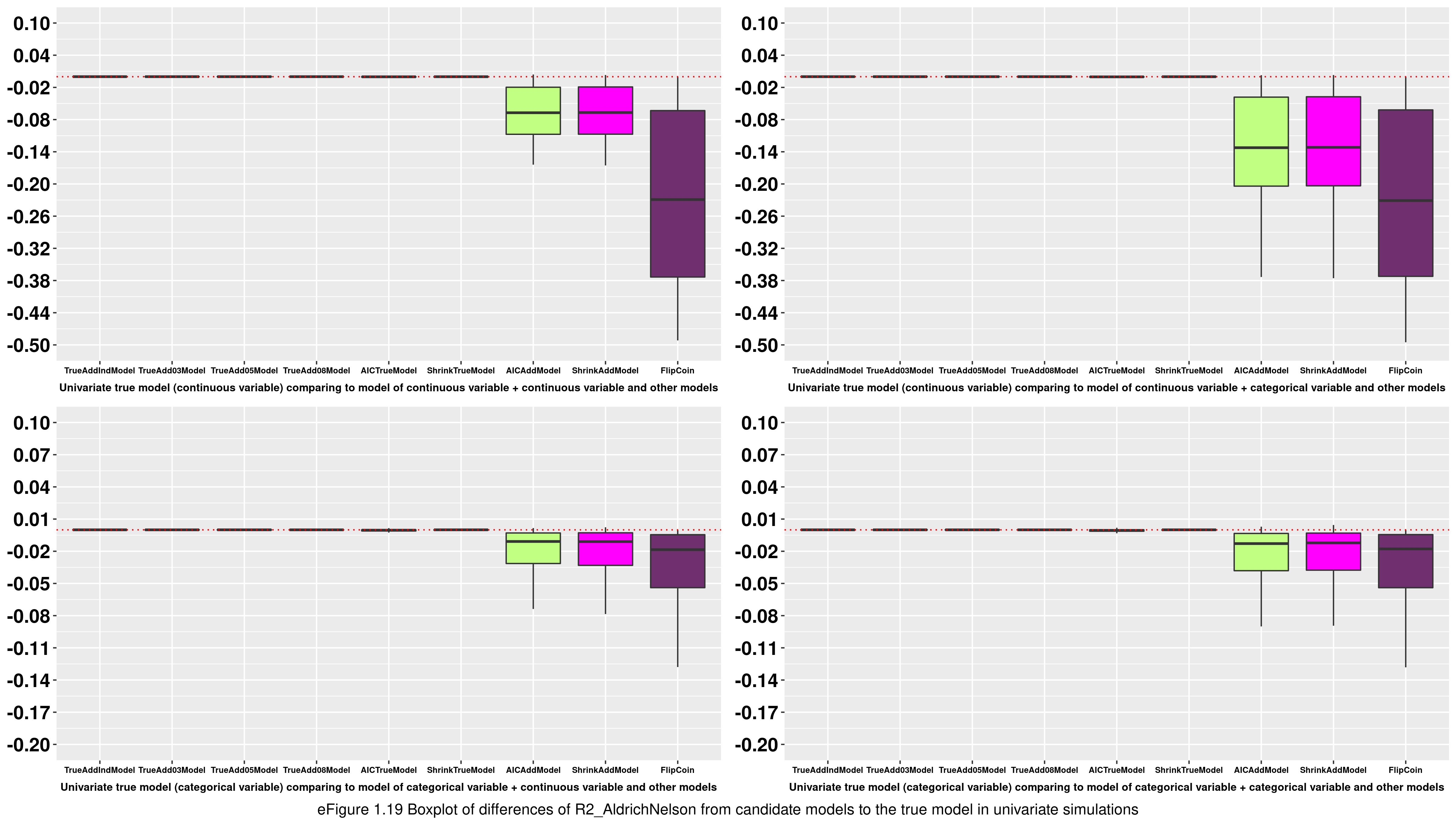

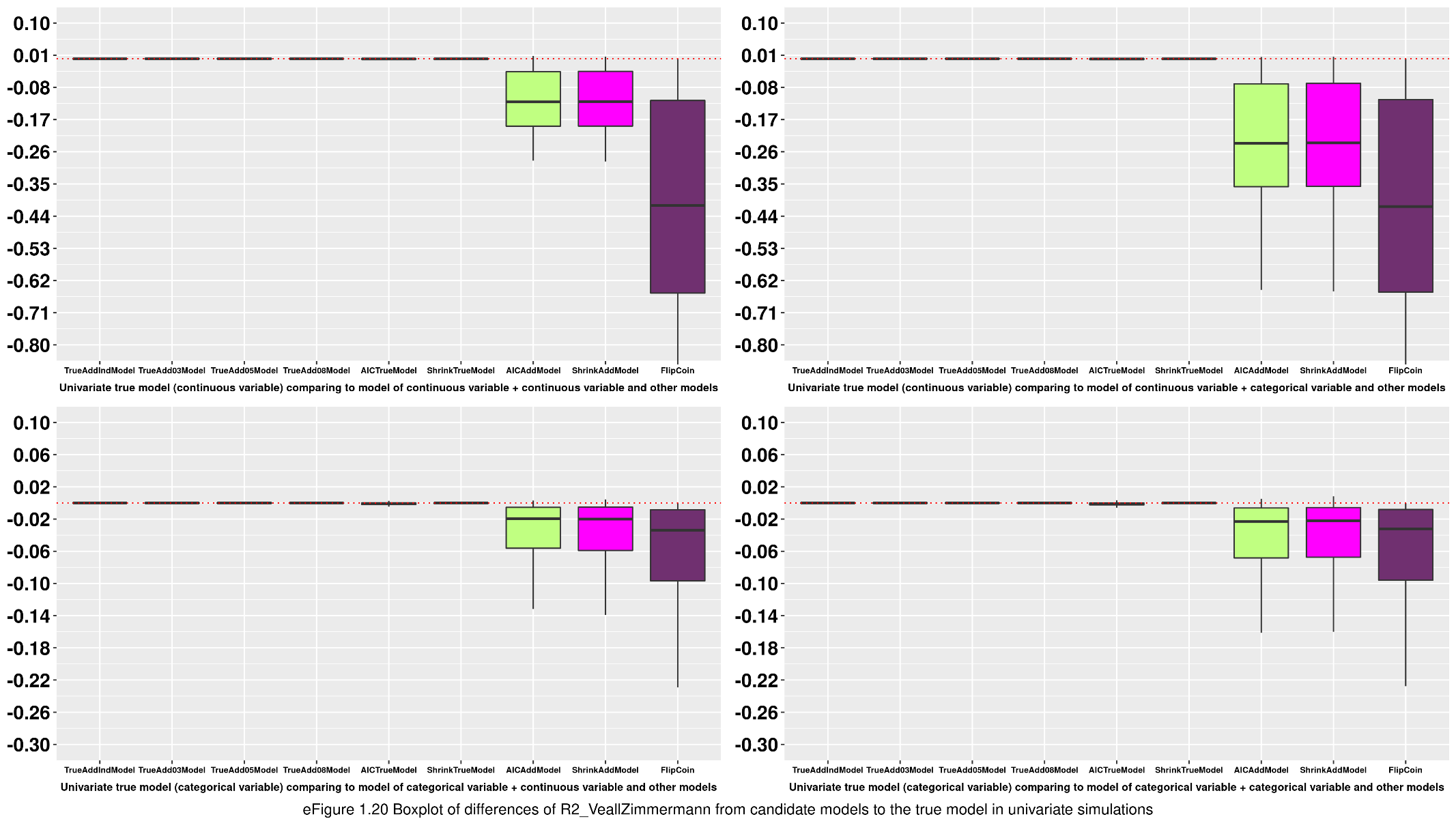

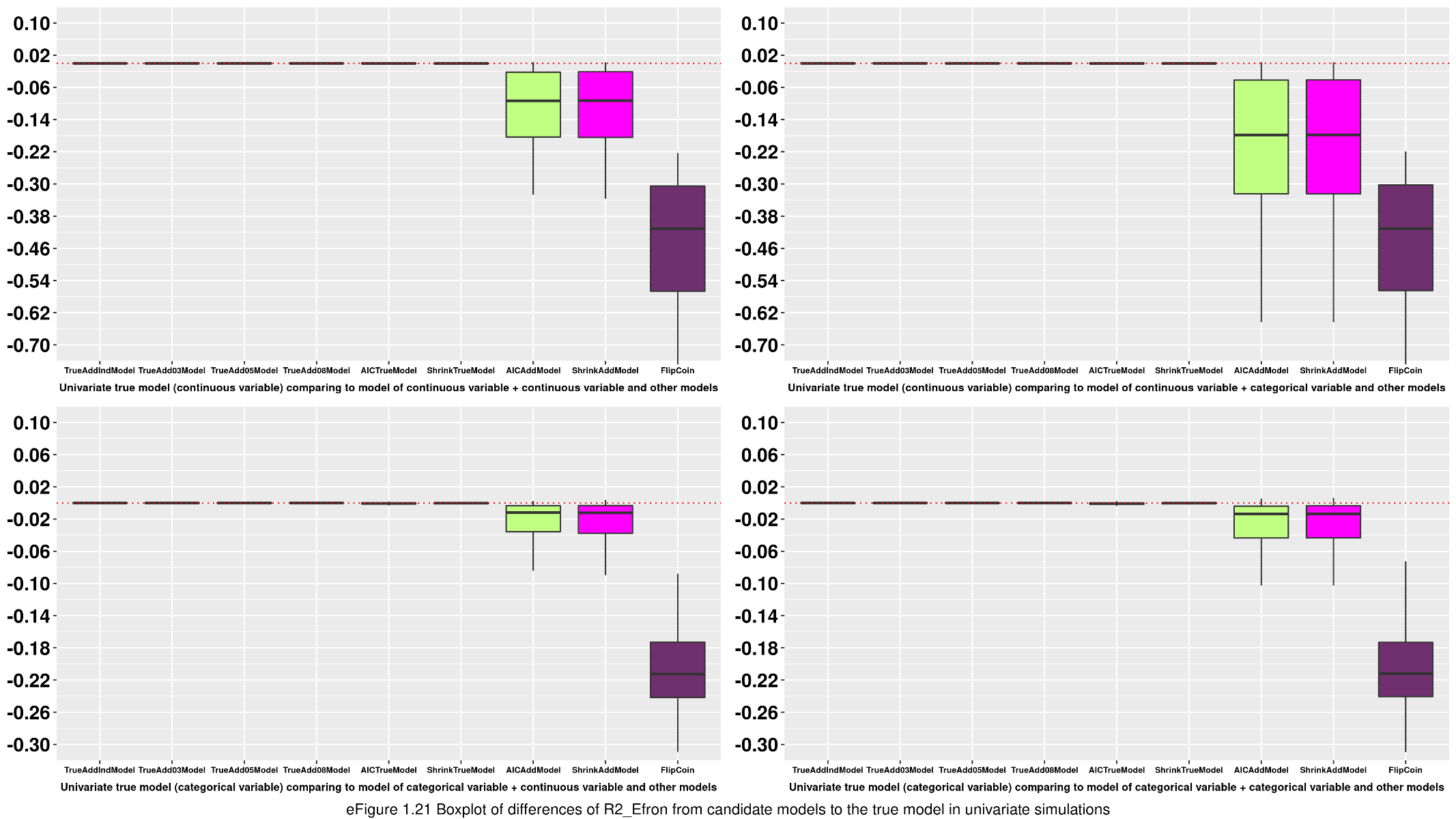

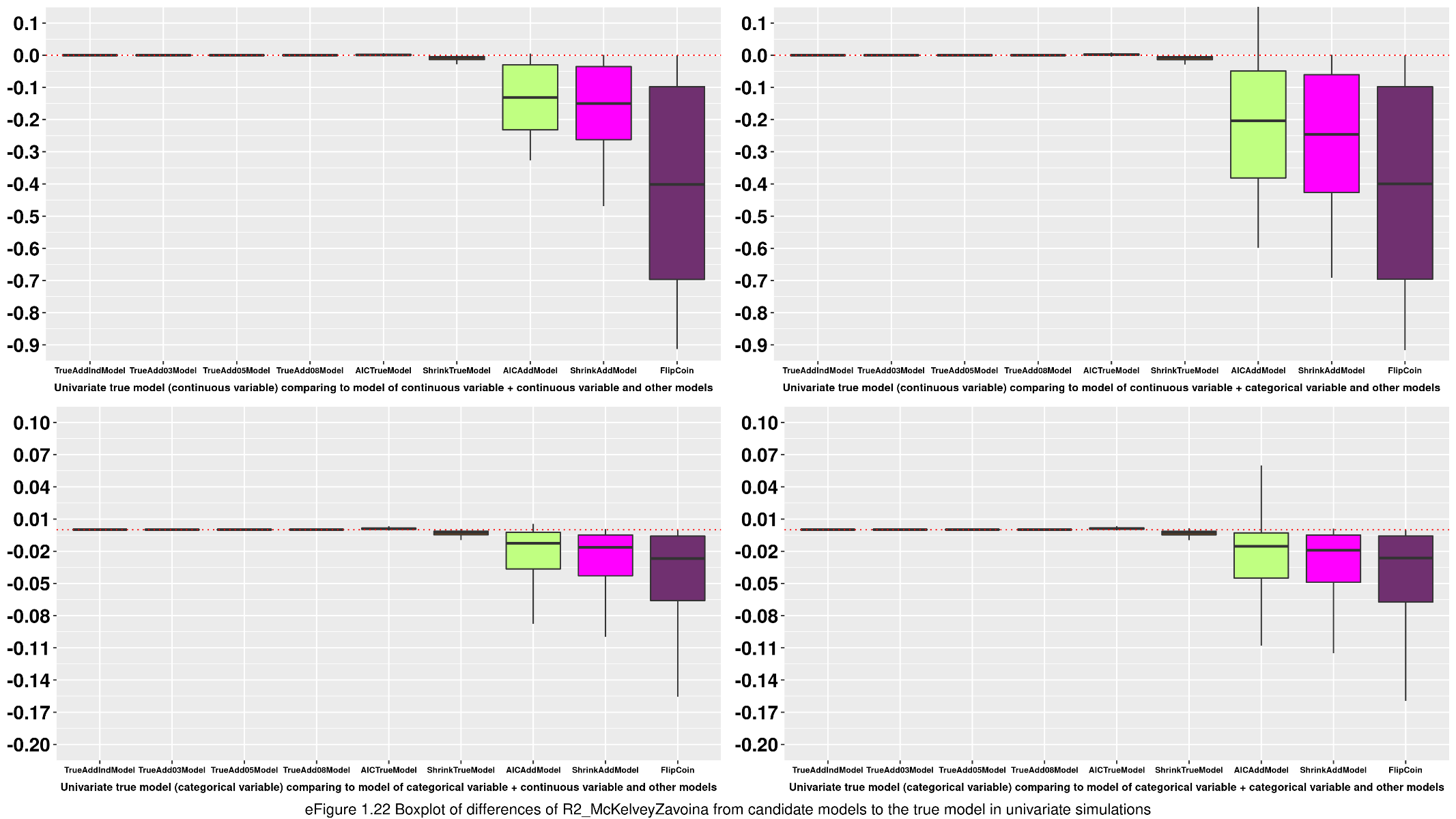

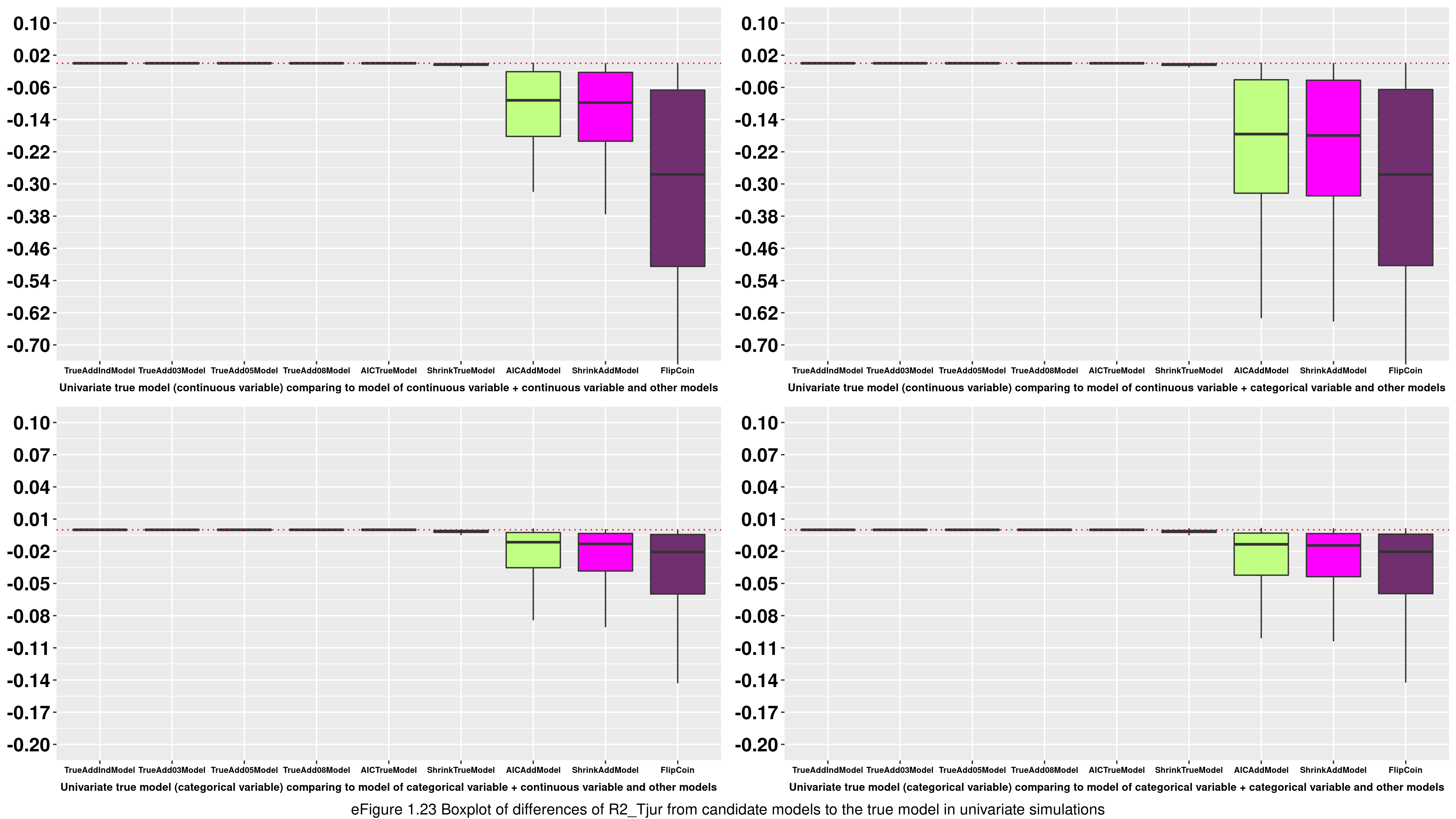

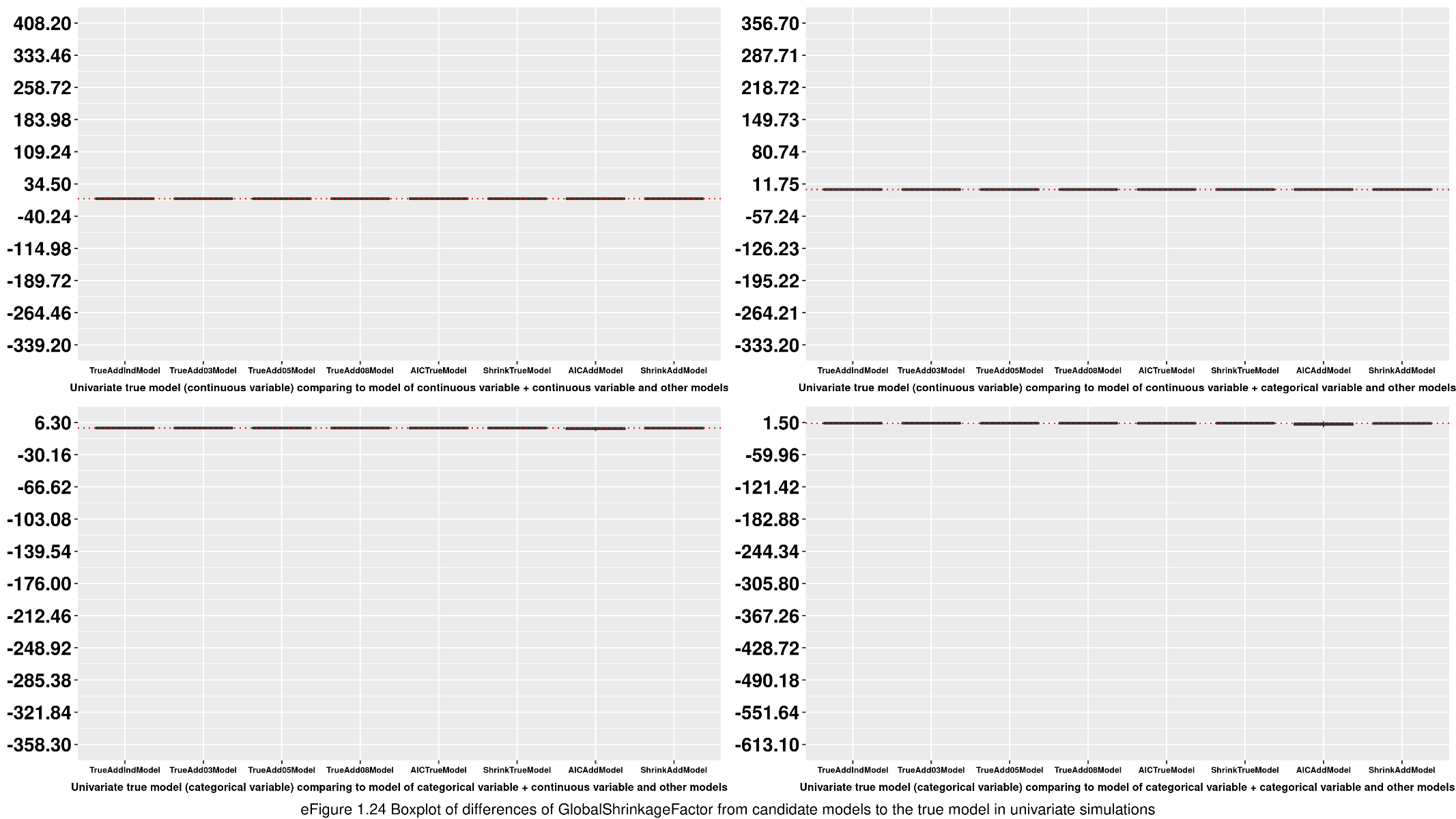

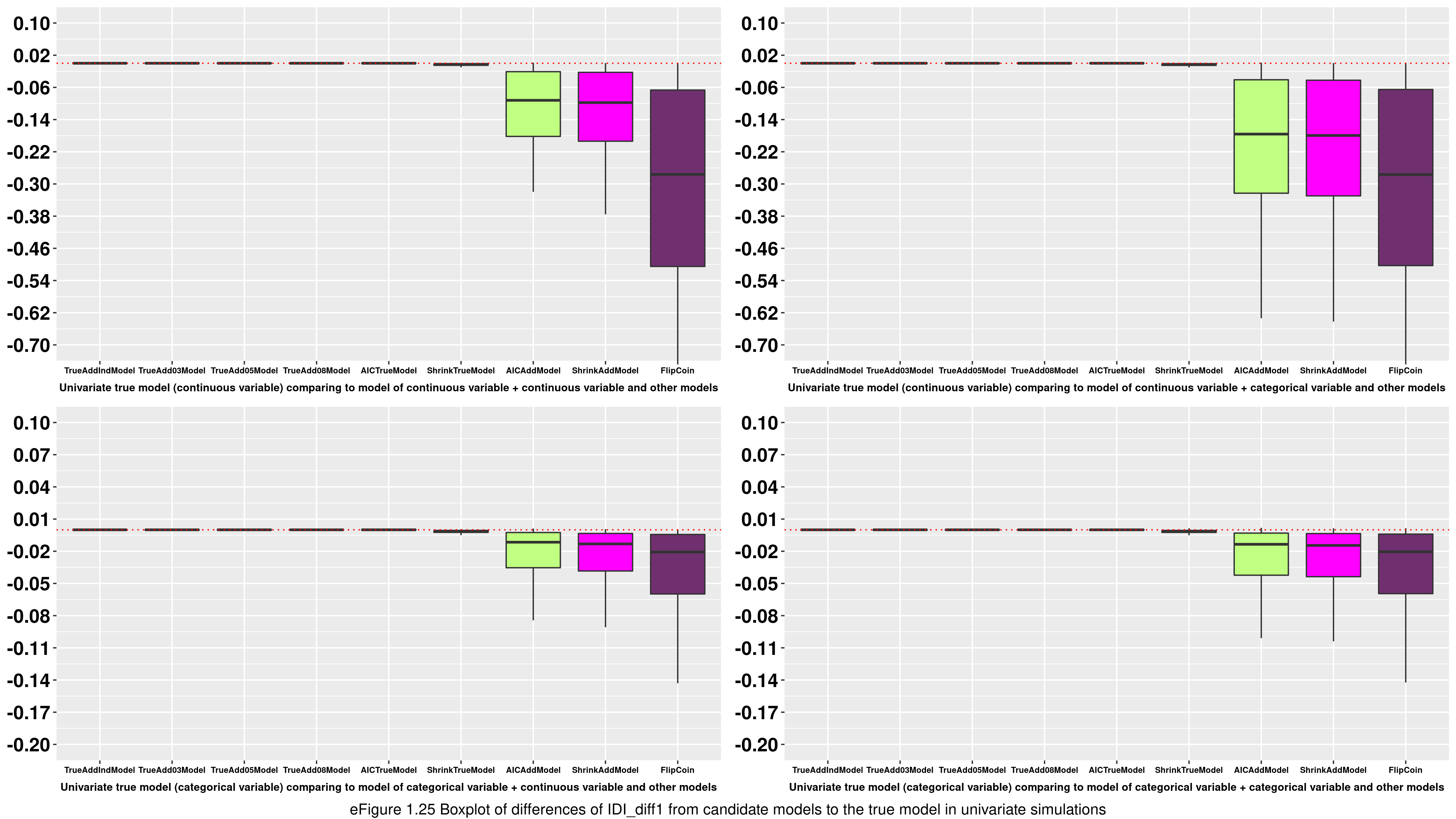


**eFigure 2.2 – 2.25**


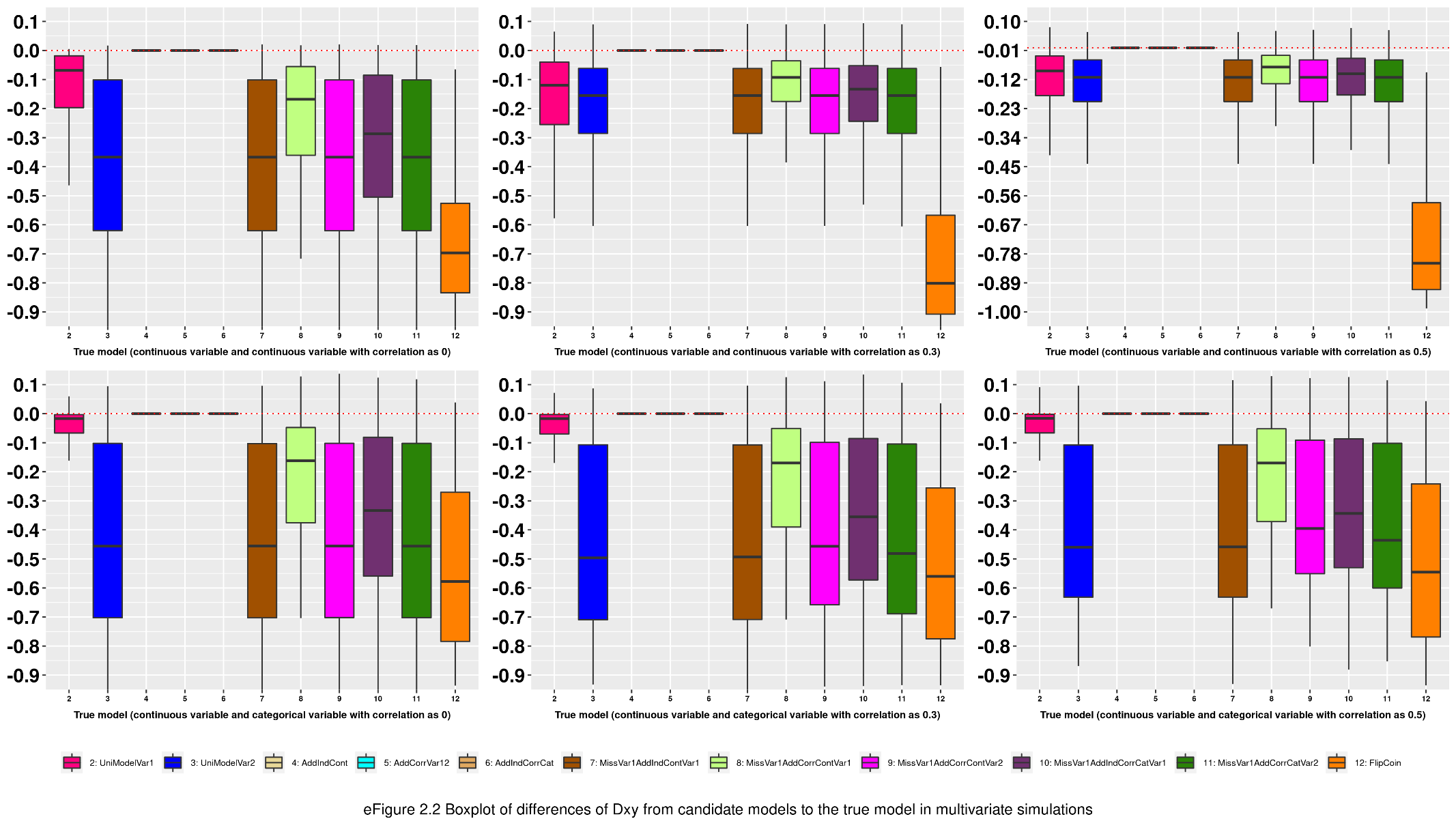

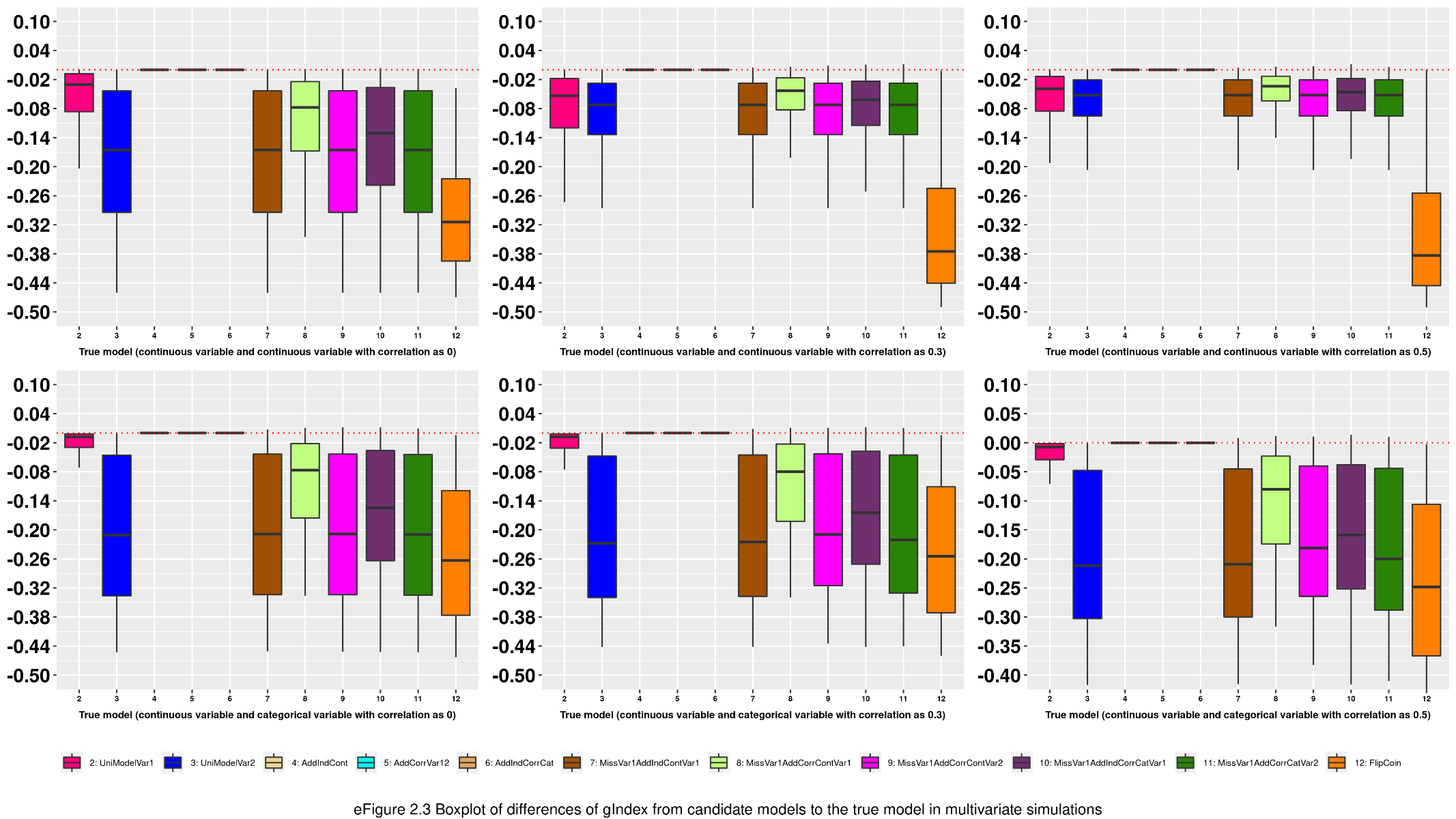

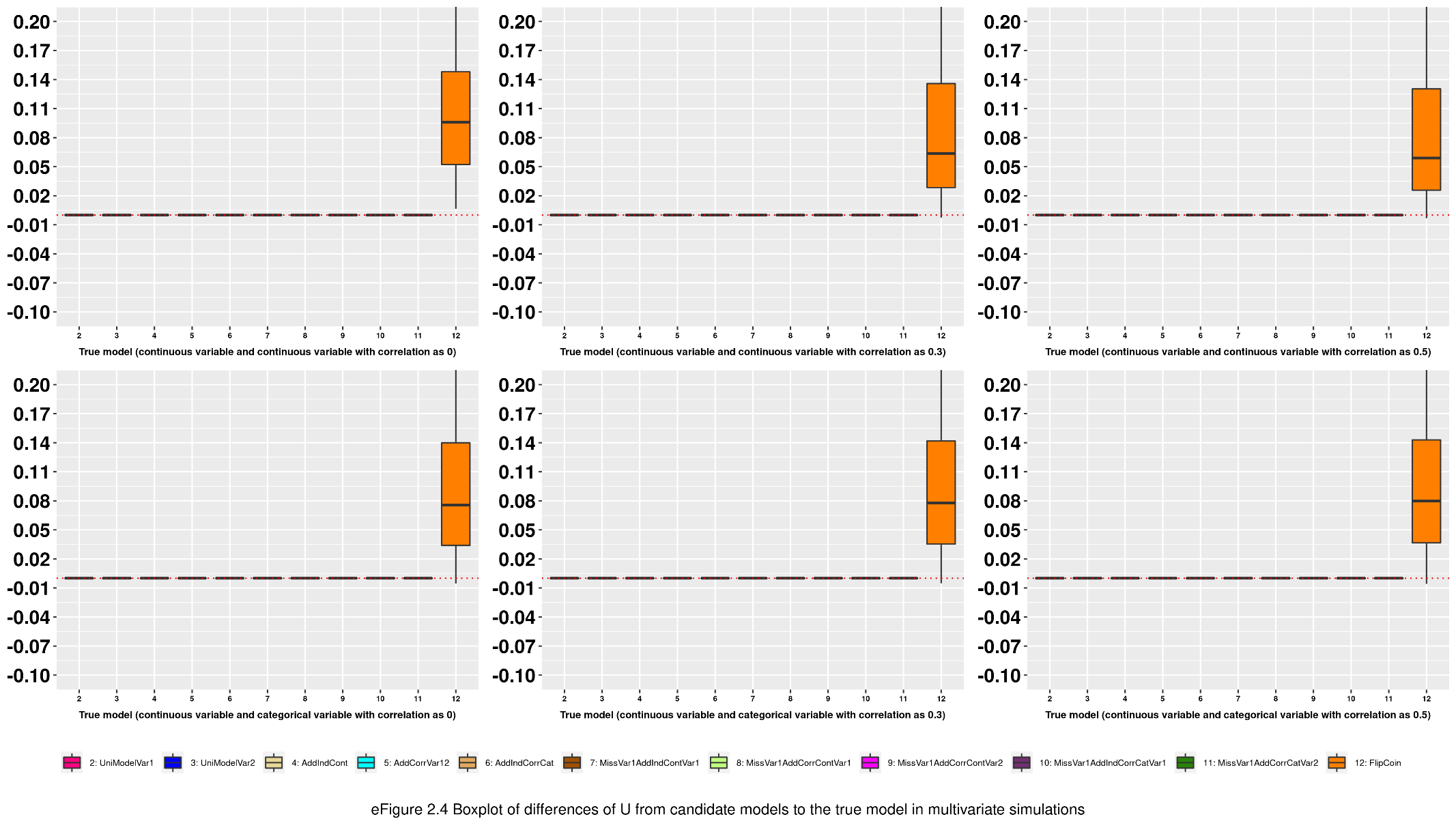

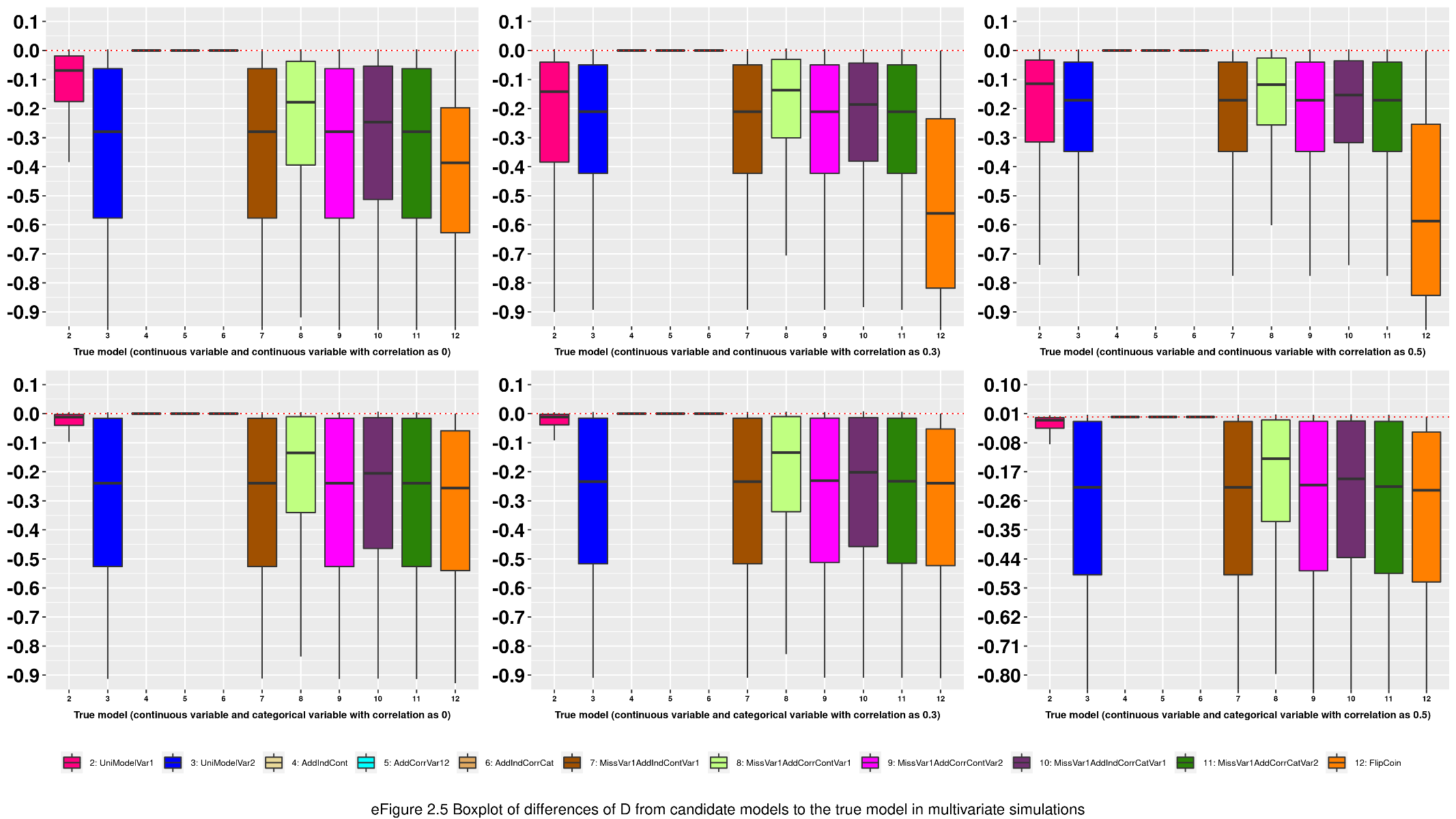

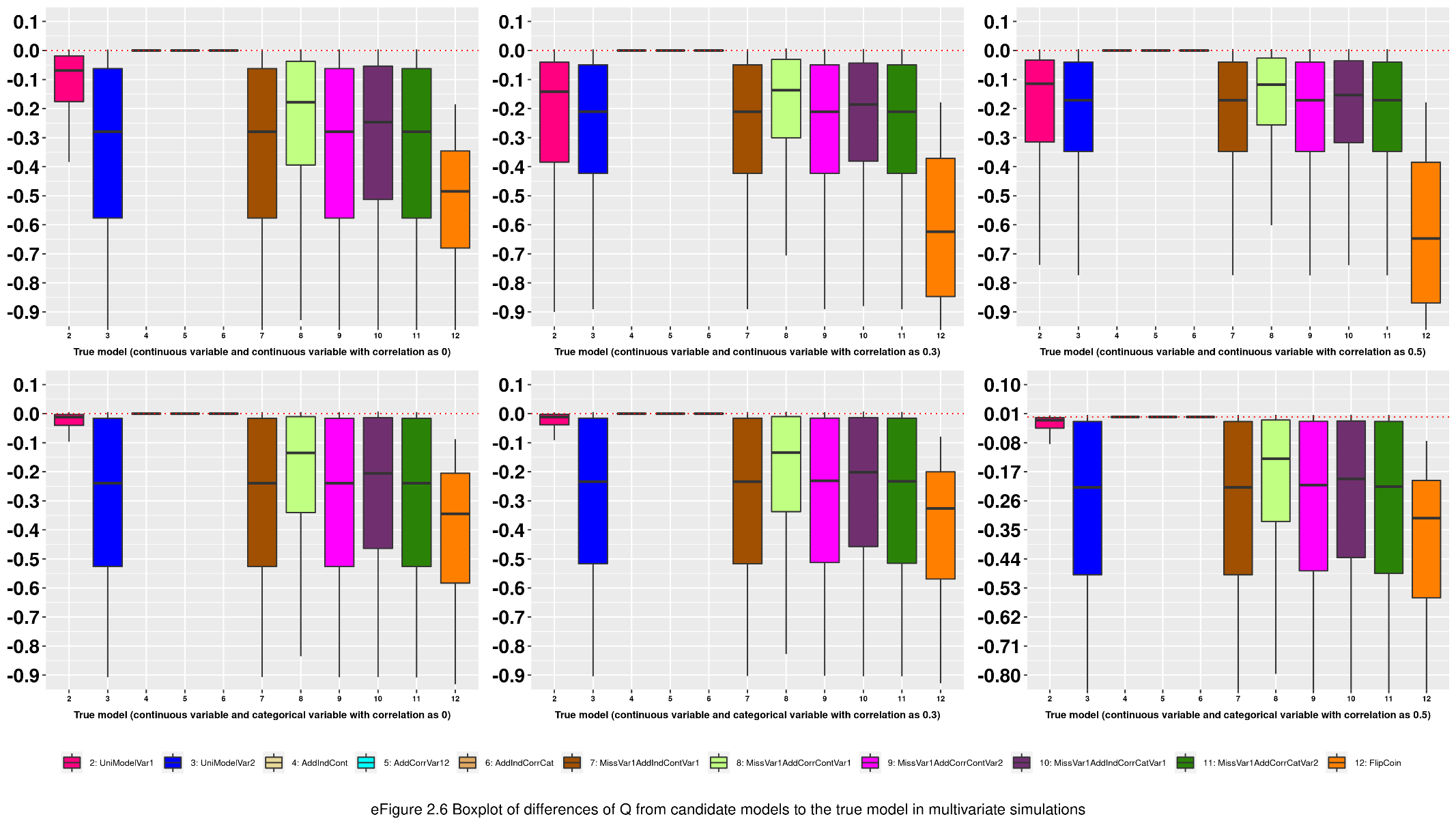

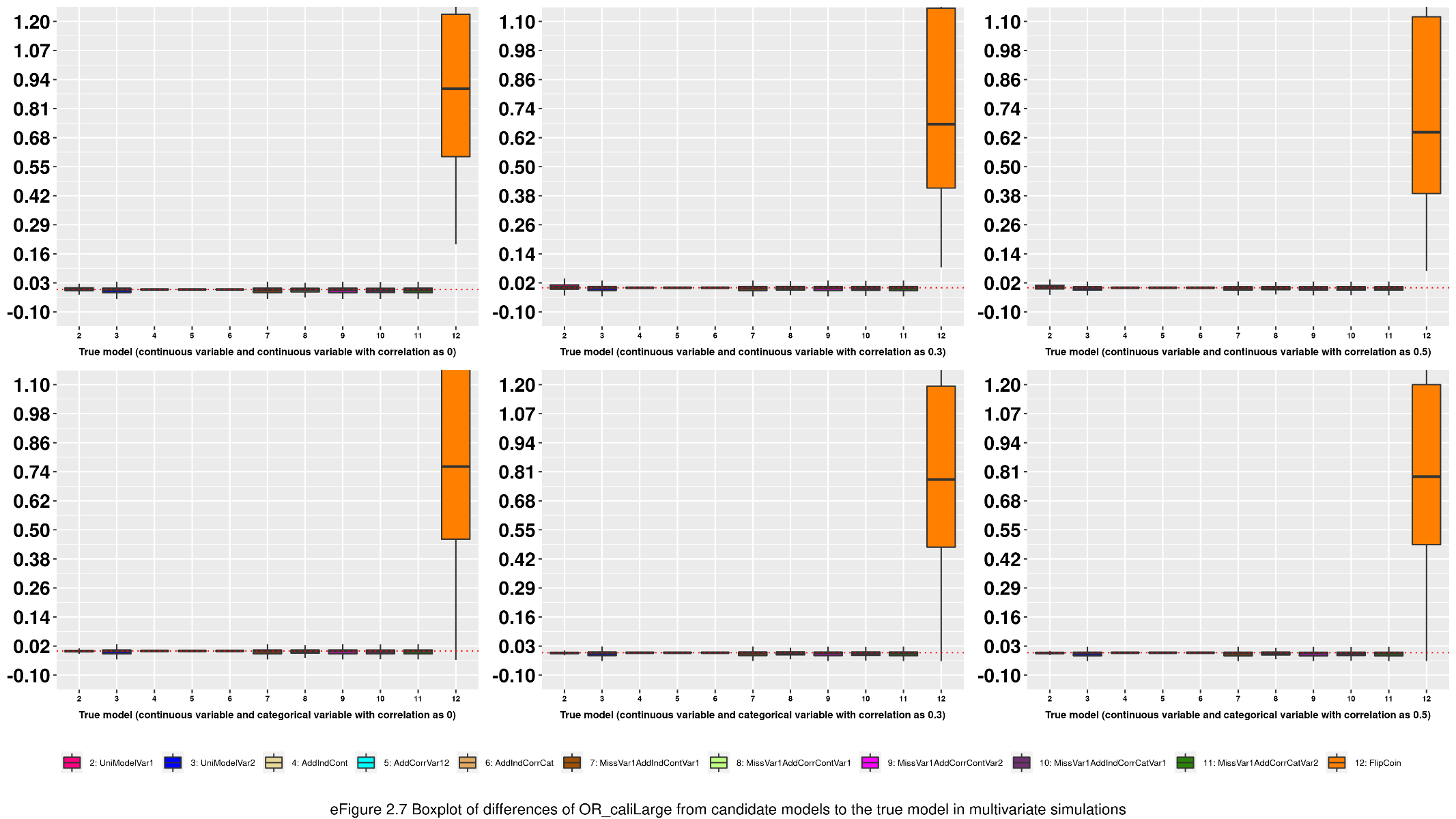

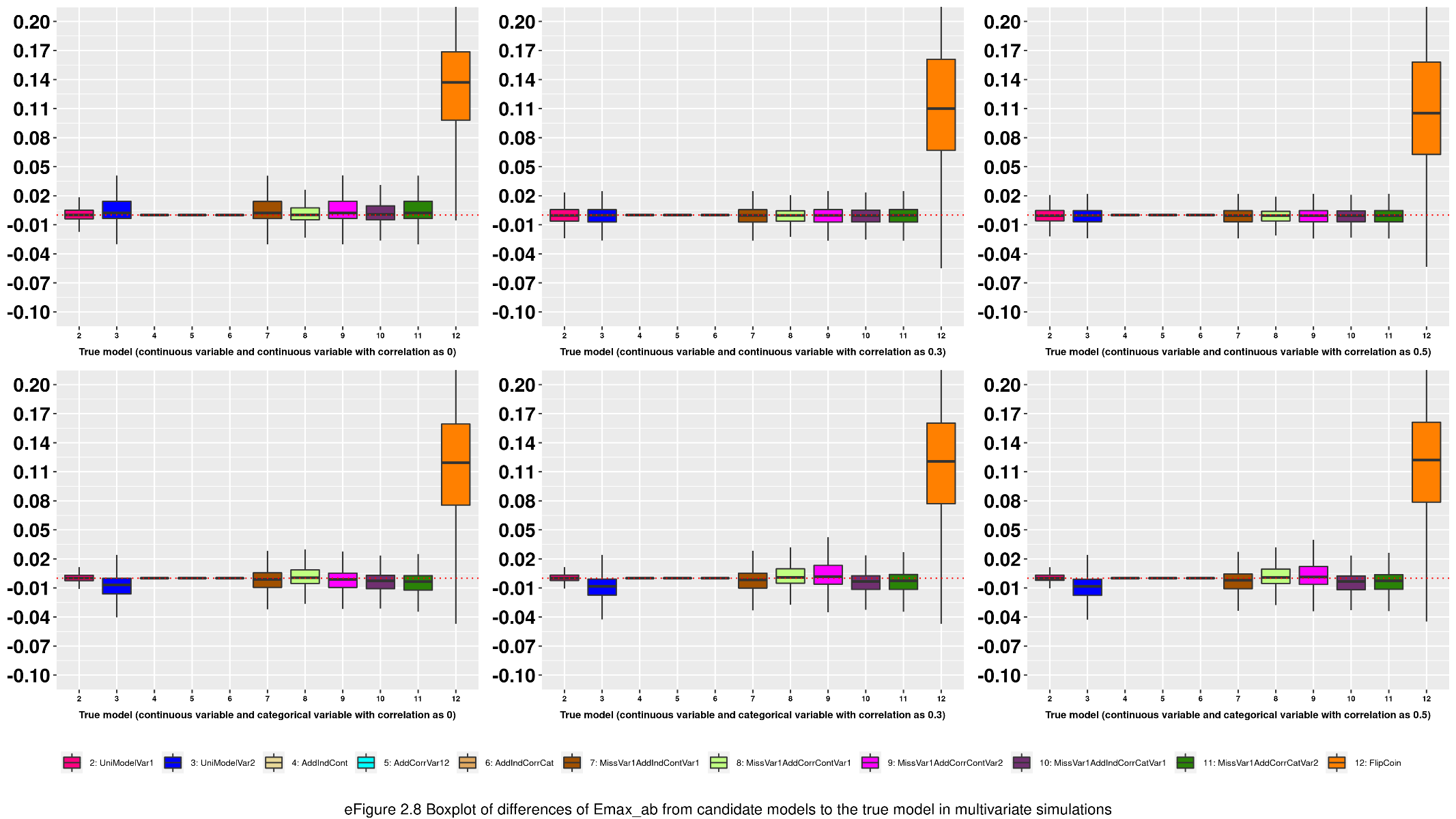

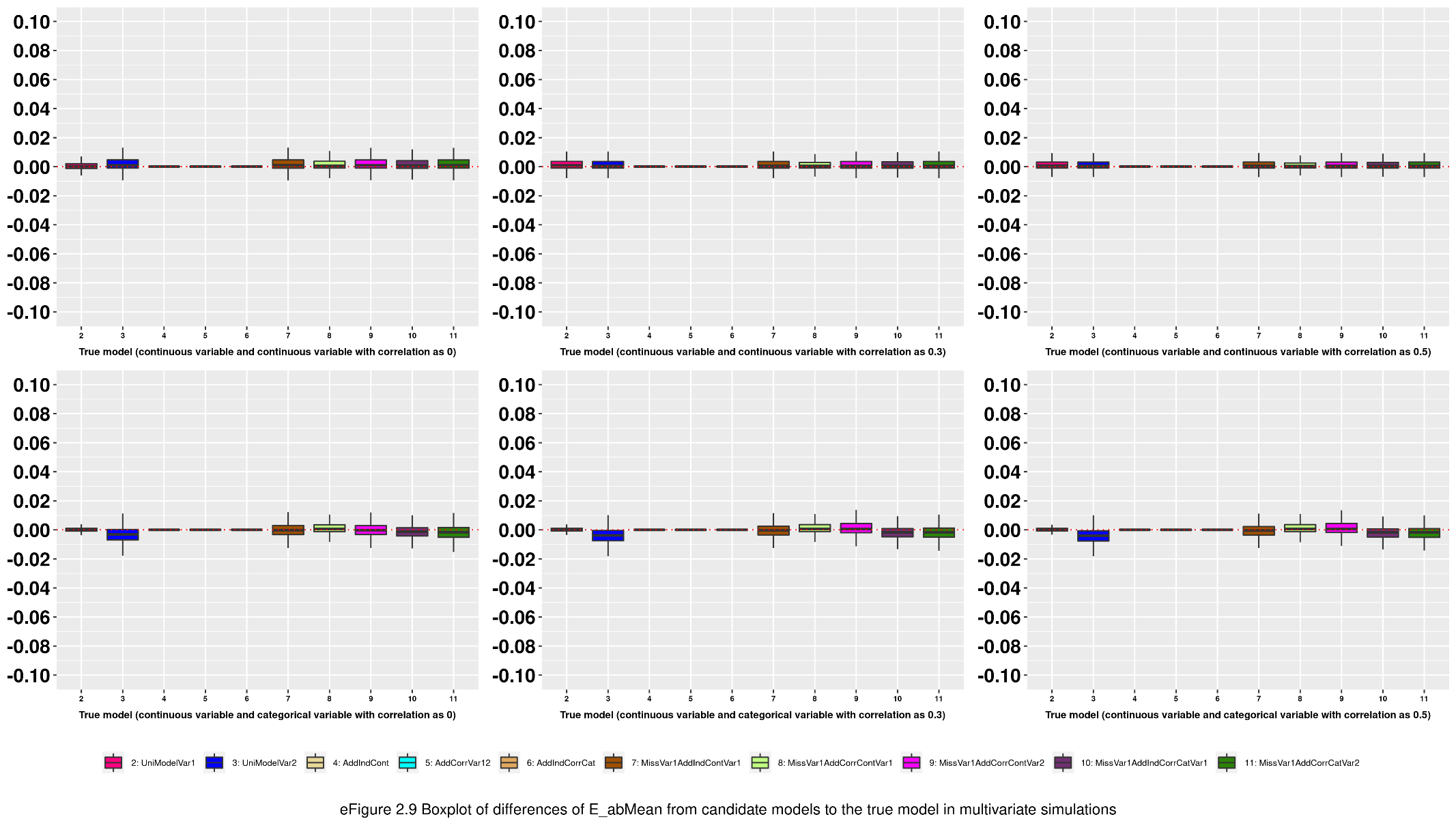

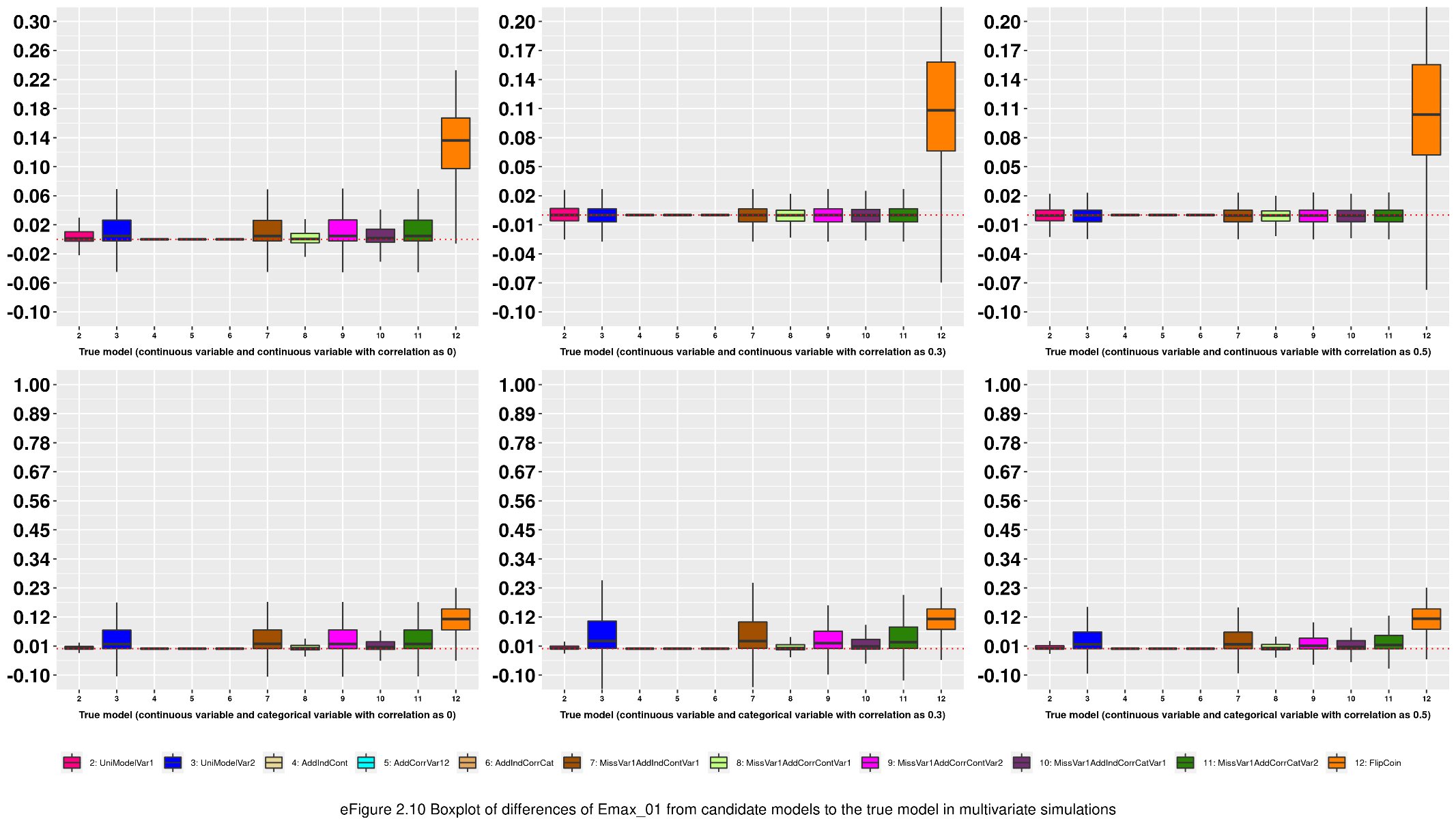

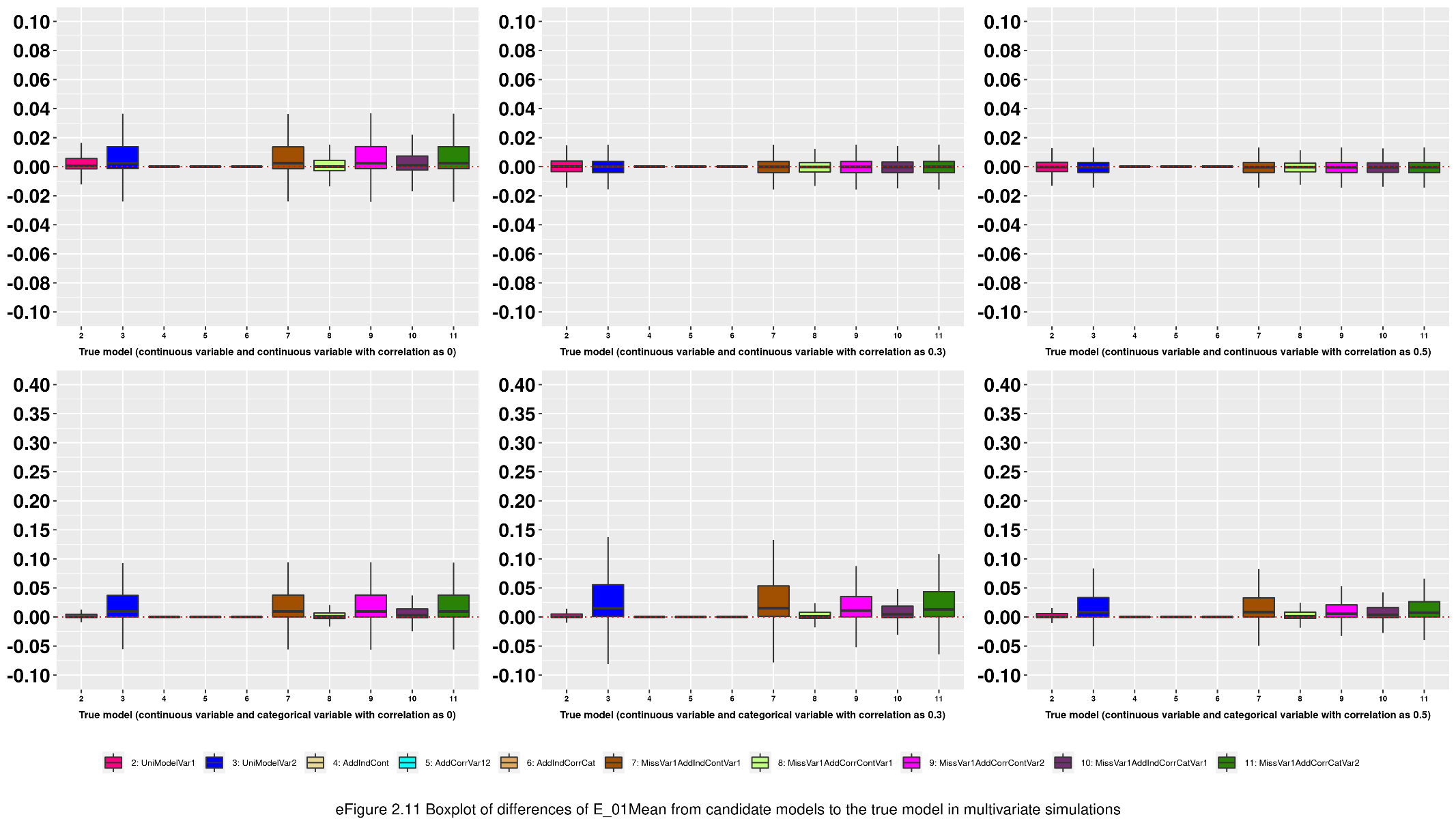

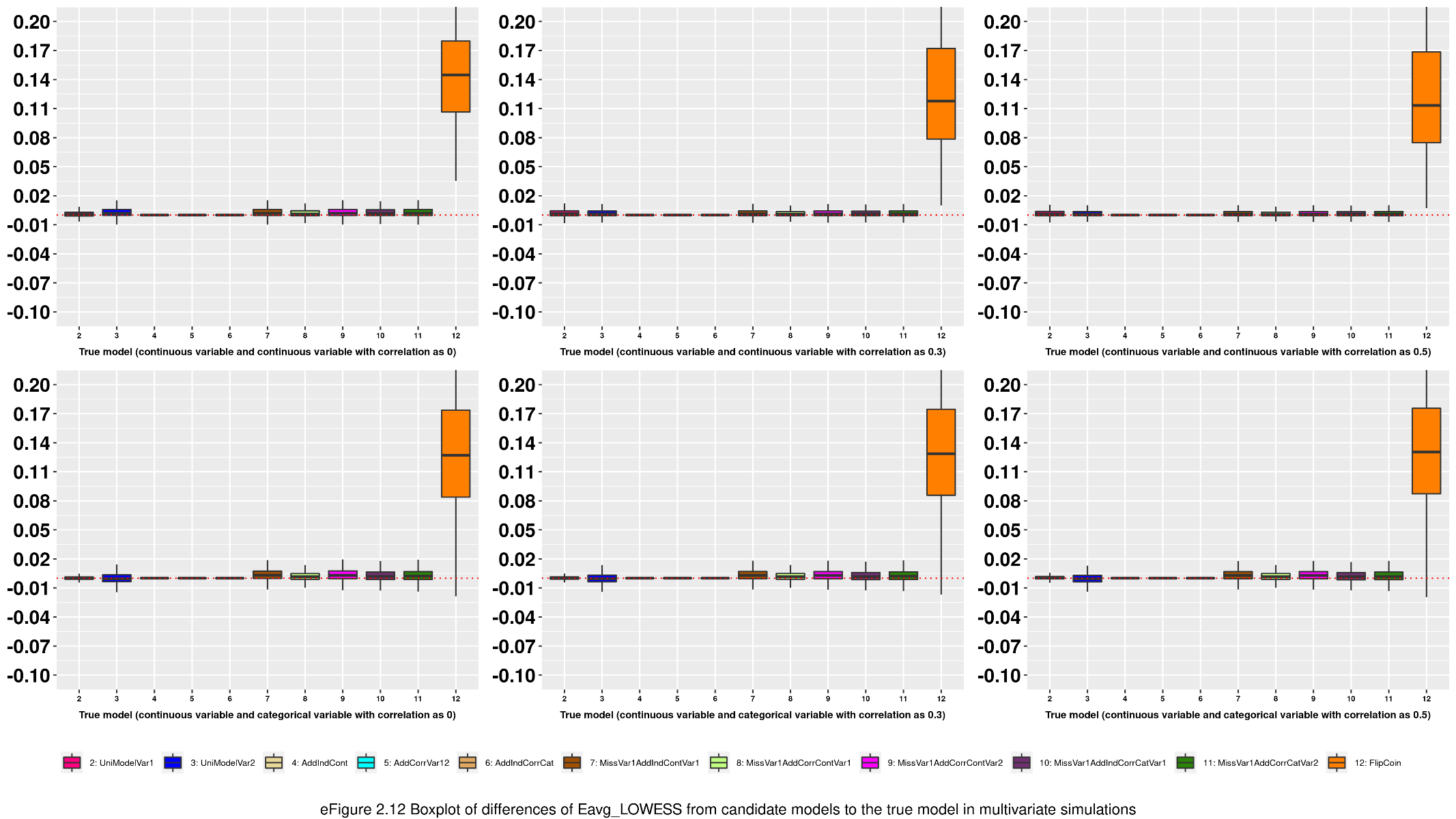

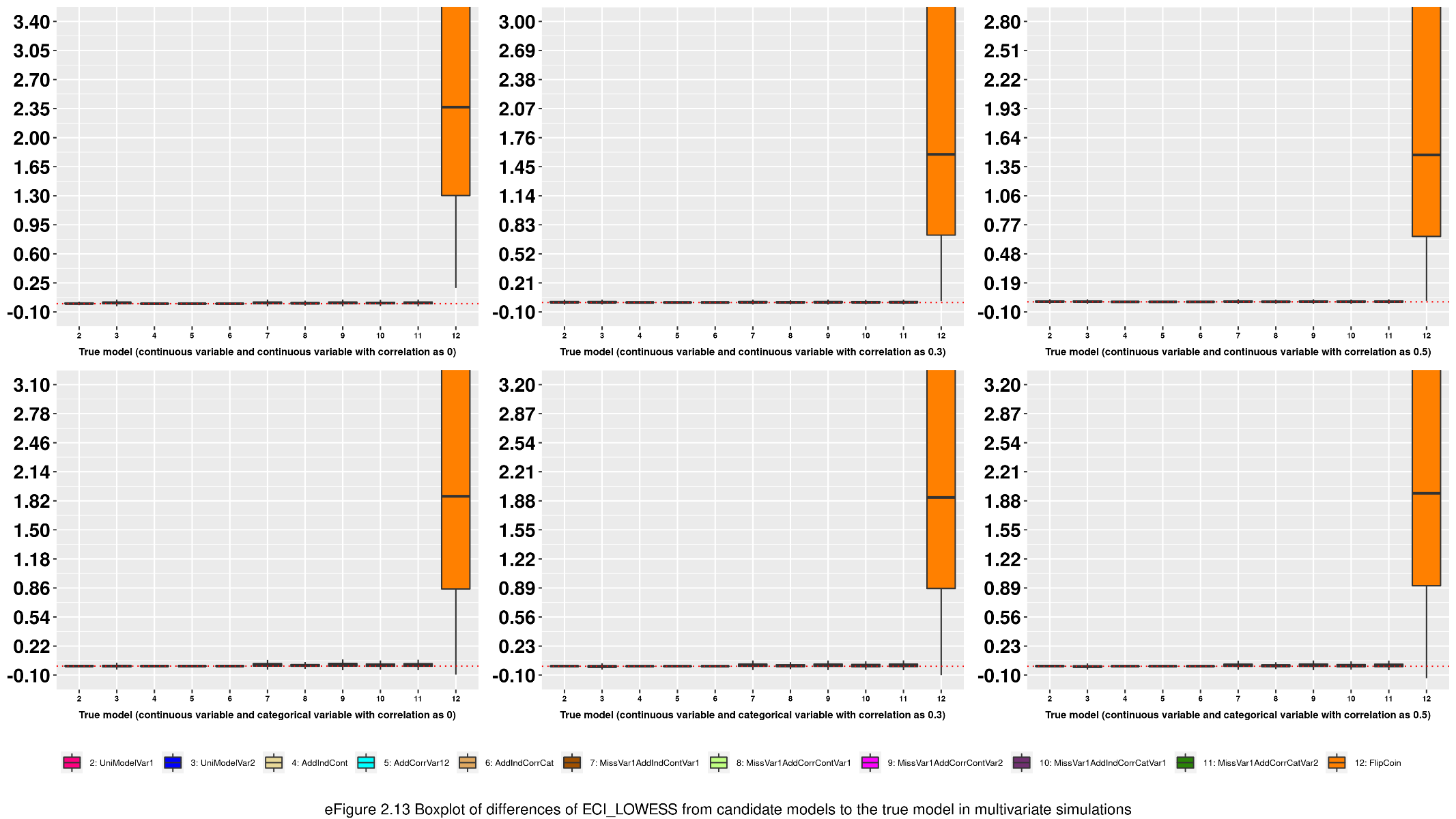

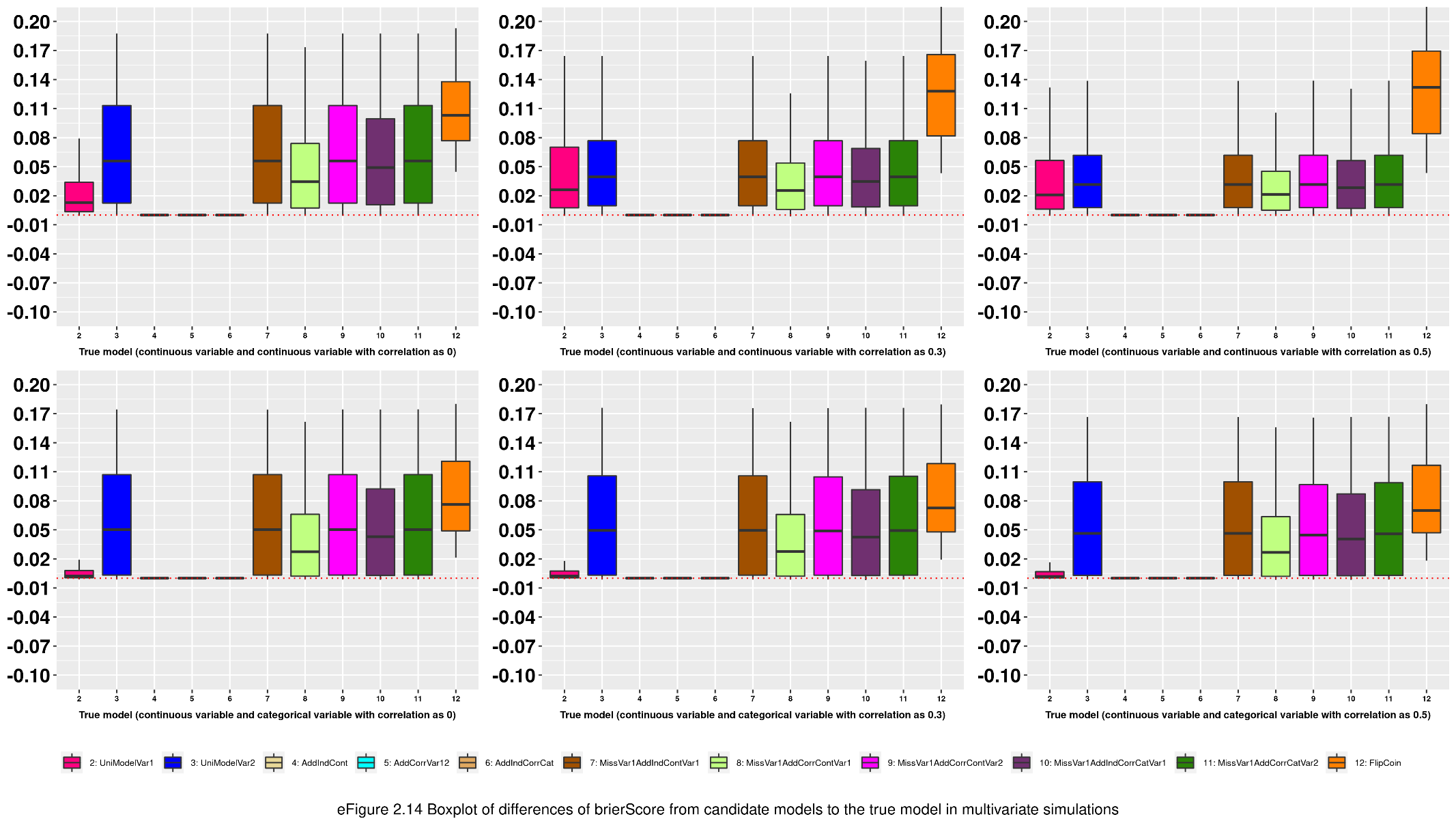

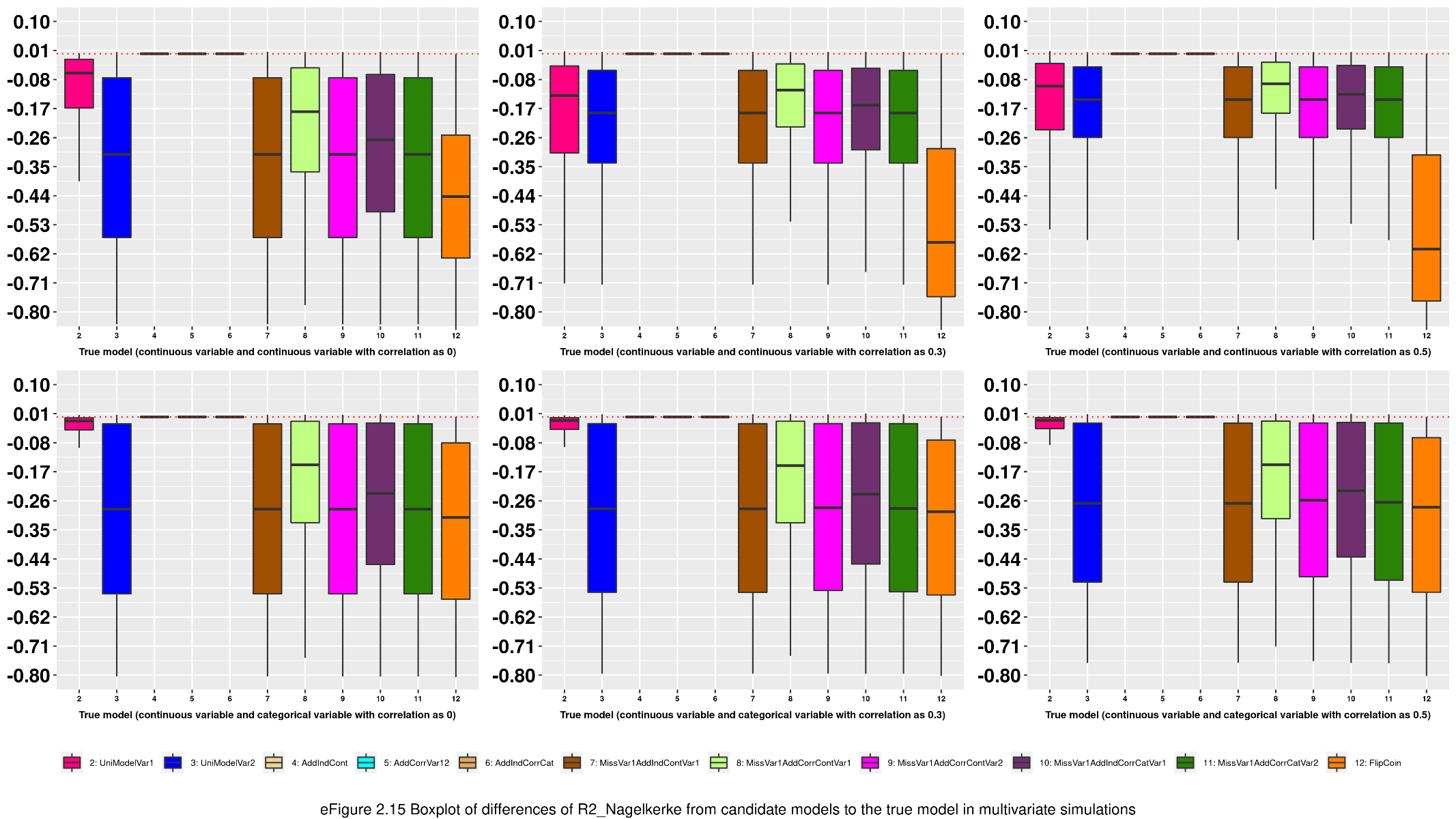

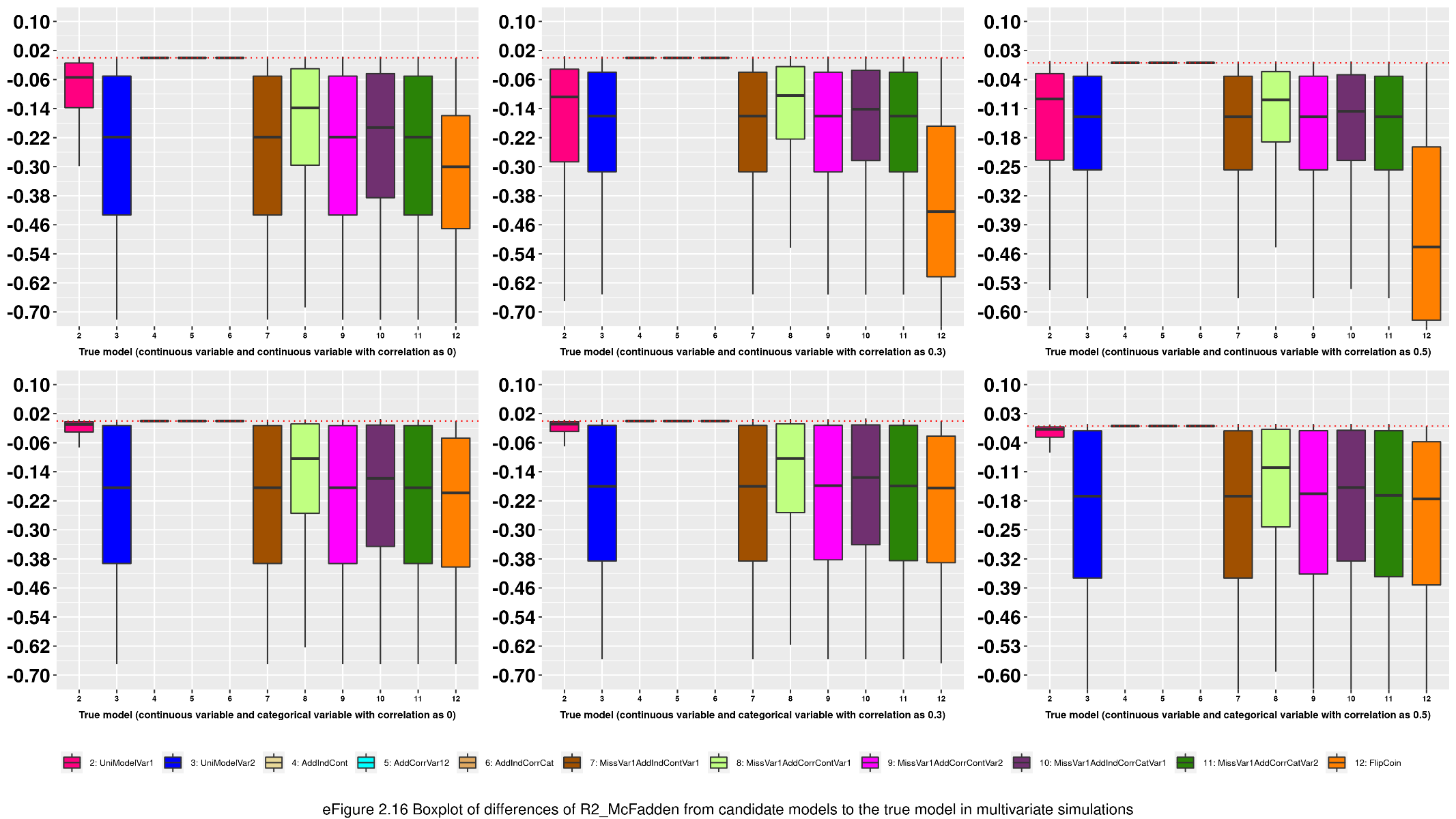

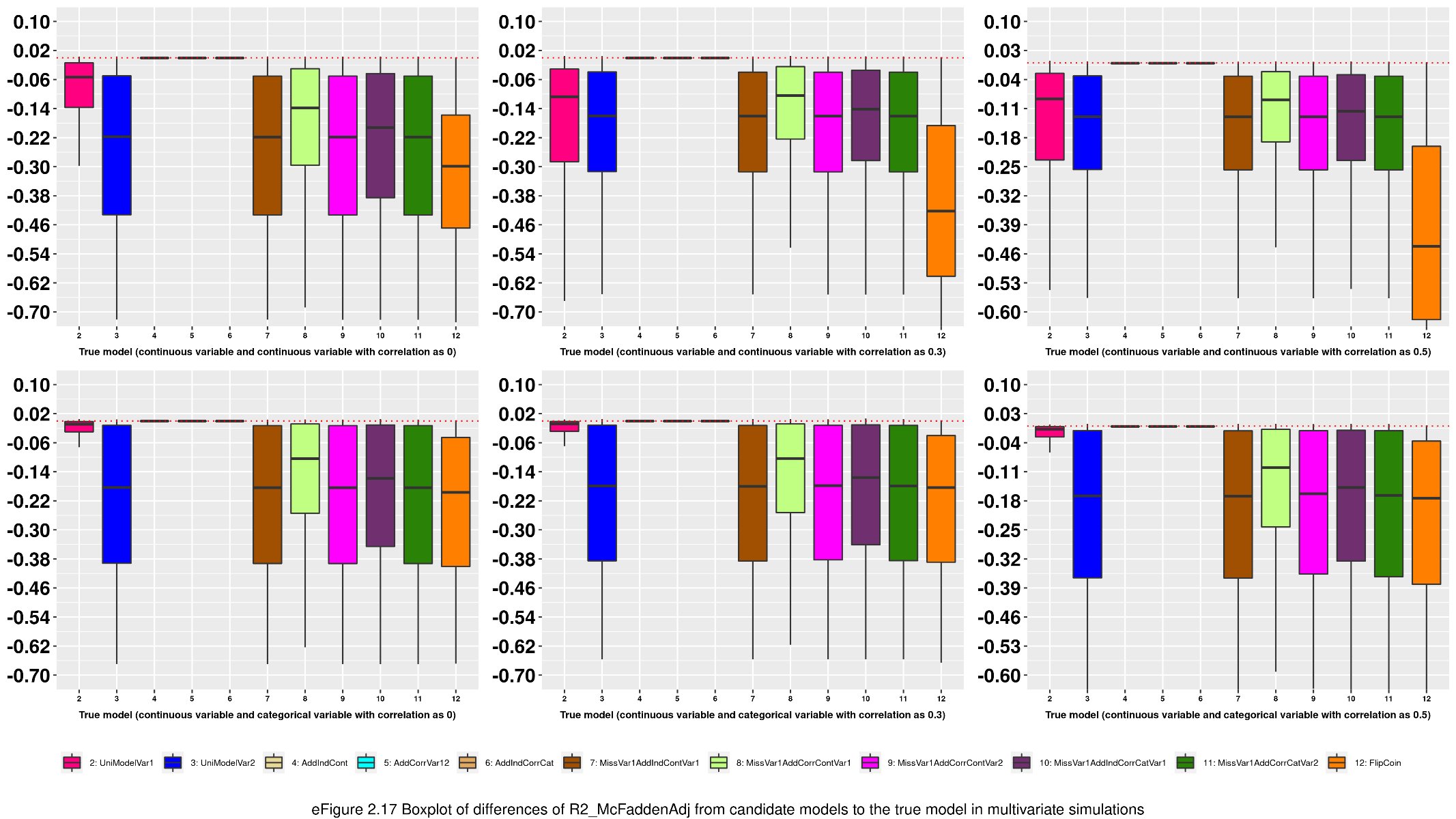

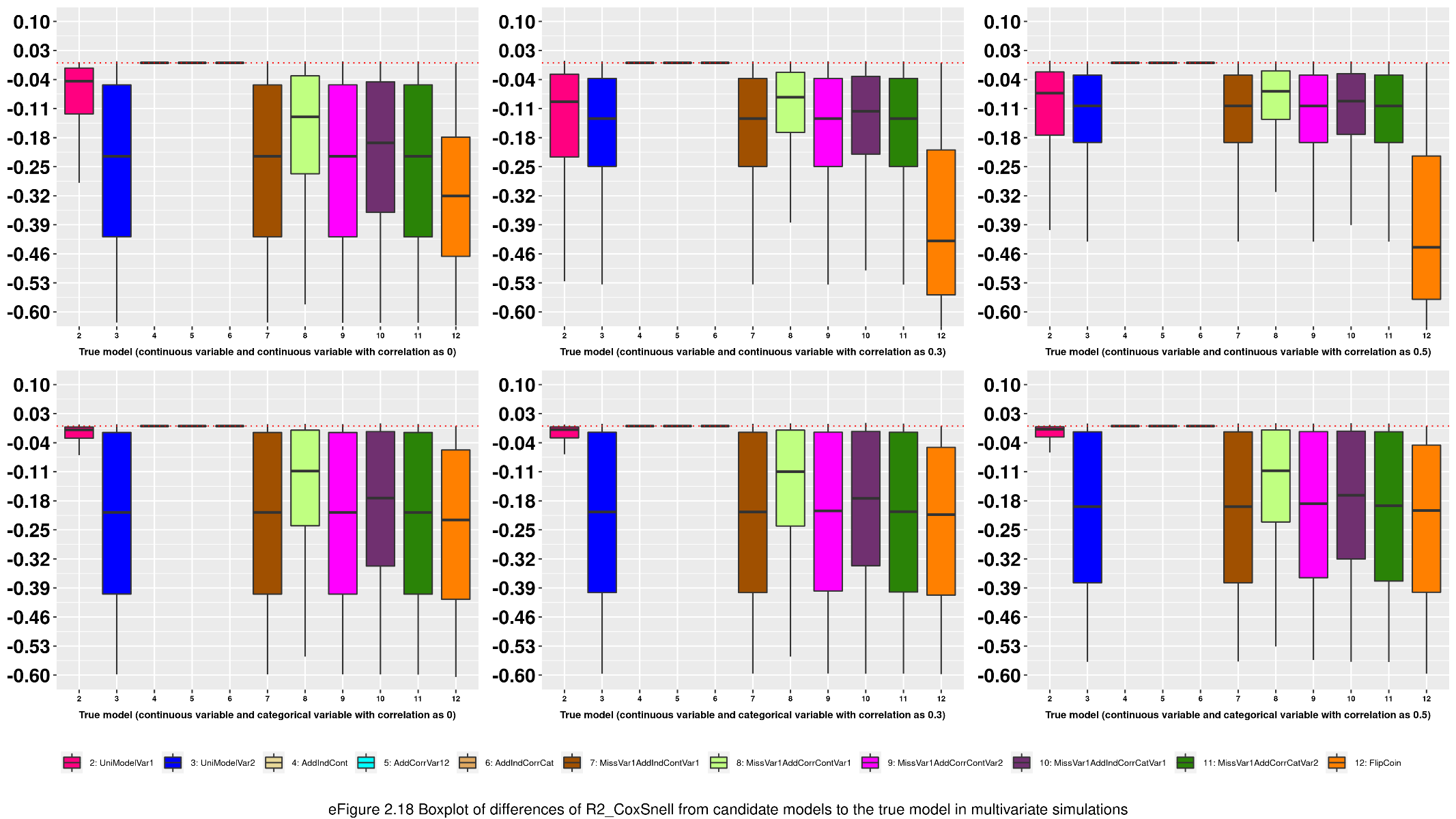

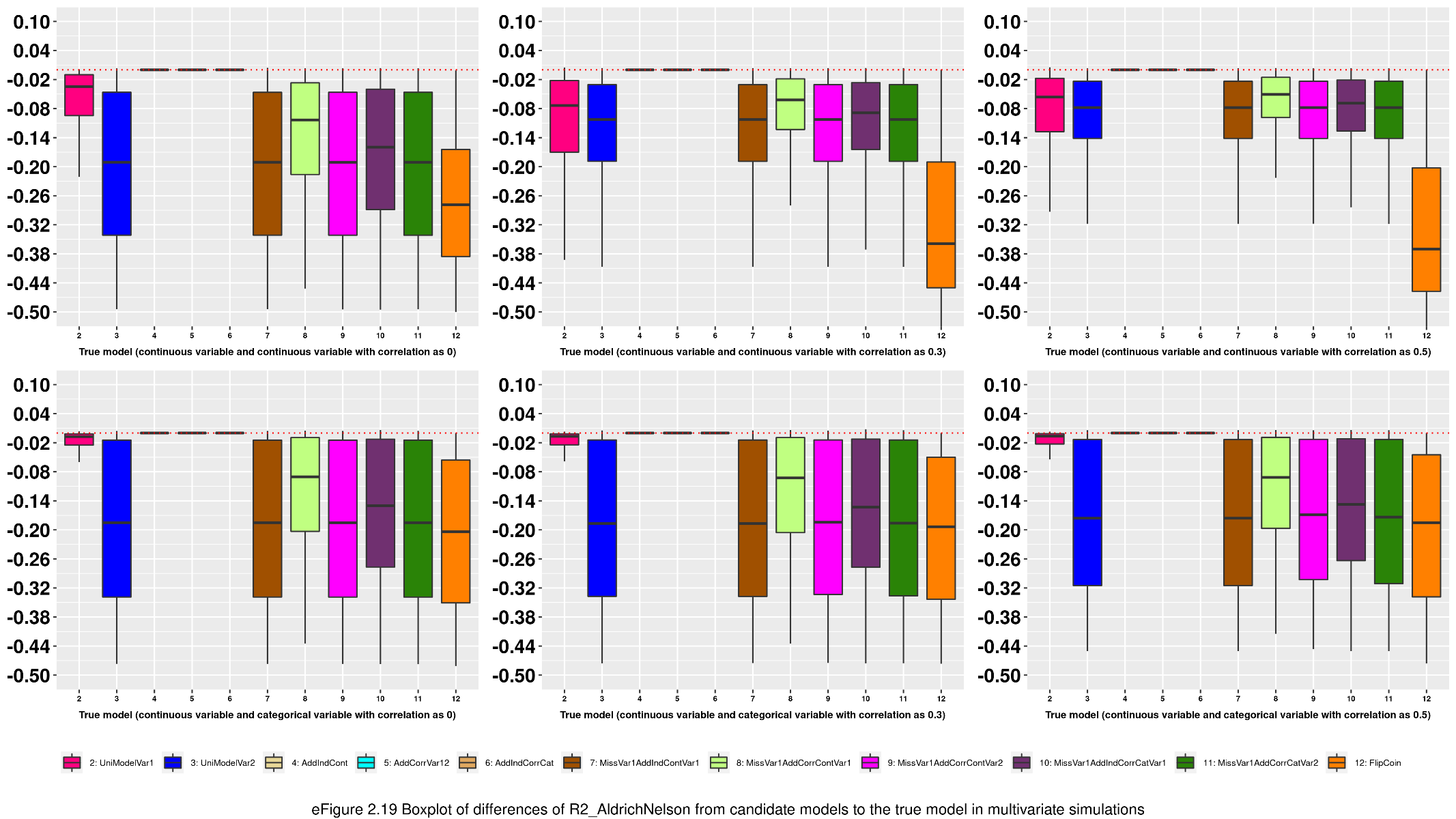

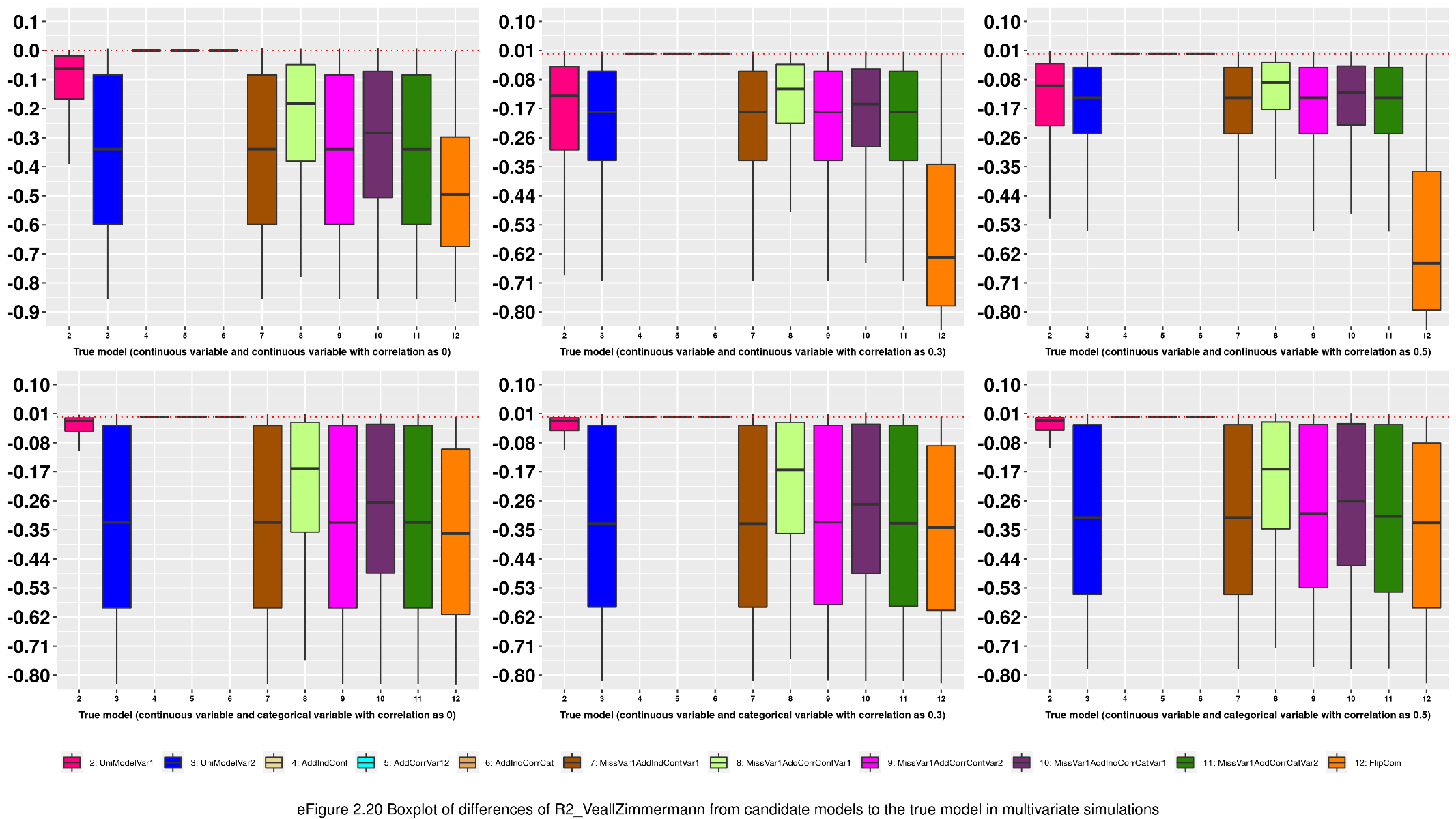

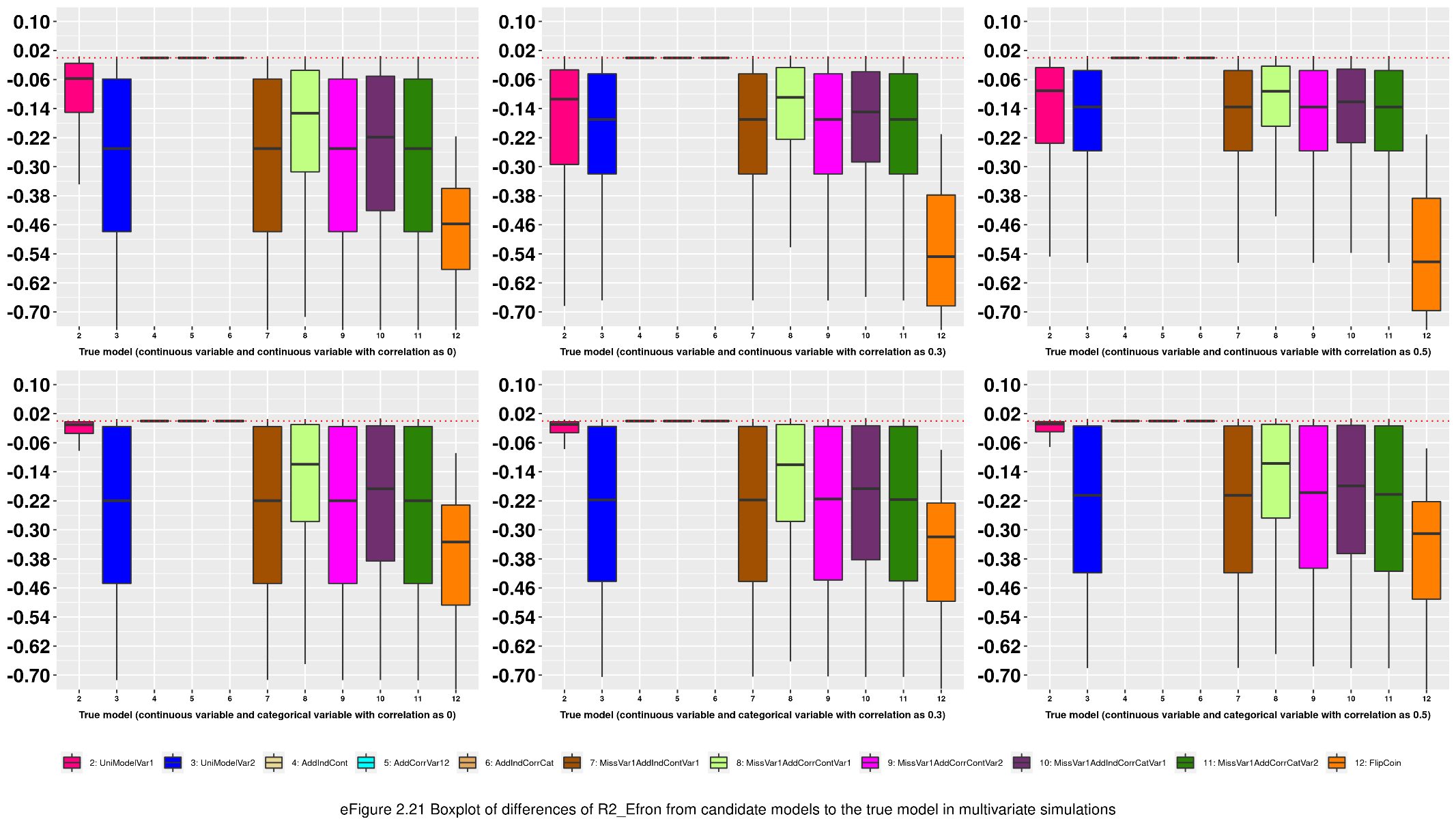

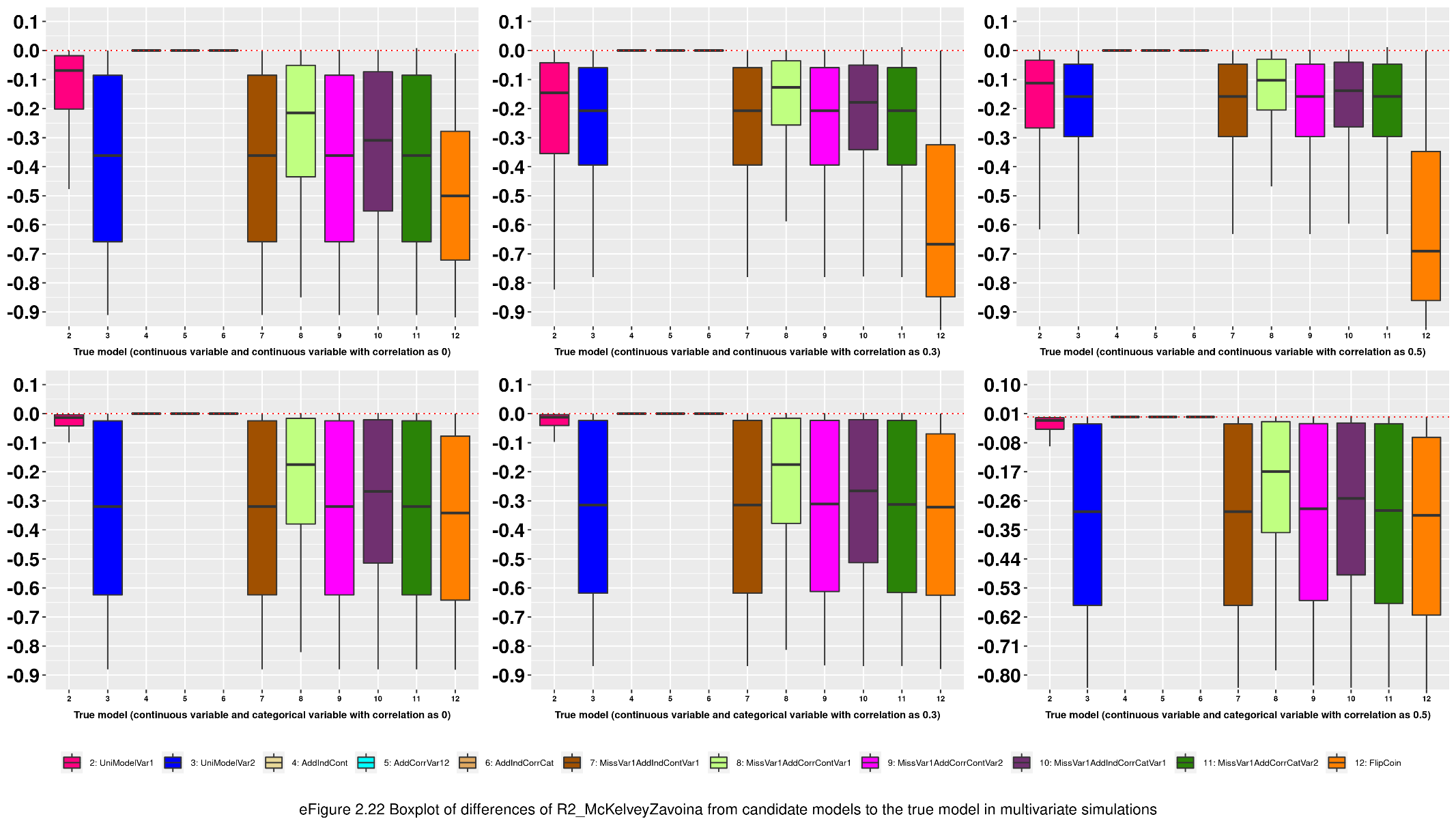

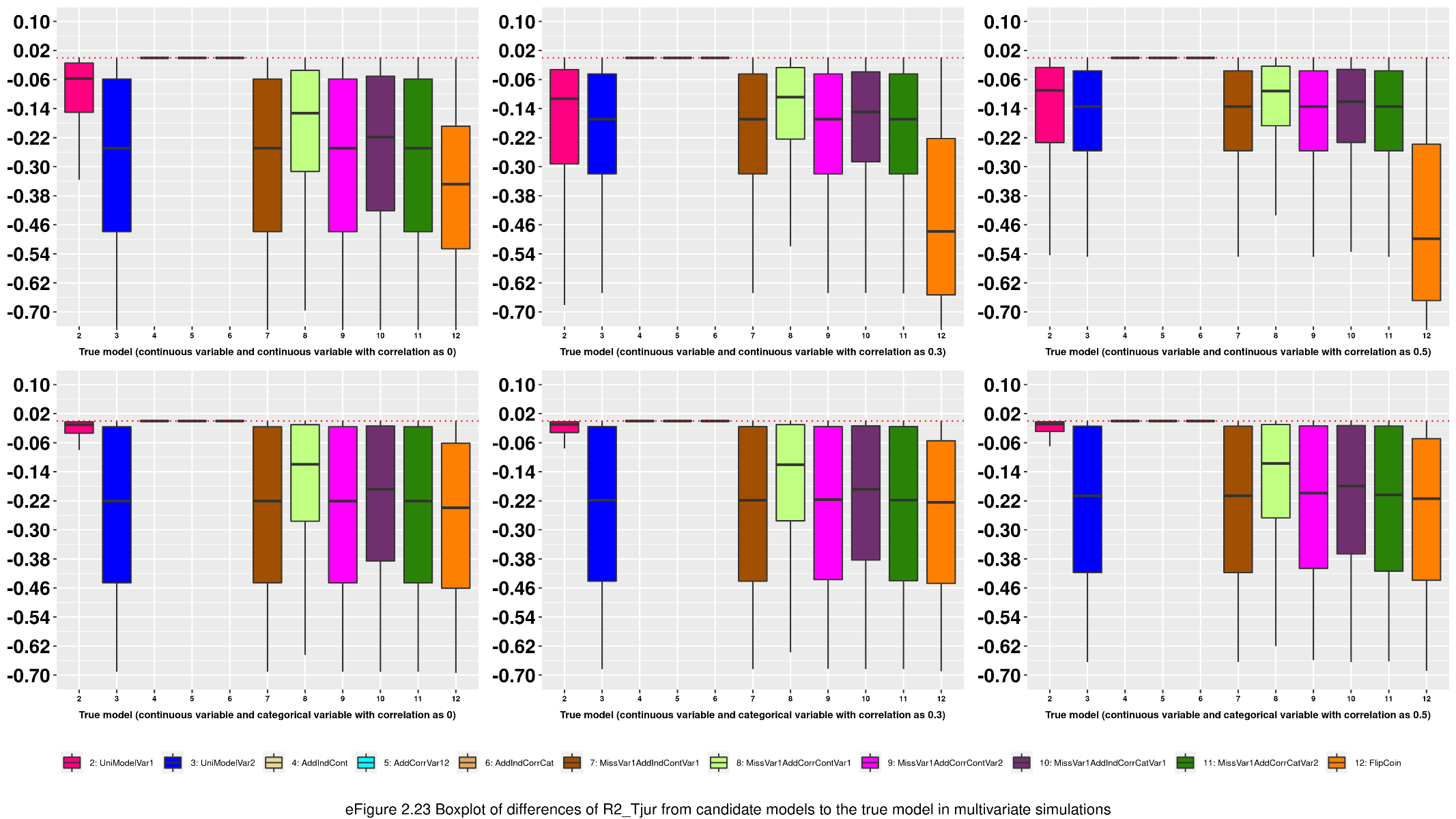

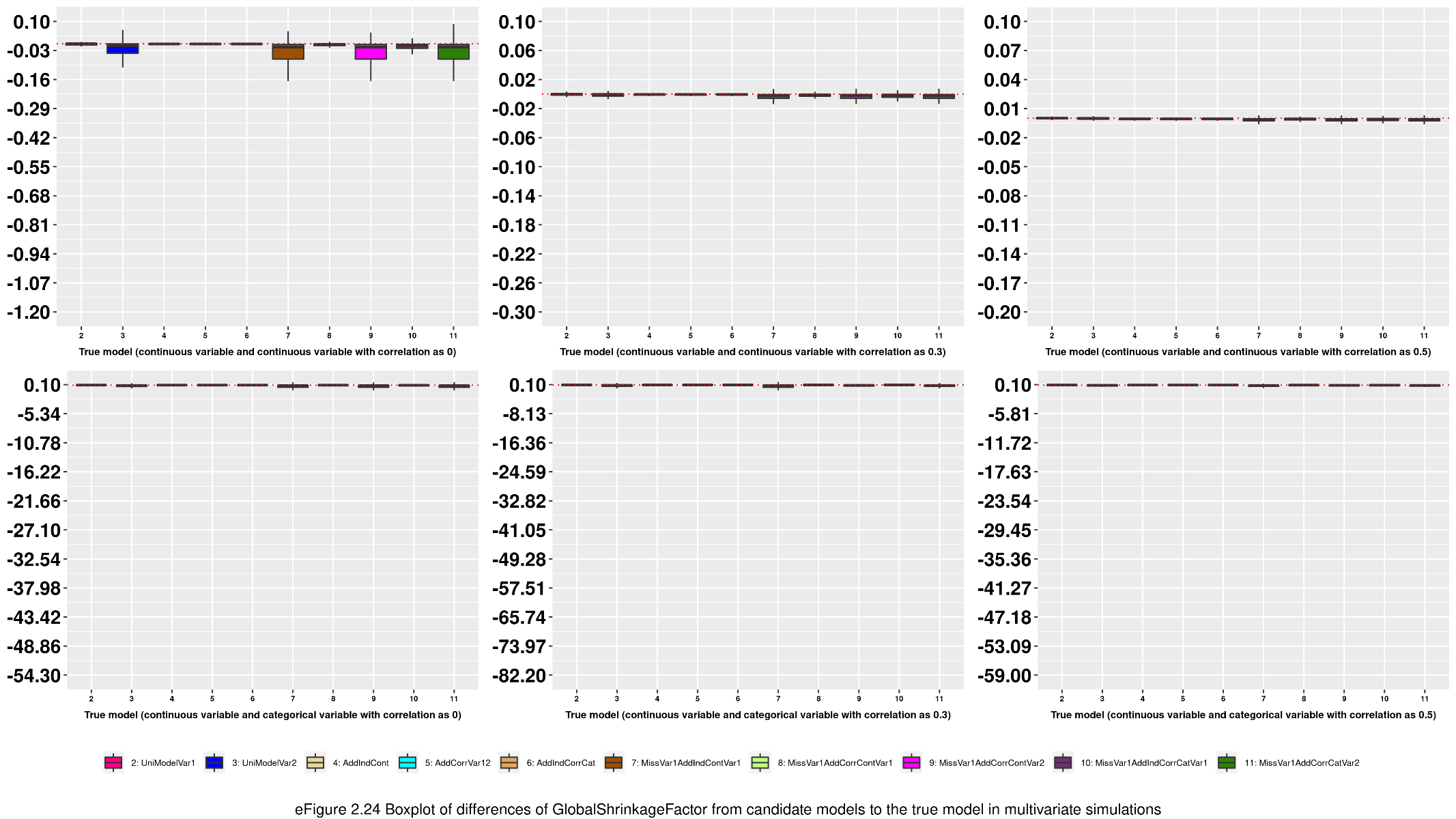

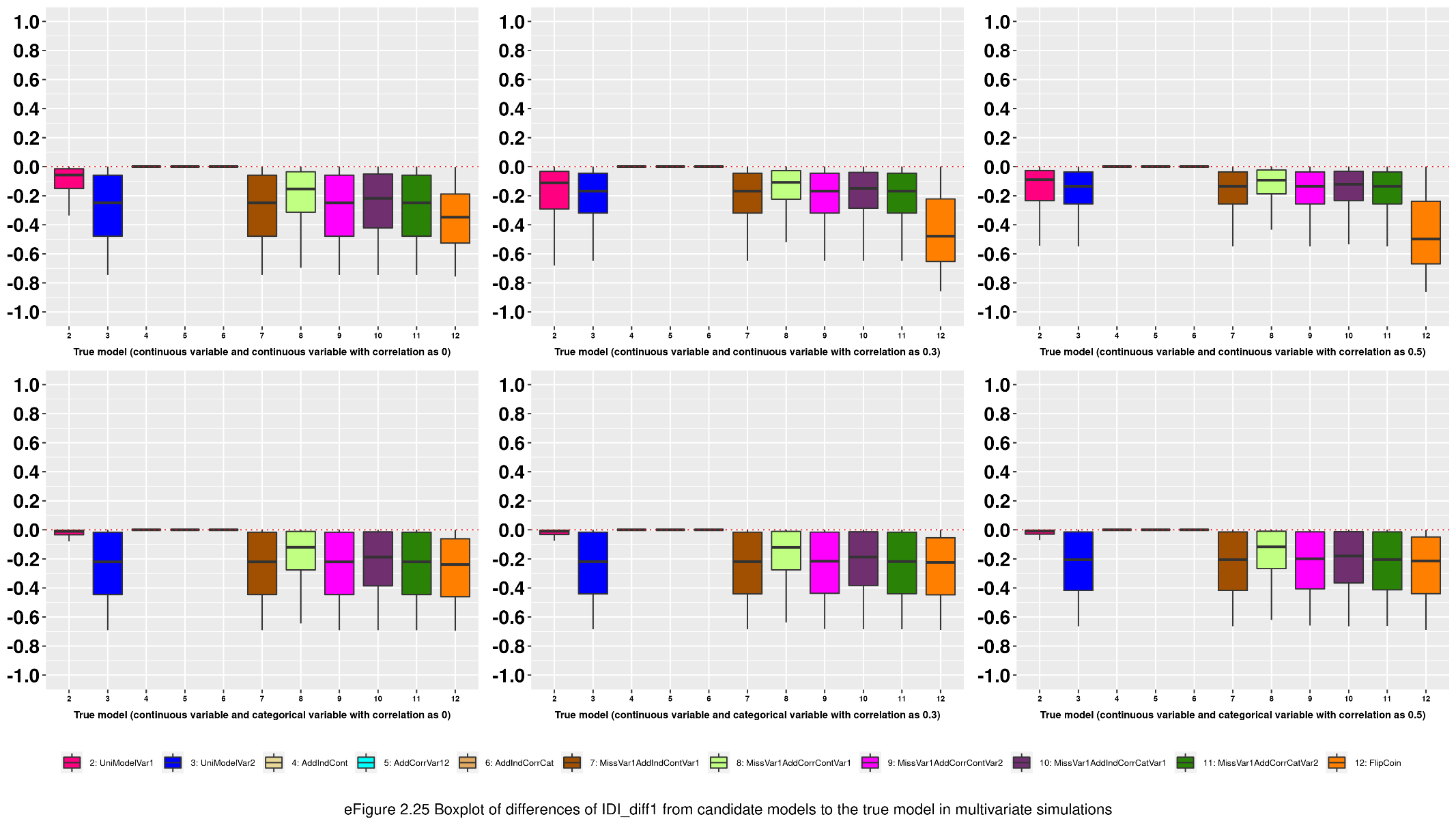


**References**

1. Hjalmarson Å. Heart rate: an independent risk factor in cardiovascular disease. *Eur Hear J Suppl*. 2007;9(suppl_F):F3-F7. doi:10.1093/EURHEARTJ/SUM030

2. Yoshioka G, Tanaka A, Goriki Y, Node K. The role of albumin level in cardiovascular disease: a review of recent research advances. *J Lab Precis Med*. 2023;8(0):7-7. doi:10.21037/JLPM-22-57

3. Kunutsor SK, Spee JM, Kieneker LM, et al. Self-reported smoking, urine cotinine, and risk of cardiovascular disease: Findings from the PREVEND (Prevention of Renal and Vascular End-Stage Disease) prospective cohort study. *J Am Heart Assoc*. 2018;7(10). doi:10.1161/JAHA.118.008726/SUPPL_FILE/JAH33124-SUP-0001-TABLES1.PDF

4. Khaw KT, Wareham N. Glycated hemoglobin as a marker of cardiovascular risk. *Curr Opin Lipidol*. 2006;17(6):637-643. doi:10.1097/MOL.0B013E3280106B95

5. de Ferranti SD, Rifai N. C-reactive protein: a nontraditional serum marker of cardiovascular risk. *Cardiovasc Pathol*. 2007;16(1):14-21. doi:10.1016/j.carpath.2006.04.006

6. Liu H, Ding C, Hu L, et al. The association between AST/ALT ratio and all-cause and cardiovascular mortality in patients with hypertension. *Medicine (Baltimore)*. 2021;100(31):e26693. doi:10.1097/MD.0000000000026693

7. Nagai M, Hoshide S, Kario K. Sleep Duration as a Risk Factor for Cardiovascular Disease- a Review of the Recent Literature. *Curr Cardiol Rev*. 2010;6(1):54. doi:10.2174/157340310790231635

8. Hippisley-Cox J, Coupland C, Brindle P. Development and validation of QRISK3 risk prediction algorithms to estimate future risk of cardiovascular disease: prospective cohort study. *Bmj*. 2017;2099(May):j2099. doi:10.1136/bmj.j2099

9. Austin PC, Steyerberg EW. Predictive accuracy of risk factors and markers: a simulation study of the effect of novel markers on different performance measures for logistic regression models. *Stat Med*. 2013;32(4):661-672. doi:10.1002/SIM.5598

10. Sigmoid Function - an overview | ScienceDirect Topics. Accessed August 11, 2023. https://www.sciencedirect.com/topics/computer-science/sigmoid-function

11. Logit Model - an overview | ScienceDirect Topics. Accessed August 11, 2023. https://www.sciencedirect.com/topics/economics-econometrics-and-finance/logit-model

12. Steyerberg EW. *Clinical Prediction Models. Statistics for Biology and Health. 2nd Edition*.; 2019.

13. Pencina MJ, D’Agostino RB, Song L. Quantifying discrimination of Framingham risk functions with different survival C statistics. *Stat Med*. 2012;31(15):1543-1553. doi:10.1002/sim.4508

14. Somers RH. A New Asymmetric Measure of Association for Ordinal Variables. *Am Sociol Rev*. 1962;27(6):799. doi:10.2307/2090408

15. Harrell , FE. Regression Modeling Strategies. Published online 2015. doi:10.1007/978-3-319-19425-7

16. Gneiting T, Raftery AE. Strictly Proper Scoring Rules, Prediction, and Estimation. Published online 2007. doi:10.1198/016214506000001437

17. Riley RD, Snell KIE, Ensor J, et al. Minimum sample size for developing a multivariable prediction model: PART II - binary and time-to-event outcomes. *Stat Med*. 2019;38(7):1276-1296. doi:10.1002/SIM.7992

18. Magee L. R 2 Measures Based on Wald and Likelihood Ratio Joint Significance Tests. *Am Stat*. 1990;44(3):250. doi:10.2307/2685352

19. Hippisley-Cox J, Coupland C, Brindle P. Development and validation of QRISK3 risk prediction algorithms to estimate future risk of cardiovascular disease: prospective cohort study. *BMJ*. 2017;357(3):j2099. doi:https://doi.org/10.1136/bmj.j2099

20. rms package - RDocumentation. Accessed August 11, 2023. https://www.rdocumentation.org/packages/rms/versions/6.7-0

21. lowess function - RDocumentation. Accessed June 26, 2022. https://www.rdocumentation.org/packages/gplots/versions/3.1.3/topics/lowess

22. Van Hoorde K, Van Huffel S, Timmerman D, Bourne T, Van Calster B. A spline-based tool to assess and visualize the calibration of multiclass risk predictions. *J Biomed Inform*. 2015;54:283-293. doi:10.1016/J.JBI.2014.12.016

23. Tjur T. Coefficients of Determination in Logistic Regression Models—A New Proposal: The Coefficient of Discrimination. *https://doi.org/101198/tast200908210*. 2012;63(4):366-372. doi:10.1198/TAST.2009.08210

24. Veall MR, Zimmermann KF. Evaluating Pseudo-R2’s for binary probit models. *Qual Quant 1994 282*. 1994;28(2):151-164. doi:10.1007/BF01102759

25. Efron B. Regression and ANOVA with zero-one data: Measures of residual variation. *J Am Stat Assoc*. 1978;73(361):113-121. doi:10.1080/01621459.1978.10480013

26. McKelvey RD, Zavoina W. A statistical model for the analysis of ordinal level dependent variables. *https://doi.org/101080/0022250X19759989847*. 2010;4(1):103-120. doi:10.1080/0022250X.1975.9989847

27. Van Houwelingen JC, Le Cessie S. Predictive value of statistical models. *Stat Med*. 1990;9(11):1303-1325. doi:10.1002/SIM.4780091109
